# Abstracts from the 1st Journal of Pharmaceutical Policy and Practice (JoPPP)-Borneo International Pharmaceutical Conference, Sarawak, Malaysia 20–22 September 2024: Publication of this supplement is sponsored by Sarawak Health Pharmacy Allied Association. Edited by Kamarudin Ahmad, Samuel Ting Chuo Yew, Nur Liyana Zainal Bahrin and Zaheer-Ud-Din Babar

**DOI:** 10.1080/20523211.2024.2403936

**Published:** 2024-09-18

**Objectives:** This study aims to define the optimal dosage of sulbactam for treating *Acinetobacter baumannii* in pediatric patients (aged 1–12 years) using a Monte Carlo simulation.

**Methods:** A systematic literature review was conducted through electronic databases such as PubMed, Scopus and Cochrane Library from 1984 to 2023 to gather the necessary pharmacokinetic parameters. A one-compartment pharmacokinetic model was performed to predict the sulbactam serum concentrations. The pharmacodynamic (PD) targets were 60% of free drug above a threshold of 0.5 to 16 times the minimum inhibitory concentration (MIC) (60%*f*T > 0.5 to 16MIC). The dose that achieved at least 80% and 90% of the probability of target attainment (PTA) was defined as optimal.

**Results and Discussion:** Thirty-six articles were included in our systematic review, and three published studies obtained the necessary parameters. The mean ± SD values of these parameters (BW = 27.14 ± 16.08 kg, CL = 5.74 ± 1.26 L/hr and Vd = 0.37 ± 0.08 L/kg) were used in Monte Carlo simulation, which simulated 10,000 patients to calculate the %PTA aiming for %*f*T > MIC > 60%. Subsequently, the optimal sulbactam dosage regimens were summarized, achieving the PD target of at least 80% and 90% of PTA. The 25 to 250 mg/kg IV regimens every 4 hours and 50 to 200 mg/kg IV every 6 hours were found to be the optimal dose for MIC 0.5 to 2 mg/L. In addition, the 150 to 200 mg/kg IV regimens every 4 hours were probably the optimal dose for MIC 4 to 8 mg/L and the regimen of 200 mg/kg IV every 4 hours was required for MIC 16 mg/L.

**Conclusions:** Pharmacodynamic targets significantly contributed to sulbactam dosing regimens in these patients. Clinical validation of the recommendation is warranted, but the safety of the mentioned doses cannot be certified. The recommended dosing regimens should be applied to patients with comparable clinical characteristics.

**Keywords:** sulbactam, pediatric patients, dose regimen, Pharmacokinetics, Pharmacodynamics, Monte Carlo simulation

