# Supplementary material for: Abstracts from the 1st Journal of Pharmaceutical Policy and Practice (JoPPP)-Borneo International Pharmaceutical Conference, Sarawak, Malaysia 20–22 September 2024: Publication of this supplement is sponsored by Sarawak Health Pharmacy Allied Association. Edited by Kamarudin Ahmad, Samuel Ting Chuo Yew, Nur Liyana Zainal Bahrin and Zaheer-Ud-Din Babar
Source: J Pharm Policy Pract. 2024 Sep 18;17(Suppl 2):2403936. doi: 10.1080/20523211.2024.2403936 (PMC11411559; doi:10.1080/20523211.2024.2403936)
Supplement: Related texts

# Abstract: Risk Factors of Central-line Associated Bloodstream Infection among Adult Patients on Parenteral Nutrition

**Authors:** Fong Pui Wun Fiona, Stella Caroline J. Bangguan, Hwang Kaai Yirng, Sharmila Seenivasagam

**Affiliations:** Department of Pharmacy, Hospital Queen Elizabeth, Sabah, Malaysia

**Objectives:** Although central venous catheter (CVC) is preferred for the administration of parenteral nutrition (PN), one of the common complications is central-line associated bloodstream infection (CLABSI), which increases mortality and healthcare burden. This study aims to determine the prevalence and risk factors of CLABSI among adult patients on PN.

**Methods:** This cross-sectional study involves all adult patients at Hospital Queen Elizabeth, Sabah, who received PN via CVC from 1st April 2022 to 31st March 2023. Potential risk factors of CLABSI were identified from medical records. Prevalence of CLABSI was calculated, and associated risk factors were determined using multiple logistic regression.

**Results and Discussion:** Of 112 patients, more than half were male (*n* = 70, 62.5%) with a mean age of 53.4 ± 15.1. Sixty-nine patients (61.6%) were from the surgical ward, while the remaining were from the intensive care unit (*n* = 25, 22.3%), medical (*n* = 9, 8.0%), haematology (*n* = 5, 4.5%), gastroenterology (*n* = 3, 2.7%) and burn wards (*n* = 1, 0.9%). The median PN duration was nine days (IQR = 5–19). CLABSI was present in 25 patients (22.3%). Of all potential factors, PN duration demonstrated a statistically significant association with CLABSI, whereby each additional day of PN via CVC increased CLABSI risk by 1.07 times (OR = 1.07; 95% CI = 1.03–1.12; *p *< 0.001). PN contributes to the risk of CLABSI due to the preference of microorganisms for dextrose, which explains the increased odds of CLABSI with prolonged PN use. Longer PN duration also means longer catheterization, exposing patients to a higher risk of infection. CLABSI may warrant withholding PN or switching to a peripheral route; necessitating reduced PN composition due to lower osmolarity cutoff and causing suboptimal nutritional status. Infection may also delay interventional procedures, increase length of stay and lead to further complications.

**Conclusions:** The duration of PN is one of the risk factors for CLABSI among PN populations. Therefore, PN duration should be minimized by evaluating the need for PN continuation daily and switching to enteral feeding as early as possible.

**Keywords**: risk factor, central-line associated bloodstream infection, adult, parenteral nutrition

# Abstract: Continuous, In Situ-Targeted, Ultrahigh Concentration of Antibiotics (CITA) for Cadiac Implantable Electronic Devices (CIED): Administration and Titration of Antibiotics.

**Authors:** Tan Sze Ling, Foo Jhi Hui, Zosimo Ken L. Jimeno, Lim Wun Xin, Stephanie Chang Yen Li, Liew Houng Bang

**Affiliations:** 1 Department of Pharmacy, Hospital Queen Elizabeth II; 2 Department of Cardiology, Hospital Queen Elizabeth II; 3 Department of Plastic and Reconstructive Surgery, Hospital Queen Elizabeth

**Objectives:** This case series aimed to describe the experience of Vancomycin and Gentamicin administration and titration, using continuous, in situ-targeted, ultrahigh concentration of antibiotics (CITA) method for treatment of cardiac implantable electronic devices (CIED) infection in Sabah Heart Centre.

**Methods:** We reviewed and summarized all the cases using the CITA method in 2023, focusing on antibiotics administration and dose titration.

**Results and Discussion:** Three cases used the CITA method in 2023. All male, aged between 35 and 90 years. Two have permanent pacemakers (PPM) and one implantable cardioverter-defibrillator (ICD). All patients empirically received intravenous (IV) Ceftriaxone and IV Vancomycin (aim level of 10–20 mg/L) and converted to CITA once cultures confirmed no growth. The dose of Vancomycin for CITA was based on the total daily dose of IV Vancomycin, which achieved a therapeutic level. For Gentamicin, a dose of 80–160 mg was given into the CIED pocket during the creation of the delivery system and then maintained with 20–40 mg daily. Both antibiotics were prepared daily in the same syringe with normal saline. TDM was done regularly with the dose titrated accordingly to ensure therapeutic for Vancomycin and a level of <1 mg/L for Gentamicin. The initial infusion rate for the first patient was set at 1.5 ml/hour (total volume 36 ml/day); however, the patient complained of CITA site pain and swelling, which then improved with the rate of 1 mL/hour. For the second case, despite the infusion rate being set at 1 ml/hour, the patient complained of CITA site pain and redness but was relieved by regular analgesics. As of the third case, an attempt of 0.8 ml/hour was made, but crystallization was noted over the infusion tubing, likely due to the high concentration of antibiotics. Subsequently, the patient tolerated 1 ml/hour with occasional bearable pain. Nonetheless, all patients could complete CITA for 10–14 days with good wound healing without significant adverse events.

**Conclusions:** CITA appears to be a promising approach in treating CIED. Individualized administration, titration, and monitoring are required to ensure optimal delivery of antibiotics via this method, where the pharmacist plays an important role.

**Keywords**: CITA, CIED, Vancomycin, Gentamicin

# Abstract: Unlocking Black Pepper’s Potential: A Nutraceutical Approach to Diabetes Management

**Authors:** Khew Choy Yuen

**Affiliations:** Department of Research, Quality Development and Innovation, Malaysian Pepper Board

**Objectives:** This study aims to investigate the potential antidiabetic and antioxidant benefits of black pepper and utilize its bioactive constituents to develop a nutraceutical for modulating blood glucose levels, ultimately contributing to the effective treatment of diabetes amidst the global epidemic.

**Methods:** The black pepper nutraceutical formulation was assessed for its inhibitory effects on key enzymes involved in carbohydrate metabolism, including β-amylase and β-glucosidase, through in vitro antidiabetic experiments. The antioxidant activity of the formulation was evaluated using the DPPH radical scavenging assay. Meanwhile, in vitro, glucose uptake experiments were conducted on cell cultures to determine the impact of the black pepper nutraceutical on glucose uptake by muscle cells and insulin sensitivity.

**Results and Discussion:** The black pepper nutraceutical formulation exhibited significant inhibitory effects on β-amylase and β-glucosidase enzymes, with a high inhibition rate of 97.3% against both enzymes. The formulation also demonstrated substantial antioxidant activity, as indicated by an observed antioxidant percentage of 85.32% in the DPPH assay. In vitro glucose uptake experiments revealed increased glucose uptake by muscle cells exposed to the black pepper nutraceutical, enhancing insulin sensitivity. These findings suggest that the black pepper nutraceutical formulation has potential as an antidiabetic agent, inhibiting key enzymes involved in carbohydrate metabolism and antioxidant properties that can reduce oxidative stress and protect cells from damage. The increased glucose uptake by muscle cells further highlights the formulation’s potential to improve insulin sensitivity, thereby contributing to better glucose management in diabetes.

**Conclusions:** Black pepper shows promise as a natural antidiabetic agent, with its nutraceutical formulation demonstrating significant inhibitory effects on enzymes involved in carbohydrate metabolism, strong antioxidant activity, and the ability to enhance glucose uptake and insulin sensitivity in muscle cells. This research lays the groundwork for further exploration of black pepper as a complementary approach to diabetes management.

**Keywords**: Black Pepper, Antidiabetic, Nutraceutical, Antioxidant, Bioactive constituents

# Abstract: The Prevalence of Unused Medicines Amongst Patients In Pengiran Anak Puteri Hajah Muta-Wakillah Hayatul Bolkiah (PAPHMWHB) Health Centre And Its Disposal Practices

**Authors:** Nurul Fatin Hazirah Fadzly, Sia Poh Geck, Nurolaini Kifli

**Affiliations:** 1 PAP Rashidah Sa’adatul Bolkiah Institute of Health Sciences, Universiti Brunei Darussalam, Brunei; 2 Department of Pharmaceutical Services, Ministry of Health, Bandar Seri Begawan, Brunei

**Objectives:** This study aims to determine the prevalence of unused medicines among patients at PAPHMWHB Health Centre, Brunei, and its disposal practices.

**Methods:** We conducted a descriptive, cross-sectional survey through face-to-face interviews using a structured questionnaire in March and April 2023. The inclusion criteria were patients aged 18 years or above and those who visited the pharmacy at PAPHMWHB Health Centre during the study period. A total of 327 participants were systematically sampled and included in the study. The chi-square and Fisher's exact tests were performed to analyze the associations between different categorical variables.

**Results and Discussion:** Our results indicate that the majority (88.4%) had unused medicines at home. The most common types of unused medicines kept were analgesics (79.9%), cough and cold medicines (58.8%) and vitamins (56.1%). Improvement in medical conditions (69.6%), forgetting to take the medicines (21.5%) and experiencing side effects of the medicines (18.7%) were the main factors contributing to the non-usage of the medicines. The majority of the participants disposed of their medication in garbage, which included solid dosage forms (64.5%), semi-solid dosage forms (77.4%), and liquid medicines (58.6%). Overall, only 20.5% of the participants practised returning unused medicines to the pharmacy through the ‘Return Your Medicines’ program. Participants who received advice regarding medicine disposal from healthcare professionals were associated with practised returning unused medicines to the pharmacy (*p *< 0.001). Participants who were aware of the program and the risks associated with improper disposal of medicines were also associated with practised returning unused medicines to the pharmacy (both associations had *p *< 0.001).

**Conclusions:** This study provides valuable insights into the high prevalence of unused medicines at home. Keeping unused medicines at home poses risks to health and the environment and represents a waste of valuable resources. Education regarding safe medicine disposal is crucial to improving the public's disposal habits. The finding underscores the necessity of interdisciplinary cooperation among researchers, pharmacists, and policymakers to raise awareness and enact policies to recycle unused medicines at home.

**Keywords**: Brunei Darussalam, Disposal, Medicine, Practices, Unused.

# Abstract: Unveiling Biosimilar Familiarity Among Diverse Medical Specialities: Insights from Physicians in Thailand

**Authors:** Ekapong Kachai, Chaoncin Sooksriwong, Wanruchada Katchamart, Tuangrat Phodha

**Affiliations:** 1 Drug Information and Consumer Protection Center, Center of Excellence in Pharmacy Practice and Management Research Unit, Faculty of Pharmacy, Thammasat University, Rangsit Campus, Pathum Thani, Thailand; 2 Division of Rheumatology, Department of Medicine, Faculty of Medicine Siriraj Hospital, Mahidol University, Bangkok, Thailand

**Objective:** This study explores the types of medical specialities associated with biosimilar familiarity among Thai physicians.

**Methods:** We conducted the research utilizing the data from the online survey. The familiarity score was evaluated by a “True/False/Don't Know” question. A correct answer was “1”, whereas an incorrect answer was “0″. Furthermore, a “don't know” answer was scored as “0” to reduce guessing bias. A one-way ANOVA was employed to analyze the association between the type of medical speciality and familiarity score (significant level at 0.05).

**Results and Discussion:** A total of 82 respondents were included in this study. There were four types of medical specialities: rheumatology, oncology, haematology, and others. The analysis revealed an association between the type of medical speciality and biosimilar familiarity score statistically significant (*p *= 0.002). The medical speciality types indicated a high impact on familiarity score (eta squared; η^2^ = 0.172). The Bonferroni post hoc test showed a statistically significant difference in the mean familiarity score, with rheumatology scoring higher than haematology (*p *= 0.038) and other medical speciality types (*p *= 0.003). Furthermore, the lowest mean familiarity score was observed among respondents from other medical speciality types. These findings emphasized the existing gap in biosimilar familiarity among Thai physicians. The initiatives aimed at enhancing biosimilar familiarity, such as the consensus on using biosimilars, the development of clinical practice guidelines, and the implementation of biosimilar educational programs, should be prioritized and disseminated across all medical specialities in Thailand.

**Conclusion:** This study provides crucial awareness of the type of medical speciality associated with biosimilar familiarity among physicians in Thailand.

**Keywords**: biosimilar, familiarity, medical specialities, physicians, Thailand

# Abstract: Exploring Clinical Outcomes of Diabetes and Hypertension Patients affected by COVID-19 pandemic: A retrospective Study

**Authors:** Jongkonnee Chongpornchai, Tuangrat Phodha, Thanawat Wongphan, Kamonwan Soonklang, Peter C. Coyte

**Affiliations:** 1 Drug Information and Consumer Protection Center, Center of Excellence in Pharmacy Practice and Management Research Unit, Faculty of Pharmacy, Thammasat University, Rangsit Campus, Pathum Thani, Thailand; 2 Trat Province Public Health Office, Ministry of Public Health, Trat, Thailand; 3 Chulabhorn Royal Academy, Bangkok, Thailand; 4 Institute of Health Policy, Management and Evaluation, Dalla Lana School of Public Health, University of Toronto, ON, Canada

**Objectives:** The study aims to compare the clinical outcomes of patients with DM and HT between the COVID-19 pandemic period and the new normal period.

**Methods:** Data was retrieved from the database of a tertiary care hospital in Thailand. The dataset includes information on patients diagnosed with diabetes, hypertension, or both during the COVID-19 pandemic period from July 1, 2021, to December 31, 2021, and the new normal period from July 1, 2022, to December 31, 2022. These patients exhibit stable symptoms without complications and do not have other comorbidities. Clinical outcome indicators, including Blood pressure (BP), fasting blood sugar (FBS) and haemoglobin A1c (HbA1c), were evaluated against the target values according to clinical practice guidelines. Chi-square tests and independent t-tests were employed for descriptive analysis of demographic characteristics. The differences in the distribution of clinical outcome indicators that met the target value between the COVID-19 pandemic and the new normal period were tested by the Chi-square statistic.

**Results and Discussion:** There were 2,193 patients included in this study: 1,018 were retrieved from the period of the COVID-19 pandemic, and 1,175 patients were retrieved from the normal period. There were statistically significant differences in the distribution of target clinical outcome indicators regarding BP (*p* < 0.05). The chi-square test results indicate that during the period of COVID-19 pandemic, there were 324 out of 1,014 patients (31.95%) with systolic BP exceeding the target value, which was higher than the 311 out of 1,165 patients (26.70%) observed during the period of new normal. In addition, during the period COVID-19 pandemic, 153 patients out of 1,014 patients (15.09%) also had diastolic BP exceeding the target value, whereas, during the period of the new normal, there were only 141 patients out of 1,165 patients (12.10%). Implementing several hospital and nationwide measures, such as limitations in patient access to treatment, changes in service delivery models, and restrictions on self-care, such as dietary control and exercise, might impact clinical outcomes.

**Conclusions:** Certain clinical outcomes, particularly blood pressure, worsened during the COVID-19 pandemic.

**Keywords**: COVID-19, clinical outcomes, diabetes, hypertension, new normal

# Abstract: Video Games Used Seriously as Medical Therapy: A Systematic Review

**Authors:** Muhammad Yaasiin bin Mansor, Siti Norlina Md Said

**Affiliations:** 1 Pharmacy Unit, Klinik Kesihatan Kuala Lumpur, 53200 Kuala Lumpur, Malaysia; 2 Department of Pharmacy Practice and Clinical Pharmacy, Faculty of Pharmacy, Universiti Teknologi MARA (UiTM), 42300 Bandar Puncak Alam, Selangor, Malaysia

**Objectives:** Serious games (SGs) are video games developed for specific uses and can be applied in the medical field to treat some medical conditions. This review aims to investigate the usability of SGs, the application of SGs, what types of SGs have been used and studied as part of medical therapy, and the medical conditions involved in the studies.

**Methods:** The Preferred Reporting Items for Systematic Review and Meta-Analysis Protocols (PRISMA-P) 2020 were adapted and provided the structure for this review. We used three databases, Web of Science, Scopus, and Cochrane, and the search terms that we used were related to serious games and medical therapy. The search terms that were used included “video game*” OR “serious game*” OR “game* used seriously” AND (therapy OR intervention OR medical), using the Boolean operators AND and OR. After retrieving an initial number of articles, further screenings were done to exclude articles that did not meet our inclusion criteria.

**Results and Discussion:** We initially retrieved 239 articles that were then screened with the title, abstract and full article, which resulted in a final number of 30 reviewed articles. This systematic review revealed that SGs in therapy were studied in conditions that involved the cognitive function and required repeated physical movements. The SGs could be used in these cases as they required the patients to use their cognitive skills or make specific physical movements to proceed with the game. The fun nature of SGs helped promote motivation and adherence and was used as a distraction against painful treatment procedures. Educational elements relating to the patient's condition were also included, especially for pediatric patients.

**Conclusions:** SGs have shown positive results when used as a complementary tool in medical therapy. SGs can provide platforms to perform physical tasks, cognitive training tasks, education and behavioural training while being fun and engaging.

**Keywords**: Video games; serious video games; games used seriously; medical therapy

# Abstract: Guideline-Directed Medical Therapy Optimization for Heart Failure with Reduced Ejection Fraction in Cardiology versus Non-Cardiology Hospitals (SARAWAK-HF Study)

**Authors:** Chelfi Chua Zhi Fei, Chung Bui Khiong, Ling Hwei Sung, Janice Wong Kee Wei, Wong Min Ting, Lian Zhi Yang, Cheah Kok Choon, Khaw Mae Jance, Chai Siew Yap, Ting Lee Yee, Noor Yuhyi Bin Sulaiman, Ting Pey Woei, Kwa Schee Li, Tan Zhun Foo, Alex Koh Zhi Yang, Wong Tze Cheng, Ho Weng Kee, Rebecca Chew Su Li, Jothiswaran Namasoo

**Affiliations:** 1 Department of Pharmacy, Sarawak Heart Centre; 2 Department of Cardiology, Sarawak Heart Centre; 3 Faculty of Medicine and Health Sciences, University Malaysia Sarawak; 4 Department of Medicine, Sarikei Hospital; 5 Department of Medicine, Limbang Hospital; 6 Department of Medicine, Kapit Hospital; 7 Department of Medicine, Serian Hospital; 8 Department of Medicine, Miri Hospital; 9 Department of Medicine, Bintulu Hospital; 10 Department of Medicine, Sri Aman Hospital; 11 Department of Medicine, Sarawak General Hospital; 12 Department of Medicine, Sibu Hospital

**Objectives:** SARAWAK-HF compared the utilization of four guideline-directed medical therapy (GDMT) pillars and clinical outcomes of heart failure with reduced ejection fraction (HFrEF) patients under heart failure (HF) clinics’ follow-up at hospital with and without cardiology speciality.

**Methods:** SARAWAK-HF is a retrospective, observational study that included HFrEF patients followed up at HF clinics in ten Sarawak hospitals from January 2021 to June 2023. This study compared GDMT optimization and clinical outcomes of patients followed up in one cardiology hospital versus nine non-cardiology hospitals over 180 days. In this study, GDMT optimization is reported as the prescription rate and number of GDMT pillars, whereas clinical outcomes include ejection fraction, mortality, and HF emergency visits or hospitalizations.

**Results and Discussion:** SARAWAK-HF included 269 HFrEF patients, with 88 patients (32.7%) from cardiology hospitals and 181 (67.3%) from non-cardiology hospitals. Over 180 days, GDMT prescription rates increased across all four drug classes in cardiology and non-cardiology hospitals. At day 180, the number of patients treated with at least three GDMT pillars in cardiology and non-cardiology centres was 66 (79.5%) and 123 (87.8%), respectively. No statistically significant difference between cardiology and non-cardiology hospitals was observed for percentage of patients on three or four GDMT pillars (*p *= 0.07). On day 180, we did not observe a significant difference in mean ejection fraction improvement between cardiology and non-cardiology hospitals (14.3% and 17.7%, respectively), with a *p*-value of 0.16. The overall 6-month all-cause mortality and HF emergency visit or hospitalization were 10 (3.7%) and 32 (11.9%), respectively, without significant difference between cardiology and non-cardiology hospitals.

**Conclusions:** SARAWAK-HF concluded that HF clinics in hospitals with or without cardiology speciality improved GDMT utilization and ejection fraction. Further studies should be conducted to study the long-term outcomes of GDMT.

**Keywords**: heart failure clinic, reduced ejection fraction, guideline-directed medical therapy, cardiology, non-cardiology

# Abstract: Monitoring and evaluation of high price medicines in the National List of Essential Medicines Category E(2) in Thailand

**Authors:** Parun Rutjanathamrong, Tuangrat Phodha, Kittiya Jantarathaneewat, Kusawadee Maluangnon, Chaoncin Sooksriwong, Pongsathorn Piebpien, Piyapat Owat

**Affiliations:** 1 Drug Information and Consumer Protection Center, Center of Excellence in Pharmacy Practice and Management Research Unit, Faculty of Pharmacy, Thammasat University, Rangsit Campus, Pathum Thani, Thailand; 2 Faculty of Medicine Ramathibodi Hospital, Mahidol University, Bangkok, Thailand

**Objectives:** Three health insurance schemes have different drug management systems, pharmaceutical benefits schemes, drug procurement, drug distribution to hospitals, and rational drug use control that impact drug access and expenditure. Monitoring and evaluation (M&E) are needed to be developed. This study aims to study the development of M&E for using essential medicine category E(2) in NLEM at the national level, a case study of Sofosbuvir /Velpatasvir with extreme amounts and Rituximab with high budget impact.

**Methods:** A mixed methods design was employed in this study. A cross-sectional study gathered the amount and value of drug utilization at four tertiary care hospitals under the Permanent Secretary Ministry Office of Public Health and four university hospitals under the Ministry of Higher Education, Science, Research and Innovation in the fiscal year of 2021 (1st October 2020 – 30th September 2021). In-depth interviews were conducted with related healthcare personnel to study criteria for prescribing Sofosbuvir /Velpatasvir and Rituximab and patients or caregivers for studying drug access.

**Results and Discussion:** The prescribing rate of Sofosbuvir/Velpatasvir, according to the criteria for essential medicines category E(2) in the NLEM, was 55%. In contrast, Rituximab was prescribed at a higher rate of 84%. The average values of Sofosbuvir/Velpatasvir per patient ranged from 593.03 to 819.75 USD, with a per-visit between 265.41 and 378.91 USD. For Rituximab, the average values per patient ranged from 1,979.09 to 3,550.82 USD, with a per-visit between 381.77 and 737.91 USD. (34.0908 THB = 1 USD on 30th September 2021)

**Conclusions:** Monitoring and evaluating essential medicines in category E(2) of the NLEM can be effectively done using hospital databases. This includes assessing prescribing practices, patient compliance with treatment plans, follow-up on clinical outcomes, and the financial value of drug use. However, for a more comprehensive analysis of drug access, it is essential to link hospital databases with healthcare insurance data.

**Keywords**: access, high-price medicines, monitoring and evaluation, essential medicines, Thailand

# Abstract: Acceptance of Electronic Prescription System among Community Pharmacists in Sarawak: A Preliminary Study

**Authors:** Loo Shing Chyi, Mas Aniza Abdullah, Yii Ee Ming, Tan Meng Hsiung, Luqman Nulhakim Said

**Affiliations:** Pharmacy Enforcement Branch Sarawak, Sarawak State Health Department, Ministry of Health Malaysia

**Objective:** This study aimed to explore CPs’ acceptance and perceptions of EPS and its associated factors.

**Methods:** A cross-sectional study was conducted from January to March 2021. All registered resident CPs were included, except those solely involved in wholesale and locum. A questionnaire based on the Technology Acceptance Model, adapted from the literature, was validated through content validation, pre-testing, and pilot testing. Internal consistency for perceived usefulness and ease of use questions was high (0.97 and 0.89, respectively). A score above 7 indicated high acceptance. Invitations were emailed, and the questionnaire was self-administered via Google Forms.

**Results and Discussion:** Among 372 CPs invited, 69 responded (18.5%). Perceived acceptance towards EPS was high (mean score 7.80 ± 2.24) despite most being non-EPS users (87%). Factors affecting acceptance included perceived usefulness (b = 0.118; 95% CI, 0.075; 0.160; *P *< 0.001), perceived ease of use (b = 0.123; 95% CI, 0.030; 0.217; *P *= 0.011), intention of future use or continued use of EPS (b = 1.884; 95% CI, 0.985; 2.782; *P *< 0.001), and number of prescriptions (>10) received annually (b = 0.760; 95% CI, 0.113; 1.407; *P *= 0.022). The widespread acceptance of EPS aligns with the current government’s policies to transition towards digitalization, reflecting a concerted effort to modernize healthcare practices that could serve as an initial step towards dispensing separation.

**Conclusion:** Acceptance of EPS is high among CPs, although most are non-users. CPs perceive EPS as valuable and easy to use, with intentions to use or continue using EPS, particularly among those who received low prescription volumes.

**Keywords:** Acceptance, Perception, Electronic Prescription System, Community Pharmacists

# Abstract: Prevalence and Associated Factors of Lipohypertrophy among Patients with Diabetes Mellitus Receiving Insulin Injections in the Districts of Sarawak

**Authors:** Loo Shing Chyi, Jessie Chin Ying Han, Wong Anak Ubong

**Affiliations:** 1 Sarawak Pharmacy Enforcement Branch, Jabatan Kesihatan Negeri Sarawak, Lorong Diplomatik 3, Petra Jaya, 93050 Kuching, Sarawak; 2 Sri Aman Health Clinic, Jalan Bukit Tembak, 95000 Sri Aman Sri Aman, Sarawak; 3 Selangau Health Clinic, 96000 Selangau, Sarawak

**Objectives:** Determined the prevalence of LH among insulin injection users and its associated factors. It also evaluates patients’ knowledge, perception, and practice of insulin injection rotation techniques.

**Methods:** A cross-sectional study recruited patients with diabetes mellitus receiving insulin injections for more than six months, from June 2022 to October 2022, across eight hospitals and seven health clinics in Sarawak. The presence of LH was assessed through inspection and palpation of insulin injection sites by medical officers at respective facilities. Self-developed questionnaires, validated for face and content, and a pilot study showing Cronbach’s alpha of 0.732 and 0.795 were employed to evaluate patient perception and practice towards insulin injection rotation.

**Results and Discussion:** A total of 355 participants participated in the study. The prevalence of LH was 5.97%. Patients exhibited good mean knowledge scores (5.09 ± 1.00), perception scores (17.38 ± 3.29), and practice scores (20.17 ± 3.86), yet the majority (86.8%) had poorly controlled HbA1c (>7%). Lower practice scores (10 points) were associated with a 7.3-fold increase in the likelihood of developing LH [OR = 0.730; 95% CI (0.643; 0.829); *P *< 0.001], which suggests that patients may perceive compliance by rotating injection sites randomly.

**Conclusions:** The prevalence of LH was low, with only practice scores found to be associated with LH development. Patients displayed good knowledge, perception, and practice towards insulin injection rotation techniques but poorly controlled HbA1c levels.

**Keywords:** Prevalence, Associating Factors, Lipohypertrophy, Insulin Injection, rotation technique, Diabetes Mellitus

# Abstract: Removal Rate of Online Illegal Medicinal Products and Cosmetics Advertisement during Operation Pangea 2020 to 2023 in Sarawak

**Authors:** Loo Shing Chyi, Alyia Farhana binti Muhadzir, Kiren Sharma, Alvina Kueh Li Wen, Augustine Law Foo Khiong, Nurfatin binti Mhd Rashid

**Affiliations:** Pharmacy Enforcement Branch Sarawak, Sarawak State Health Department, Ministry of Health Malaysia

**Objectives:** This study assessed the illegal advertisement removal rate across all examined online platforms during Operation Pangea. Comparisons were drawn between reported and reassessment removal rates.

**Methods:** A cross-sectional study where reported removal rates were extracted from the Pangea Report from 2020 to 2023, while the reassessed removal rates were obtained via reassessing the preserved links of illegal advertisements that were screened and reported in the Pangea Report. Illegal advertisements included advertisements of unregistered products, unnotified cosmetics, and exaggerated or prohibited medical claims. The chi-square test was used to identify factors associated with the illegal advertisement removal rate and compare the removal rate between reported and reassessed.

**Results and Discussion:** The study revealed that 75.1% of advertisements were reported successfully removed after our inquiries during Operation Pangea. A statistically significant association was found between the type of platform and advertisement removal (*P *< 0.001), with YouTube exhibiting the lowest removal rate at 21.7%. Moreover, 4.7% of advertisements reported as removed were still active (*P *< 0.001). Continuous efforts in engaging online platforms to develop awareness towards the potential harm of illegal advertisements and collaboration with online platforms are likely to improve the removal rate of illegal advertisements. In addition, reassessment of the reported removed advertisement can improve report accuracy.

**Conclusions:** Our findings demonstrate a commendable advertisement removal rate. However, a notable percentage of advertisements showed discrepancies from the Pangea report, highlighting the importance of reassessment and collaboration between online platform providers and government agencies in combating illegal advertising.

**Keywords**: Online advertisement, removal rate, illegal, medicinal products, cosmetics

# Abstract: Prevalence of Illegal Products and Cosmetics Detected by Pharmacy Enforcement Officers at Entry Points in Sarawak, Malaysia

**Authors:** Loo Shing Chyi, Alvina Kueh Li Wen, Alyia Farhana binti Muhadzir, Kiren Sharma, Augustine Law Foo Khiong, Nurfatin binti Mhd Rashid

**Affiliations:** Pharmacy Enforcement Branch Sarawak, Sarawak State Health Department, Ministry of Health Malaysia

**Objectives:** This study explored the prevalence and types of IPCs detected at all entry points in Sarawak during Operation Pangea from 2020 to 2023.

**Methods**: A cross-sectional study was conducted, and data were extracted retrospectively from the Operation Pangea database from 2020 to 2023. Relevant data were extracted to an electronic data collection form. Data was cleaned and analyzed by using quantitative analytical software.

**Results and Discussion:** A total of 301 consignments and 1006 items were screened during Operation Pangea from 2020 to 2023. IPCs were detected in 27.9% of consignments, and 6.7% of these IPCs were confiscated. The courier and entry points located in the middle zone have the highest confiscation rate (*p *< 0.001). Items reasonably believed to contain adulterants (38.8%) and unregistered health supplements (31.3%) were the most confiscated IPCs. Consequently, it has prompted the implementation of a new work process involving the inclusion of a QR code directing users to educational resources to discourage repeated importation. In addition, emphasis was placed on implementing thorough screening and physical checks, especially in the middle zone of Sarawak, to enhance the detection rate.

**Conclusions**: This study highlights the outcomes of pharmacy enforcement officers in combating the importation of IPCs in Sarawak during an international operation from 2020 to 2023. To better strategize the efforts by pharmacy enforcement branches in combating IPCs, more time series data should be collected and analyzed to explore the trend and pattern of IPCs distribution in the region.

**Keywords**: Prevalence, Illegal Products and Cosmetics, Entry Points, Operation Pangea

# Abstract: Prevalence and Trends of Adulterants Detected in Products Sampled by Sarawak Pharmacy Enforcement Branch

**Authors:** Loo Shing Chyi, Sim Sui Theng, Cornelius Chua Kim Ann, Ting Chuong Wei, Augustine Law Foo Khiong, Ridhwan bin Abdul Razak

**Affiliations:** Sarawak Pharmacy Enforcement Branch, Sarawak State Health Department, Lorong Diplomatik 3, Petra Jaya, 93050 Kuching, Sarawak

**Objectives:** This study assesses the prevalence and trends of adulterants in products sampled by the Sarawak Pharmacy Enforcement Branch (PEB).

**Methods:** A cross-sectional study in which data on all samples from January 2015 until December 2021 were extracted retrospectively. The samples were obtained from intelligence, market surveillance, and public complaints. The samples were sent to the Department of Chemistry Malaysia to be analyzed qualitatively for the presence of adulterants.

**Results and Discussion:** Out of 633 samples analyzed, the majority were traditional medicine products (54.3%), followed by health food products (31.4%), over-the-counter products (7.6%), supplements (4.9%), and others (1.7%). Adulterants were detected in approximately one-third of traditional medicine (33.1%), health food (39.2%), over-the-counter (25.0%), and supplement products (38.7%). The most found adulterants were sex stimulants, accounting for the highest proportion of adulterated samples. Hence, more specific strategies for improving public awareness of the dangers of misuse of sex stimulants were necessary. Notably, samples obtained through intelligence activities showed a significantly higher prevalence of adulteration (*P *< 0.001), which justifies expenditure on purchasing the samples from the market to safeguard the public interest, especially in assuring the safety of the products marketed in Malaysia.

**Conclusions:** The study revealed that nearly one-third of the samples were adulterated, particularly with sex stimulants being the most frequently detected adulterant. Continuous strict surveillance and public awareness campaigns are essential to address this issue and safeguard public health.

**Keywords:** Adulterants, Traditional Medicines Products, Health Food Supplements, Over-the-Counter Products

# Abstract: Gap analysis on the development of legal framework for drug security in Thailand: a scoping review

**Authors:** Parun Rutjanathamrong, Chaoncin Sooksriwong, Vijj Kasemsup

**Affiliations:** 1 Drug Information and Consumer Protection Center, Center of Excellence in Pharmacy Practice and Management Research Unit, Faculty of Pharmacy, Thammasat University, Rangsit Campus, Pathum Thani, Thailand; 2 Faculty of Medicine Ramathibodi Hospital, Mahidol University, Bangkok, Thailand

**Objectives:** There is no study on developing a legal framework for drug security in Thailand. Gap analysis is required for the development of a legal framework. So, this scoping review gathers related documents for mapping gap analysis and drug security in Thailand.

**Methods:** A scoping review on “drug security” was performed by searching five databases of Thailand, including (1) Thai Journal Online, (2) Health System Research Institute (HSRI), (3) Thailand Library Integrated System (Thailis), (4) Thai National Research Repository (TNRR), and (5) Digital Research Information Center of the National Research Council of Thailand. The search terms “drug security” OR “security in medicine” (in Thai) were employed to gather the document from online databases. The published studies and government documents from 2000 to 2023 were recruited to the screening process. After initial identification, documents underwent a screening process to exclude duplicates and irrelevant materials. A gap analysis was conducted on the remaining documents to identify deficiencies in Thailand’s legal framework concerning drug security.

**Results and Discussion:** The initial search identified 471 documents, of which 186 were duplicates and 280 were deemed irrelevant. Five key documents were thoroughly reviewed. The findings indicate a heavy reliance on imported pharmaceuticals, highlighting the unsustainability of Thailand’s current pharmaceutical business model. There is a critical need for research and development in pharmaceutical supply chains and logistics systems tailored to the Thai context. Strategic evaluations and lessons learned from existing research emphasize the importance of fostering self-reliance in drug production. Additionally, following the Triple Bottom Line (TBL) principle—Economic, Social, and Environmental factors—sustainability indicators should be incorporated at the national policy level to ensure comprehensive drug security.

**Conclusions:** Significant reforms in the public health emergency management system are necessary to improve drug security in Thailand. Adjustments in the structure and roles of relevant agencies are crucial to developing a resilient and self-sufficient pharmaceutical sector. This study underscores the importance of a well-structured legal framework to support these changes and enhance national drug security.

**Keywords**: Drug security, gap analysis, law, regulation, sustainability, public health

# Abstract: Pilot study of oral sodium bicarbonate knowledge, attitude and practice questionnaire among pharmacy staff

**Authors:** Siew Mei Sim, Joan Zu En Wong, Zai Yang Yong, Jaime Yoke May Chan

**Affiliations:** 1 Department of Pharmacy, Sarawak General Hospital, Ministry of Health, Kuching, Malaysia; 2 Centre for Quality Management of Medicines, Faculty of Pharmacy, Universiti Kebangsaan Malaysia, Kuala Lumpur, Malaysia

**Objectives:** Pharmacists play a crucial role in patient education and medication counselling to improve patients’ adherence, particularly in those with high pill burdens, such as individuals with chronic kidney disease (CKD). However, effective pharmacist-led patient education requires good core knowledge of disease and treatment. This study evaluated the validity and reliability of the oral sodium bicarbonate knowledge, attitude and practice (KAP) questionnaire for pharmacy staff.

**Methods:** A 23-item questionnaire consisting of three domains, knowledge, attitude and practice, was constructed and tested. This study was conducted in two phases: the content validity by an expert panel and the pilot study. Content Validity Index (CVI) was used to determine the validity of the questions quantitatively, and two types of CVI were evaluated: item-level CVI (I-CVI) and scale-level CVI (S-CVI). I-CVI and the average I-CVI scores (S-CVI/Ave) were calculated. I-CVI ≥ 0.78 and S-CVI/Ave ≥ 0.90 were considered high content validity. For the pilot study, the questionnaire was administered via Google Forms to outpatient department pharmacy staff. The reliability of the questions in each domain was established using Cronbach's alpha measurement. A Cronbach's alpha value of ≥0.70 indicated adequate internal consistency.

**Results and Discussion:** This questionnaire showed high content validity of individual items in each of the domains (knowledge domain, I-CVI range: 0.67–1.00; attitude domain, I-CVI: 1.00; practice domain, I-CVI range: 0.83–1.00) and high overall content validity for all three domains (Knowledge domain, S-CVI/Ave = 0.91; Attitude domain, S-CVI/Ave = 1.00; Practice domain, S-CVI/Ave = 0.95). The pilot study was conducted among 30 pharmacy staff. Most respondents were female (*n* = 23, 76.6%) and fully registered pharmacists (*n* = 21, 70.0%). Cronbach's alpha values for the reliability of the knowledge, attitude, and practice domains were 0.755, 0.925, and 0.676, which shows adequate internal consistency for all domains.

**Conclusions:** This pilot study showed that the developed questionnaire was feasible for the proposed main study with the intended objectives.

**Keywords:** oral sodium bicarbonate, knowledge, attitude, practice

# Abstract: The monetary benefits of various managed entry agreements for access to anticancer drugs: A Systematic Review

**Authors:** Piyapat Owat, Chaoncin Sooksriwong, Hataiwan Ratanabunjerdkul, Tuangrat Phodha

**Affiliations:** 1 Drug Information and Consumer Protection Center, Center of Excellence in Pharmacy Practice and Management Research Unit, Faculty of Pharmacy, Thammasat University, Rangsit Campus, Pathum Thani, Thailand; 2 Medical Oncology Unit, Department of Internal Medicine, Faculty of Medicine, Thammasat University, Rangsit Campus, Pathum Thani, Thailand

**Objectives:** This study aims to review and describe the monetary benefits of various managed entry agreements (MEAs) utilization on access to anticancer drugs.

**Methods:** A systematic review was conducted through PubMed, ScienceDirect, The Cochrane Library, and EconLit. The literature search was performed for articles published from January 1, 2000, to March 30, 2023. Articles published in English that describe the monetary benefits of MEAs implemented in any country to facilitate access to anticancer drugs for cancer patients were included. Magazines, letters, and news were excluded. Data were analyzed using thematic analysis.

**Results and Discussion:** In this study, two themes of MEAs were identified: (1) improving the incremental cost-effectiveness ratio (ICER) to provide data for decision-making regarding the recommendation of drugs for inclusion in the patient access scheme (PAS) and (2) drug cost savings to sustain the budget. The United Kingdom has implemented MEAs for anticancer drugs, especially discount and rebate agreements. These agreements have improved ICER to less than £30,000 per quality-adjusted life-year (QALY). Other countries have implemented MEAs to save on drug costs. In Brazil, a discount agreement has been implemented for erlotinib, resulting in annual drug cost savings of 30 million Brazilian reais. In Italy, a cost-sharing agreement has been implemented for erlotinib, resulting in drug cost savings of €25 million. In the Netherlands, a pay-by-result agreement has been implemented for erlotinib, resulting in drug cost savings of €6,626 per case. Additionally, in Spain, a pay-by-result agreement has been implemented for gefitinib, resulting in drug cost savings of around €36,000. Lastly, a discount agreement has been implemented for sunitinib in the United Kingdom, resulting in cost savings of £3,396,398. Although MEAs are an effective strategy and should be applied, further research should be conducted when implementing them in different countries with varying healthcare systems.

**Conclusions:** The objectives of MEAs implementation in various countries are (1) improving the ICER to provide data for decision-making regarding the recommendation of drugs for inclusion in the PAS and (2) cost savings to sustain the budget.

**Keywords:** managed entry agreements, anticancer, monetary benefits, access, budget

# Abstract: Impact of COVID-19 towards antibiotic consumption in a major specialist hospital: A non-COVID-19 hospital perspective

**Authors:** Laura Soon, Darren Stacey Simon, Foo Seng Ling, Michele Xing Yi Ng, Anith A Aziz, Qing Liang Goh, Kah Ling Fong, Nur Hazwani Bt. Zulbadrisham

**Affiliations:** 1 Pharmacy Department, Hospital Queen Elizabeth II, Sabah, Malaysia; 2 Pathology Department, Hospital Queen Elizabeth, Sabah, Malaysia

**Objectives:** This study aimed to compare the prevalence of antibiotic consumption before (2018/2019) and during (2020/2021) the pandemic and to explore its impact on antibiotic-acquired cost and bacterial resistance.

**Methods:** This observational study reviewed antibiotic consumption from 1st January 2018 to 31st December 2021 in HQEII. The antibiotics selected were Meropenem, Vancomycin, Piperacillin-tazobactam, Ceftazidime and Ceftriaxone. The antibiotic consumption, antibiotic-acquired cost and cases of multidrug-resistant organisms (MRO) before (2018/2019) and during (2020/2021) the COVID-19 pandemic were compared.

**Results and Discussion:** The overall consumption of the selected antibiotics significantly increased by 45.2% (*p *< 0.001) during the COVID-19 pandemic. The intensive care unit had the highest increase in antibiotic consumption (+114.3%, *p *< 0.001). There was a rising trend for using Vancomycin, Meropenem, Ceftazidime and Piperacillin-Tazobactam (*p *< 0.005). All these contributed to a significant increase in antibiotic-acquired cost by 64.4% during the COVID-19 pandemic (RM909,898.80 vs RM1,486,791.20, *p *< 0.001). Notably, cases of multidrug-resistant organisms also increased, especially MRO Acinetobacter (+197%) and Carbapenem-resistant Enterobacterales (+92%). During the pandemic, HQEII was designated the main non-COVID-19 referral centre on the west coast of Sabah. There was also an interruption of the Antimicrobial Stewardship service, carried out twice weekly, and the round was withheld from March 2020 until October 2021. These may lead to an increase in antibiotic consumption during the pandemic.

**Conclusions:** High antibiotic consumption, antibiotic-acquired cost and MRO cases were observed in non-COVID-19 healthcare settings during the pandemic. Our suggestion for future studies would be to include the factors of antibiotic consumption and the bacterial resistance pattern.

**Keywords**: Antibiotic consumption; Antibiotic cost; Bacterial resistance; COVID-19 pandemic

# Abstract: The price reduction pattern of innovators from applying the reference pricing policy

**Authors:** Chaoncin Sooksriwong, Siriwat Suwattanapreeda, Kusawadee Maluangnon, Sanita Hirunrassamee, Thirapich Chuachantra, Krissana Kuchaisit, Niti Osirisakul

**Affiliations:** 1 Drug Information and Consumer Protection Center, Center of Excellence in Pharmacy Practice and Management Research, Faculty of Pharmacy, Thammasat University, Rangsit Campus, Pathum Thani, Thailand; 2 Somdet Chaopraya Institute of Psychiatry, Bangkok, Thailand; 3 Medicines Regulation Division, Food and Drug Administration, Ministry of Public Health, Nonthaburi, Thailand; 4 Pharmacy Division, Faculty of Medicine Ramathibodi Hospital, Bangkok, Thailand

**Objective**s: Reference drug pricing is one of the government measures applied to the public procurement system to reduce prices. This research aims to identify the pattern and magnitude of price reductions resulting from the reference drug pricing measure applied to innovator drugs.

**Methodology:** The public hospitals procurement database from The Controller General Department, Ministry of Finance from 2018 to 2022 was retrieved. The stratified sampling method was used for sample selection. By commencing ten pharmacological groups of the highest procurement budgets, 8 GPUs from each group were selected. 81 GPUs (588 TPUs) were sampled, and 75,755 transactions were identified from 923 hospitals in Thailand.

**Results and Discussion:** The study examined 23 groups of originator drugs, with reference prices (RP) announced between 2018 and 2022. These drugs were categorized into two groups: (A) monopoly originators and (B) originators with generic competition. Group A: 27% have RP higher than, 18% have RP equal to, and 55% have RP lower than the median of previous procured prices. The median price decreased to 75.50% of the previous year’s price (range 21.27% to 93.46%). Despite the price reductions, all innovator drugs remained on the market. Group B: Among this group, 16.67% were removed from the market after the announcement of RPs. The RPs announced were 49.05 and 62.76% of previous innovators sold prices. The rest, or 83.33%, stayed in the market and reduced the price close to RPs to compete with generics. The median price was 74.36% of the year before (range 70.00% to 100.00%). Some innovators kept decreasing prices to compete with generic drugs to the price of 33.40% to 66.92% of before.

**Conclusions:** Applying a reference price policy can reduce innovators’ prices to 75% of the previous year’s prices, whether having generics or not. However, innovator prices could reduce more to compete with generic drugs as time passed. The figure 75% could be used for price negotiation with innovators because all remained in the market.

**Keywords:** drug price policy, reference drug prices, drug price reduction, price reduction pattern

# Abstract: An Observational Study to Compare Accuracy of Soluble Insulin Delivered via INSUPen Pro and a Comparator

**Authors:** Tan Jun Hua, Winnie Too, Chua Kheng Sin Harry, Chuo Sing Hong

**Affiliations:** 1 Pharmacy Department, Sarikei Hospital, Sarikei, Sarawak, Malaysia; 2 Sarikei Divisional Pharmacy Office, Sarikei, Sarawak, Malaysia; 3 Pharmacy Department, Kanowit Hospital, Kanowit, Sarawak, Malaysia

**Objectives:** This study aims to explore alternative safe methods for accurate insulin dose delivery in the event of insulin penfill cartridge supply disruption affecting a particular brand in a Malaysian public hospital setting. Specifically, this study investigates the accuracy of Insugen®-R delivered between the comparator insulin pen (Allstar® pen) against the control (INSUPen PRO™).

**Methods:** Out of all the insulin pens currently available in the Malaysian market, the Allstar® pen was chosen as a comparator because it can accommodate Insugen® penfill cartridges. Soluble insulin (Insugen®-R) was chosen because of its relative uniformity and less sedimentation prone. New cartridges from the same batch and new 32G needles were used for each test. The weight of insulin delivered was measured gravimetrically using an electronic analytical balance by sequential deduction of insulin pen weight pre- and post-plunger actuation of varying international units (1-unit, 2-unit, 6-unit and 10-unit). Ten repetitions were performed for each unit according to ISO Standard 8655-6:2022. The data collected was analysed via an independent-sample t-test of the mean difference in insulin weight delivered by both insulin pens.

**Results and Discussion:** Eighty measurements were obtained (two pens, four doses with ten iterations each). All the tests passed Levene’s test for equality of variances (*p* > 0.05). No statistically significant difference in weight for actuation of 1-unit (t(18) = 0.287, *p *= 0.777), 2-unit (t(18) = −0.249, *p *= 0.806), 6-unit (t(18) = −1.672, *p *= 0.112) and 10-unit (t(18) = −1.383, *p *= 0.183) was found. Further testing using intermediate-acting NPH insulin (Insugen®-N) and premixed insulin (Insugen® 30/70) may be required to confirm accuracy.

**Conclusion:** The weight of Insugen®-R delivered from both pens was not statistically different. This study suggested that the Allstar® pen may be an alternative for accurate insulin dose delivery. We recommend future studies on users’ acceptance and preference of insulin pens.

**Keywords**: soluble insulin, insulin pen, INSUPen Pro, AllStar pen, accuracy

# Abstract: Comparison of Medication and Surgical Items Procurement Prices under Local Tender vs Direct Purchase Method at Sarikei Divisional Pharmacy Office

**Authors:** Ung Stephanie Kee Yiin, Chua Harry Kheng Sin, Tiong Lilian Chyong Shin, Teng Wan Ying, Leong Fei Lin

**Affiliations:** Pejabat Farmasi Bahagian Sarikei

**Objectives:** This study explored the difference in procurement prices of medication and surgical items between local tender and direct purchase methods.

**Methods:** A comparative observational study was conducted in July 2023. The samples that contributed a significant portion of the overall procurement value of local tender for the first half of 2023 in the Sarikei Divisional Pharmacy Office were selected from the list. Thirty-nine medications and five surgical items procurement prices were compared between local tender and direct purchase methods. Descriptive analyses and independent t-tests were conducted using SPSS to analyse the data.

**Results and Discussion:** Out of 39 medications, 25 have lower local tenders prices than direct purchases. Among 25 medications, the considerable price difference was 55.72% cheaper. Conversely, 3 out of 5 surgical items have higher prices in local tenders. However, there was no significant difference in the mean procurement prices of medications and surgical items between the local and direct procurement methods. (*p *= 0.924).

**Conclusions:** More studies are needed to explore optimising procurement strategies, enhancing cost-effectiveness, and ensuring equitable access to essential medicines in Sarawak.

**Keywords**: Local Tender, Direct Purchase, Comparison, Medicine price

# Abstract: A Cross-Sectional Survey to Evaluate the Perception of Drugs and the Development of Phlebitis among Nurses in Malaysia

**Authors:** Jerry Liew Ee Siung, Wan Nurul Aishah Binti Wan Mahmood, Carolyn Teo Ai Li, Ho Shing Yang, Anith Binti A. Aziz, Lim Ming Jie, Calvin Sim Siau Xin, Kuapih @ Florina Kimjun, Benjamin Loh Chuan Ching

**Affiliations:** Department of Pharmacy, Queen Elizabeth Hospital, Sabah, Malaysia

**Objectives:** Intravenous drug treatment is a primary part of nursing practice. The nurses should possess the required knowledge to recognise drugs’ effect on phlebitis. This study aimed to evaluate nurses’ perception of the risk of phlebitis for drugs commonly used in the wards.

**Methods:** A cross-sectional survey was conducted among ward nurses at Queen Elizabeth Hospital (March-April 2024) using a self-administered questionnaire form. All nurses actively involved in patient care with at least six months of experience were recruited in this study. Ward nurses were stratified by type of wards and having post-basic certificates before they were selected by simple random sampling. Descriptive statistics analysed data obtained.

**Results and Discussion:** The majority of the study participants (*n* = 312) were female (*n* = 280, 89.7%), with a mean age of 34.9 years (SD = 4.5). 28.5% (*n* = 89) had post-basic education, and the average nursing experience was 10.2 years (SD = 3.8). Slightly more than half of nurses (*n* = 184, 59%) agreed that drugs can be the cause of phlebitis. However, a third (*n* = 99, 31.8%) of all nurses perceived that drugs were less likely to cause phlebitis than other risk factors. 22.8% (*n* = 71) believed that there was no difference between drugs in causing phlebitis. A total of 16 drugs (38%) were misperceived in terms of phlebitis risk.

**Conclusions:** This study demonstrates nurses’ differing perceptions about drugs and the risk of phlebitis. While most nurses agree that drugs can cause phlebitis, approximately one-third believe they are less likely to cause phlebitis than other established risk factors. Drugs were also mistakenly believed to carry a comparable risk of phlebitis. As a result, efforts should be undertaken to provide continuous education to nurses about the risk of phlebitis with routinely used drugs.

**Keywords**: Phlebitis, Drugs, Risk, Wards, Routine, Survey

# Abstract: A Before and After Study to Evaluate the Effect of Pharmacist-led Intervention to Reduce Overstocking of Antidiabetic Medications among Patients in District Health Clinic in Malaysia.

**Authors:** Ka Mun Ho, Shu Ning Lee, Jerry Ee Siung Liew

**Affiliations:** 1 Department of Pharmacy, Klinik Kesihatan Apas Balung, Sabah, Malaysia; 2 Department of Pharmacy, Klinik Kesihatan Tawau, Sabah, Malaysia; 3 Department of Pharmacy, Hospital Queen Elizabeth, Sabah, Malaysia

**Objectives:** This study compares the cost, medication adherence, and glycemic control of diabetic patients in a pharmacist-led intervention to prevent antidiabetic medication overstocking.

**Methods:** A quasi-experimental (before-after) study was adopted among diabetic patients who presented for their monthly medication refill. During the adaptation phase (3 months), the patient was educated and guided in filling out the balance counting form, and drugs were dispensed according to routine practice. According to the medication balance counting form, only a sufficient number of medications were dispensed during the intervention phase (following three months). The total costs of antidiabetic medications, glycosylated haemoglobin (HbA1c) changes, and medication adherence (pill-counting method) were accessed before and after intervention. Patients were also interviewed to determine the reasons for drug non-adherence if they were found to overstock antidiabetic medications.

**Results and Discussion:** Thirty-three patients, predominantly female (*n* = 21, 64%) with a mean age of 58.2 years (SD = 10.5), were recruited in this study. The study intervention significantly reduced the total cost of antidiabetic medications (RM 3202.4 (SD = 81.2) vs RM 2842.4 (SD = 70.7), *p *= 0.005) dispensed by 12%, which was translated to a cost saving of RM 360 (SD = 20.9). There were no significant differences in the mean HbA1c (8.3% (SD = 2.3) vs 8% (SD = 2), mean difference = 0.2%, *p* = 0.518) in this study. A total of 17 patients (52%) were found to be non-adherent to their medications. The top three reasons identified were forgetfulness about their medications, multiple follow-ups, and adverse effects.

**Conclusions:** This study supports the implementation of a balanced counting form for diabetic patients to reduce medication wastage. The intervention implemented was beneficial in reducing the medication cost without affecting the patient's glycemic control. However, a significant portion of a patient were found to be non-adherent. Hence, efforts to address the main reasons for medication non-adherence must be implemented.

**Keywords**: medication wastage, balance counting, medication adherence

# Abstract: Identifying the Issues and Challenges of Prosecution Faced by the Pharmacy Enforcement Division in Malaysia: A Qualitative Study

**Authors:** Dr. Mohammad Firdaus bin Abdul Aziz, Hii Lu Yien

**Affiliations:** 1 Faculty of Law, Universiti Malaya, Selangor, Malaysia; 2 Pharmacy Enforcement Branch, Sabah State Health Department, Sabah, Malaysia

**Objectives:** This study aims to identify the issues and challenges pharmacy enforcement officers face in conducting prosecutions.

**Methods:** A qualitative study recruited experienced pharmacy enforcement officers appointed as prosecuting officers from several states across Malaysia for face-to-face interviews. Each interview session lasted one hour, was audio-recorded, and transcribed verbatim. The tenth interview reached the data saturation point. An inductive analysis was conducted on the findings to identify relevant themes, which were then corroborated by a literature review and research supervisor.

**Results and Discussion:** Six out of ten pharmacy enforcement officers had a master’s degree in a law-related field, while the other four possessed valuable enforcement experience. Thematic analysis identified two main issues: incomplete investigations resulting in insufficient evidence, which hinders successful prosecution, and pharmacy enforcement officers struggling to present solid legal arguments due to a lack of adversarial legal skills. The identified challenges are (i) limitations in current legislation, (ii) challenges in proving cases in court due to judges presiding over the proceedings being unfamiliar with pharmacy legislation, and (iii) witnesses’ reluctance to cooperate due to fear of testifying in open court. Results indicated that the problems faced by pharmacy enforcement officers occur either at the beginning of prosecution, when receiving poor investigation papers, during the proceeding process, or post-prosecution. They identified a lack of confidence in pharmacy enforcement officers in conducting prosecutions and some uncontrollable external factors associated with current pharmacy legislation and other agencies as barriers to successful prosecution.

**Conclusions:** Pharmacy enforcement officers encounter various issues and challenges in conducting prosecutions. This study suggests mitigation strategies for the Pharmaceutical Enforcement Division, such as improving investigation procedures and prosecution practices, enhancing training for pharmacy enforcement officers and revising current pharmacy legislation. The findings also highlight the importance of effective collaboration with related agencies and educating the public and judges to ease the prosecution process.

**Keywords**: prosecution, prosecuting officers, pharmacy enforcement officer, pharmacy legislation

# Abstract: Looking into FarmaTag Hologram: Are Consumers and Regulatory Authorities Provided with Complete Pharmaceutical Product Information?

**Authors:** Ahmad Firdaus bin Shahari, Anitha Uthayakumaran, Zulhilmi Farhan bin Zulkefli, Muhamad Fikri bin Bakhtiar, Lavinia Kong Jin Qi

**Affiliations:** Pharmacy Enforcement Branch, Pharmaceutical Services Division, Sabah State Health Department, Ministry of Health Malaysia, Malaysia

**Objectives:** The FarmaTag Hologram is a mandatory QR code label on registered pharmaceutical products (RPP) in Malaysia, providing instant product information to consumers. This study aims to determine the proportion of authentic FarmaTag Holograms, assess the availability of complete product information in the FarmaChecker app, and classify the category of information provided among mainstream medicine sellers (MMS) in Sabah, Malaysia.

**Methods:** A cross-sectional descriptive study was conducted using census sampling of 1,262 MMS in Sabah. Four registered pharmaceutical products (RPP) were conveniently selected from each MMS, and their holograms were scanned using the FarmaChecker app to verify authenticity. If authentic, a button for product information would appear. The status was marked as ‘not assigned’ if the product information was incomplete. All data were collected and analyzed using the Statistical Package for Social Sciences (SPSS).

**Results and Discussion:** The results of this study provide significant insights into the completeness of product information and the classification category of RPP in Sabah State, Malaysia. All 5,048 FarmaTag Holograms on RPPs were authentic (100%). However, 94.7% (*n* = 4,780) of the product information was not assigned. This deficiency is primarily attributed to the optional policy of manufacturers, distributors, or suppliers filling in the information (MDS). The voluntary level of MDS in providing complete product information is a significant issue. Among the remaining 5.3% (*n* = 268), 47.8% (*n* = 128) were classified as ‘Over-the-Counter,’ while 52.2% (*n* = 140) were designated as traditional products. None were categorized as scheduled poisons or supplements, meaning all poison and supplement products available in private clinics, hospitals and pharmacies lack product information. It is deeply concerning because the provision of product information is beneficial for both consumers and regulatory authorities.

**Conclusions:** The availability of product information is significantly low. The voluntary nature of this process underscores the need for greater cooperation from manufacturers, distributors, and suppliers in providing complete information. It is suggested that this process be made mandatory to improve results. Providing complete information benefits consumers and regulatory authorities by enhancing oversight and ensuring safety standards in the industry.

**Keywords**: FarmaTag hologram, registered pharmaceutical products, complete product information, product classification category, Sabah, Malaysia

# Abstract: Cross-Border Region Pharmaceutical Crime through Sebatik Island: Analysis and Challenges

**Authors:** Joseph Oyol Modili, Mohd Afiq Bin Abu Latifei, Ooi Kah Geap, Wong Jing Ting, Thai Yih Yang, Farisha Binti Fauzi, Rashidatul Asmin Binti Rosli

**Affiliations:** Pharmacy Enforcement Branch, Sabah State Pharmaceutical Services Division, Ministry of Health Malaysia

**Objectives:** This study aims to determine the crime rate, types, and direction of transportation of illegal pharmaceuticals and cosmetics across the Sebatik border, which is well-known for the trafficking of controlled goods, drugs, and other commodities.

**Method:** We analyzed police seizure data from 2021 to identify trends in the illegal transportation of pharmaceuticals and cosmetics across the Sebatik border. The analysis focused on seized items’ frequency, types, values and transportation direction (importation versus exportation).

**Results and Discussion:** Our results indicate that ineffective border control has led to an increasing trend of illegal transportation of pharmaceuticals and cosmetics. The crime rate was determined to be 0.23 per 100,000 people in 2021, 1.83 in 2022, and 2.05 in 2023. Of the total value of seized items, 72.73% comprised unnotified cosmetics, while the remaining 27.27% were from unregistered medicines, reflecting a more robust demand for the former. Moreover, 51.82% of cases were suspected of illegal exportation, while the remaining 48.18% were suspected of illegal importation, indicating a balanced and active bidirectional smuggling environment with organized criminal activities on both sides of the smuggling trade. It also indicates that Malaysia is both a point of transit and a destination for illegal goods.

**Conclusions:** This study underscores the necessity to implement a risk-based enforcement approach aligned with regional crime patterns by expanding awareness efforts, collaborating with domestic and international stakeholders, strengthening border controls, and improving regulatory frameworks.

**Keywords**: Pharmaceutical crime, Sebatik Island, risk-based enforcement approach, border control, police seizure.

# Abstract: Modification and cross-cultural adaptation of MedUseQ in primary healthcare settings in Penang, Malaysia

**Authors:** Christina Malini Christopher, Ali Blebil, Bhuvan KC, Deepa Alex, Mohamed Izham Mohamed Ibrahim, Mark Cheong Wing Loong

**Affiliations:** 1 Hospital Sultanah Bahiyah, Alor Setar, Ministry of Health Malaysia; 2 School of Pharmacy, Monash University Malaysia, Jalan Lagoon Selatan, Subang Jaya, Selangor, Malaysia; 3 School of Clinical Sciences, Queensland University of Technology, Brisbane, Australia; 4 Jeffrey Cheah School of Medicine and Health Sciences, Monash University Malaysia, Jalan Lagoon Selatan, Subang Jaya, Selangor, Malaysia; 5 Clinical Pharmacy and Practice Department, College of Pharmacy, QU Health, Qatar University, Doha, Qatar

**Objectives:** Assessing medication utilization patterns among older adults is crucial for identifying the barriers and challenges they encounter in primary healthcare. However, limited tools can be used specifically for the older population. This study aims to modify and culturally adapt Malaysia's MedUseQ questionnaire (English version).

**Methods:** The target population was older adults above 60 years in **General Medical Wards** settings in Penang. Participants were selected to ensure a balanced demographic profile encompassing individuals from diverse socio-economic and educational backgrounds. Methodological approaches started with cognitive debriefing, followed by content validation by two experts, pilot testing and reliability analysis.

**Results and Discussion:** The main issues identified in the cognitive debriefing and content validation were lengthy questions, difficulties in understanding certain words such as ‘community pharmacy’ and ‘primary healthcare’ and sensitive questions on alcohol consumption. Subsequently, the questionnaire length was revised, and redundant questions were consolidated to enhance participant convenience during completion. A pilot test on 20 older adults on the overall questionnaire reported a Cronbach alpha of 0.884 (good internal consistency reliability).

**Conclusions:** This study supports the modified version of MedUseQ questionnaires to be valid and reliable. It can be used to identify medication use problems among older adults in Malaysia and assist in generating solutions for such problems.

**Keywords**: MedUseQ, Modification, OlderAdults, Validation

# Abstract: Investigating the Cost of Healthcare Clinical Waste: A Case Study in Tuanku Mizan Armed Forces Hospital

**Authors:** Manvikram Singh Gill

**Affiliations:** Department of Pharmacy, Tuanku Mizan Armed Forces Hospital, Kuala Lumpur, Malaysia

**Objectives:** This study aims to investigate the cost of healthcare clinical waste for six years from 2018 to 2023 in Tuanku Mizan Armed Forces Hospital (TMAFH). Precisely, we assess the relationship between continuous stakeholder training for clinical waste processes, which include generation, segregation, collection, storage, transportation, and treatment. We will then correlate how this impacts the hospital's healthcare clinical waste management cost.

**Methods:** We comprehensively analysed consignment notes generated by a service provider for TMAFH for six years from 2018 to 2023. Simultaneously, we collected data on the number of times the service provider conducted training for all the stakeholders.

**Results and Discussion:** For the year 2018, the cost was RM421,156.71; for 2019 was RM383,106.60; for 2020 was RM396,970.43; for 2021 was RM710,260.05, for 2022 was RM521,109.16 and for 2023 was RM602,203.77. Stakeholder training was done continuously ten, eight, six, four, six, and five times per year. Our results indicate a positive correlation between the cost of clinical waste and the number of times stakeholder training was conducted. Furthermore, the analysis revealed a significant association between the number of training times when increased reduced the service provider report on improper segregation by hospital staff. However, a further study examining all factors in depth to determine the causation is warranted.

**Conclusions:** This study provides valuable insights into the total cost of hospital waste management incurred by TMAFH for six years and how this may be affected by stakeholder training. The findings emphasise the need for interdisciplinary collaboration to develop a sustainable healthcare clinical waste management environment.

**Keywords**: cost, healthcare clinical waste, armed forces hospital

# Abstract: Formulation and Evaluation of Lupinifolin-loaded Microemulsion Gel for Acne Vulgaris

**Authors:** Wanwisa Khunawattanakul, Sakulrat Rattanakiat, Pawitra Pulbutr, Waraporn Saentaweesuk

**Affiliations:** 1 Integrative Pharmaceuticals and Innovation of Pharmaceutical Technology Research Unit, Faculty of Pharmacy, Mahasarakham University, Maha Sarakham, 44150, Thailand; 2 Pharmaceutical Chemistry and Natural Product Research Unit, Faculty of Pharmacy, Mahasarakham University, Maha Sarakham, 44150, Thailand

**Objectives:** This study investigates the antibacterial efficacy of lupinifolin, a prenylated flavanone extracted from *Derris reticulata* Craib. stems against acne-associated bacteria. Additionally, it focuses on developing a novel topical gel formulation by incorporating this highly lipophilic flavonoid into microemulsion (ME) systems.

**Methods:** The broth macro dilution method was employed to determine the minimum inhibitory concentration (MIC) and minimum bactericidal concentration (MBC) of lupinifolin against *Cutibacterium acnes* and *Staphylococcus aureus*. ME systems were developed using pseudoternary phase diagrams. The optimal ME system was then integrated into a gel using carbomer 940 and ammonium acryloyldimethyltaurate/carboxyethyl acrylate crosspolymer (ACA) as gelling agents. The resulting microemulsion gels were evaluated for appearance, pH, viscosity, and stability under cyclic temperature stress.

**Results and Discussion:** Lupinifolin demonstrated significant antibacterial activity against both *C. acnes* (MIC: 4 μg/mL; MBC: 32 μg/mL) and *S. aureus* (MIC: 8 μg/mL; MBC: 32 μg/mL). Glycerin-based MEs exhibited favourable properties, including appropriate pH, conductivity, zeta potential, and droplet size. Among the formulations, LME-G1A showed satisfactory clarity and ease of application. Both LME-G1A and LME-G1C demonstrated good stability following cyclic temperature stress.

**Conclusions:** This study reveals lupinifolin's substantial antibacterial activity against acne-causing bacteria, specifically C. acnes and S. aureus. Developing lupinifolin-loaded microemulsion gels, particularly LME-G1A, presents a promising innovative approach for acne treatment. Furthermore, this research advances the enhancement of water solubility, stability, and delivery of lupinifolin for topical application in managing acne.

**Keywords**: Microemulsion system, Lupinifolin, Acne vulgaris, *Cutibacterium acnes*, *Staphylococcus aureus*

# Abstract: Public Awareness and Knowledge of Dispensed Medicine Labelling Under the Poisons Regulations 1952 in the Federal Territory of Labuan

**Authors:** Siti Nadiah binti Dahlan, Ridhwan bin Abdullah, Heriman bin Mahali, Tan Zhi Shan Sujata, Jason Lee Kung Yin, Muhammad Naqeeb bin Ahmad Alhassora, Siti Nur Syathiroh binti Shahirul, Koay Chin Yin, Kamarudin Ahmad

**Affiliations:** 1 Federal Territory of Labuan Pharmacy Enforcement Branch; 2 Federal Territory of Labuan Pharmaceutical Services Department; 3 Miri Hospital, Sarawak

**Objectives:** This study aimed to assess the public awareness and knowledge of the Poisons Act 1952 and the Poisons Regulations 1952 concerning dispensed medicine labelling requirements in the Federal Territory (F.T.) of Labuan.

**Methods:** A cross-sectional study used a convenience sample of 405 Malaysian adults in F.T. Labuan. Online and hard copy questionnaires were administered to collect demographic data and participants’ awareness and knowledge of dispensed medicine labelling requirements. Data were analysed using descriptive statistics and chi-square tests. The questionnaire was previously validated and achieved a reliability score of 0.88.

**Results and Discussion:** 41.0% of the participants displayed good awareness of the Act and Regulations. 56.8% demonstrated good knowledge of the information required on medicine labels. Significant associations were found between knowledge scores and age groups (χ^2^ = 35.8, *p *= 0.003) and education levels (χ^2^ = 22.92, *p *= 0.028), suggesting these demographics influence knowledge levels. Other variables, such as gender, race, and occupation, showed no significant associations. However, 41.0% of participants were aware of the Act and Regulations, and 56.8% demonstrated good knowledge of the information required on the dispensed medicine labels, indicating room for improvement.

**Conclusions:** The high awareness and self-reported understanding of labelling requirements among the public in F.T. Labuan highlights the need for targeted educational programmes. These should improve the practical application of this knowledge to ensure safe medication practices.

**Keywords:** Medicine Labelling, Poisons Regulations, Public Awareness, F.T. Labuan, Knowledge Assessment

# Abstract: Strategic Financial Management in Health Emergencies: Policy Recommendations from Thailand's COVID-19 Experience

**Authors:** Suratchada Chanasophon, Wiraphol Phimarn, Kritsanee Saramunee, Pornchanok Srimongkol, Areerut Leelathanalerk, Panumart Pumart

**Affiliations:** 1 Social Pharmacy Research Unit, Faculty of Pharmacy, Mahasarakham University, TH; 2 Health Services and Pharmacy Practice Research and Innovation (HSPRI), Faculty of Pharmacy, Mahasarakham University, TH; 3 Faculty of Pharmaceutical Sciences, Khon Kaen University, TH

**Objectives:** The COVID-19 pandemic significantly impacted global healthcare systems, prompting service delivery and financial management adaptations. This study aims to synthesize policy recommendations for health financial management during emergencies, using Thailand's response to the COVID-19 pandemic as a case study.

**Methods:** This mixed-method qualitative study, conducted from August to December 2021, involved four key phases: (I) Documentary review of white papers from health policy organizations and national payers to identify emerging services and financial adjustments; (II) Semi-structured interviews with seventeen stakeholders, ranging from hospital directors to health insurance staff, capturing a spectrum of responses from regions with varying epidemic severity; (III) A policy drafting session with representatives from major national health agencies; (IV) Final Discussion with the Division of Health Economics and Health Security to refine policy recommendations. Data were transcribed and analyzed thematically.

**Results and Discussion:** The pandemic necessitated infection prevention measures such as social distancing, enhanced hygiene, and protective barriers. Telemedicine services gained acceptance, reducing face-to-face interactions. The ‘hospitel’ concept, merging hotel and hospital functionalities, provided isolation facilities, demonstrating cross-sector collaboration. Community pharmacies played a critical role in distributing medications and self-monitoring kits. Financially, the NHSO remained the primary payer, employing mechanisms like capitation, Diagnosis-Related Groups (DRG), fee schedule, and ceiling, adapted to increase reimbursement rates for COVID-19 services. Private hospitals assisted in receiving infected patients, and hospitals, community pharmacies, and delivery services were compensated—issues with delayed payments led to liquidity challenges for healthcare providers. Recommendations include flexible payment mechanisms, accelerated reimbursement processes, and an efficient information system to facilitate timely payments. Early provision of a lump-sum budget to healthcare providers is recommended to prevent financial instability during crises.

**Conclusions:** The COVID-19 pandemic has transformed Thai health services, introducing new norms and necessitating flexible financial mechanisms. These adaptations and the lessons learned should effectively inform future strategies for managing health finances during emergencies.

**Keywords**: health financial management, policy recommendations, emergency response, COVID-19, Thailand.

# Abstract: An Overview of Intravenous to Oral Antibiotics Conversion Practice in Multidisciplinary Wards Hospital Baling

**Authors:** Oi Aun Chyi, Afifah Zuki, Nur Syafiqah Abdul Salam

**Affiliations:** Department of Pharmacy, Hospital Baling, Kedah, Malaysia

**Objectives:** The objective of this study was to assess current practices of converting intravenous (IV) to oral (PO) antibiotics, the types of conversion therapy employed, and their influence on length of hospital stay (LOS).

**Methods:** A retrospective cross-sectional study was conducted on adult patients who received intravenous antibiotics between January and December 2021. Medical records of all patients meeting predetermined inclusion and exclusion criteria were reviewed.

**Results and Discussion:** Among the 455 patients studied, approximately 74.7% were eligible for early conversion, but only 48.5% underwent timely conversion. C-reactive protein levels, persistent fever, and unstable immune response were associated with lower conversion rates. Significant early conversion rates were observed for pneumonia (*P *< 0.001) and urinary tract infections (*P *= 0.002). Amoxycillin-clavulanate and Cefuroxime were the most commonly converted antibiotics (*P *< 0.001). Ceftriaxone is less frequently used in conversion therapy due to the lack of an oral formulation. Converted patients had a shorter duration of IV therapy (3.06 ± 0.77 days) and LOS (4.29 ± 1.64 days) compared to non-converted patients (6.11 ± 1.29 days, 7.63 ± 2.78 days, respectively; *P *< 0.001). However, nearly half of intravenous antibiotics were continued for five days without signs of infection before switching to oral antibiotics. Patients without COVID-19 were more likely to undergo conversion therapy (OR  =  3.37,95% Cl [2.00, 5.65]). Sequential therapy was the most commonly used method (72.9%). The study sheds light on antibiotic conversion challenges in a Malaysian district hospital lacking in-house physician services in such settings. The findings identify areas for improvement for both medical officers and clinical pharmacists.

**Conclusions:** Despite the demonstrated benefits of conversion therapy in facilitating earlier discharge, a significant number of eligible patients were not converted. Despite the importance of conversion practices in such scenarios, the low switch rate observed in pneumonia cases is particularly concerning. Of particular note, Ceftriaxone had the lowest conversion rate, highlighting a critical area for further investigation and improvement. These findings emphasize the importance of enhancing conversion practices as part of Antimicrobial Stewardship Program initiatives.

**Keywords**: antimicrobial stewardship program, antibiotic conversion, switch rate, intravenous to oral antibiotics, length of hospital stay

# Abstract: Chemical Pleurodesis with Povidone-Iodine 4% For Refractory Chylothorax In Neonates

**Authors:** Kae Shih Law, Lee Gaik Chan,

**Affiliations:** 1 Department of Pharmacy, Sarawak General Hospital; 2 Department of Paediatrics, Sarawak General Hospital

**Objectives:** Congenital chylothorax in neonates is a life-threatening disorder. It accumulates lymphatic fluids within the pleural cavity and results in respiratory distress, nutritional, metabolic and immunological complications with substantial morbidity and a high mortality rate of 50%. Conservative therapy consists of thoracostomy drainage, administering Octreotide with total parenteral nutrition and low-fat diet modification, which provide 50–100% chyle leak resolution. There is limited published literature on pleurodesis using Povidone iodine 4%, a sclerosing agent for neonates with congenital chylothorax who could not achieve total chyle leak resolution with conservative therapy. This article aims to report on two cases of pleurodesis using Povidone Iodine 4% for refractory congenital chylothorax who failed to respond to conservative therapy.

**Methods:** From January to December 2023, clinical data of two neonates who were treated for congenital chylothorax with Povidone Iodine 4% pleurodesis were retrospectively analysed, including clinical manifestations, pleural effusion investigations, treatment effects, outcome and complications.

**Results and Discussion:** Both patients have Down syndrome. One of them was hydrops fetalis, and the other one had developed worsening respiratory distress that required intubation at day 10 of life. Chest X-ray and Ultrasound detected pleural effusion in both cases; thus, diagnostic pleural tapping was done. Investigations of milky-coloured fluid collected were positively identified as chyle. However, despite thoracic drain in-situ, administered maximum dose of Octreotide intravenous infusion at 10 mcg/kg/hr with a mean duration of 17 days in combination with total parenteral nutrition, there was still high output chyle leak up to 75 mL/kg/day. Parents consented to the procedure. Both patients received 2 mL/kg of Povidone Iodine 4% pleurodesis once and found minimally low output chyle leak after the procedure and nil for the subsequent days. Drains were then removed. Patients remain symptom-free and have no recurrence throughout their stay in the ward. No side effects were observed after the procedure, and both patients were treated with a clinical cure.

**Conclusion:** Pleurodesis with povidone-iodine 4% is a cheap, effective and minimally invasive procedure with a high success rate in congenital chylothorax.

**Keywords**: Chylothorax, neonates, pleurodesis, povidone-iodine

# Abstract: Developing a Medication Value Assessment Tool: A Scoping Review of Perceived Value Measurement Instruments

**Authors:** Nur Ashikin Abdullah Sani, Ernieda Md Hatah, Chandini Menon A/P Premakumar

**Affiliations:** 1 Clinical Pharmacy Discipline, Faculty of Pharmacy, Universiti Kebangsaan Malaysia (UKM); 2 Department of Pharmacy, Kemayan Health Clinic, Bera District Health Office

**Objectives:** Understanding how patients perceive the value of their medications is crucial for improving patient satisfaction, adherence, and overall health outcomes. By gaining these insights, healthcare providers can better meet patient needs and expectations. Therefore, this study aims to provide a comprehensive overview of available perceived value scales, examining the relevant dimensions and domains that can be used to measure perceived value in medication.

**Method:** Using the scoping review methodology from the Joanna Briggs Institute Reviewers’ Manual 2015, we searched five electronic databases (Ovid, Web of Science, SCOPUS, CINAHL, and PubMed) from inception to September 30, 2023, for articles on validated tools or scales assessing perceived value in services, social experiences, brands, or products related to healthcare or in general. The articles identified were managed in EndNote and screened for inclusion and exclusion criteria by NAAS, with checks by EMH and CMAP. Full-text articles written in English on the scale's development, conceptualisation, and validation were included. Articles that focused on or related or adapted to health themes were prioritised. Articles were excluded if they were reviews, commentary, editorial articles, conference proceedings, or papers. Data on dimensions and psychometric properties of the tools were summarised in a table and discussed narratively.

**Results and Discussion:** The systematic search retrieved 5,887 articles. After removing duplicates and irrelevant titles/abstracts, 124 articles were included for full-text review, and 46 articles met the inclusion criteria for the final analysis. Common dimensions in assessing perceived value included Functional, Social, Acquisition, Emotional, Efficiency, Transaction, Aesthetic, Epistemic, Intentions, General, Economic, Utilitarian, Spatial, Image, Hedonic, Utility, Intrinsic, and Extrinsic. The identified dimensions provide a comprehensive framework for understanding the multifaceted nature of perceived value.

**Conclusions:** This study highlights the extensive range of validated perceived value scales encompassing various dimensions relevant to assessing individual perceptions in diverse contexts, including healthcare. Future research should focus on empirically testing these dimensions within the context of medication use, exploring their relevance and applicability in various patient populations and healthcare settings.

**Keywords**: “perceived”, “value”, “perceived value”, “health”, “scale”, “survey” and “questionnaire”

# Abstract: Potency of 70% Ethanol Extract of Bitter Melon Seeds (***Momordica charantia*** L.) as an Anti-inflammatory and Healing of Cut Wounds in Mice (***Mus musculus*** L.) DDY Strain

**Authors:** Norman Dyanto, Dony Hermanto, Nuzul Gyanata Adiwisastra, Diana Laila Ramatillah

**Affiliations:** 1 Faculty of Pharmacy, University of 17 Agustus 1945 Jakarta, Jakarta, Indonesia; 2 Program Study of Undergraduate Pharmacy, STIKes Prima Indonesia, Bekasi, Indonesia

**Objectives:** This research aims to know the potential of 70% ethanol extract of *Momordica charantia* L. seeds as an anti-inflammatory and healing cut wounds in *Mus musculus* L. DDY strain through histopathological observation of mice skin cells and measurement of cut length.

**Methods:** This type of research is an experimental in vivo study in mice. Fifty DDY strain mice were divided into five groups, namely the positive control group (povidone-iodine 10%), also known as K+; the negative control group (without treatment), also known as K-, first dose group (5%), also known as P1, second dose group (7.5%) also known as P2, and third dose group (10%) also known as P3. The back skin of mice was sliced with a scalpel parallel to the spine with a length of 3.0 cm. All groups received treatment by dripping twice daily from each Momordica charantia L. seeds extract test preparation. Ultimately, the skin samples were taken for a histopathological examination to see the number of inflamed cells.

**Results and Discussion:** The groups that decreased the number of inflammatory cells K+, P1, P2, and P3 were not significantly different (*p *> 0.05) but significantly different from K-. *Momordica charantia* L. seeds extract doses of 5%, 7.5%, and 10% showed overall healing of the cut.

**Conclusions:** This study concludes that 70% ethanol extract of *Momordica charantia* L. seeds effectively reduces the number of inflammatory cells in healing cut wounds.

**Keywords**: *Momordica charantia* L. Seeds, Inflammation, Cut Wounds

# Abstract: A Qualitative Exploration of the Challenges to Smoking Cessation among Patients with Chronic Obstructive Pulmonary Disease

**Authors:** Lukman Nul Hakim Md Khairi, Wan Aimuni Wan Zakaria, Mazlina Mukhtar, Muhammad Fais Sopi, Shubashini Gnanasan, Azlina Samsudin

**Affiliations:** 1 Pharmacy Department, Hospital Sultanah Nur Zahirah, Ministry of Health Malaysia; 2 Faculty of Pharmacy, Universiti Teknologi MARA (UiTM) Cawangan Selangor, Kampus Puncak Alam, Selangor, Malaysia; 3 Department of Respiratory Medicine, Hospital Sultanah Nur Zahirah, Ministry of Health Malaysia

**Objectives:** This study aimed to explore the challenges to smoking cessation among chronic obstructive pulmonary disease (COPD) patients treated at Hospital Sultanah Nur Zahirah (HSNZ), Kuala Terengganu, who continued smoking despite treatment.

**Methods:** We conducted semi-structured in-depth interviews with a purposive sample (*n* = 11) of patients recruited from the Respiratory Clinic registry of HSNZ. COPD patients who were unable to stop smoking were included. The interviews were audio-taped, transcribed verbatim, and thematically analyzed to identify key themes and subthemes.

**Results and Discussion:** A three-theme model emerged, illustrating patients’ decision-making processes. (1) Motivational factors: Participants’ attempts to stop smoking were hindered by multiple life-long habits that were difficult to break and low internal motivation; (2) Access to assisted smoking cessation: The smoking cessation program was poorly understood and not easily accessible, along with participants’ negative experiences with the pharmacotherapy; and (3) Requiring support while maintaining autonomy: Withdrawal symptoms and remarks made by family members and friends significantly influenced the participants’ emotions and perspectives on smoking cessation.

**Conclusions:** Understanding the challenges that COPD patients face in their attempts to stop smoking is vital for successful cessation. Three specific themes of barriers that hindered COPD patients from quitting were identified. Measures should be designed and implemented to ensure that COPD patients are internally motivated to quit and receive adequate support for smoking cessation. These findings also support a non-authoritarian approach to communication, which emphasizes understanding patients’ motivations and prior experiences, respecting their perspectives, and providing clear education and information.

**Keywords**: Chronic obstructive pulmonary disease, COPD smokers, smoking cessation, qualitative, Malaysia

# Abstract: Budget Impact Analysis of Introducing Ponatinib as Frontline Therapy for Philadelphia-positive Acute Lymphoblastic Leukaemia from the Perspective of Ministry of Health Malaysia

**Authors:** Muhammad Nasri Yusoff, Yi Jing Tan, Faridah Hanim Mustafa

**Affiliations:** 1 Department of Pharmacy, Hospital Tengku Ampuan Afzan; 2 Department of Pharmacy, Hospital Seri Manjung; 3 Department of Pharmacy, Hospital Sultan Haji Ahmad Shah

**Objectives:** This study aimed to determine the budget impact on the Ministry of Health Malaysia (MOH) of introducing ponatinib as frontline therapy for patients with Philadelphia-positive acute lymphoblastic leukaemia (Ph+ ALL).

**Methods:** A budget impact model was developed in Microsoft Excel to compare two scenarios: the current scenario where tyrosine kinase inhibitors (TKIs), imatinib or nilotinib, were used alongside induction chemotherapy, and the future scenario where ponatinib was introduced to partly substitute existing TKIs. The analysis was conducted over a 5-year time horizon without discounting. The target population comprised adult patients newly diagnosed with Ph+ ALL. Direct healthcare costs included drug costs, adverse event (AE) management costs, and reinduction chemotherapy costs for patients who did not achieve minimal residual disease negativity. The size of the eligible population, mortality rate, intervention mixes, share uptakes for each TKI, and costs were estimated using various local/regional sources, including official censuses and tariffs, published literature, and expert opinion. Efficacy and safety inputs were derived from the PhALLCON trial and drug monographs. All costs were reported in 2024 Malaysian Ringgit (RM). One-way sensitivity analyses were conducted to evaluate parameter and structural uncertainties.

**Results and Discussion:** It was projected that 28 new cases of Ph+ ALL among local adults would be diagnosed annually, contributing to subsequent cohorts. In the current scenario without ponatinib, treating newly diagnosed Ph+ ALL would cost RM2,312,119 in the first year, rising to RM7,570,669 by the fifth year. With ponatinib, costs were RM2,426,085 in the first year, reaching RM8,690,159 by the fifth year. Adopting ponatinib would, therefore, add RM113,966 in the first year to RM1,119,490 by the fifth year. Anticipated reductions in disease management costs would partially offset the increase in drug and AE costs. The 5-year incremental budget impact was RM2,954,377. Sensitivity analyses highlighted ponatinib's acquisition cost and Ph+ ALL prevalence as having the most significant impact on the budget.

**Conclusion:** Introducing ponatinib as frontline therapy for treating newly diagnosed Ph+ ALL cases would entail an incremental budget impact of RM2,954,377 over five years for the MOH.

**Keywords**: budget impact, ponatinib, tyrosine kinase inhibitor, Philadelphia-positive, acute lymphoblastic leukaemia

# Abstract: Trends of Importation of Medicines and Cosmetics via Courier in Johor, Malaysia pre-to-post Pandemic Coronavirus Disease 2019: Characteristics of Detained Parcels for Malaysian Checkpoint Border Agency to Ponder

**Authors:** Marina P., Thanusha A/P Mohan, Tuhfah Zahidah S., Muhammad Hakim J., Nurul Balqis Riza B., Nurfadhilah A.R., Fitriyah Amira A.H., Manesh Vari A/P Asogan

**Affiliations:** Pharmacy Enforcement Branch, Pharmaceutical Services Division, Johor State Health Department

**Objective**: To examine the characteristics of detained imported parcels containing medicines and cosmetics pre-to-post-pandemic COVID-19 via courier in Johor.

**Methods**: Cross-sectional analysis was conducted based on the data extracted from *Sistem Maklumat Kastam* (SMK). The data was collected retrospectively in three phases (pre-, during, and post-COVID-19) and analysed using Microsoft Excel.

**Results and Discussion**: 990 parcels containing 4,331 items were detained during the COVID-19 pandemic transition, with the majority being detained during the COVID-19 period (*n* = 607). Throughout this period, individual parcel detentions exceeded companies (*p *= 0.353). However, exporters from companies were significantly higher than individuals (*p *= 0.027). The top 5 exporting countries during the pre-to-post-pandemic COVID-19 transition were consistently the United States of America (*n* = 262), India (*n* = 174), Singapore (*n* = 146), and China (*n* = 63). The United Kingdom (*n* = 44) held the fourth position both before and during COVID-19 but was surpassed by South Korea (*n* = 22) in the post-COVID-19 period. Notably, iHerb, based in the United States of America (*n* = 134), emerged as the primary exporter during this period. During and after COVID-19, there has been an increase in detained parcels, medicines and cosmetics being the most commonly held items (*p *< 0.001). The most contravening laws observed were the importation of unregistered medicine (42.90%) and unnotified cosmetics (42.21%) (*p *< 0.001).

**Conclusions**: The COVID-19 pandemic, with the rise of online advertising, has transformed consumer habits in importing medicines and cosmetics. Looking ahead, the Malaysian Checkpoint Border Agency (MCBA), entrusted with supervising entry points in Malaysia, must give paramount importance to tackling the escalating challenge of importing medicines and cosmetics via parcels.

**Keywords**: medication, cosmetic, import, parcel, COVID-19, detention, exporters, customs

# Abstract: Characterisation of Unintentional Medication Discrepancies through Medication Reconciliation during Discharge: Preliminary Findings from General Medical Wards of a Tertiary Hospital

**Authors:** Phang Yen Yen, Theng Mei Ing, Oh Ai Ling, Ting Chuo Yew, Chong Wan Choon, Chieng Irene Yee Yew, Jong Alvin Chok Leong, Chiong Lily Hung, Tan Crystal Sing Yee, Teo Yi Jia, Dayang Nurfarahin Binti Abang Usop

**Affiliations:** 1 Pharmaceutical Services Division, Sarawak State Health Department; 2 Pharmacy Department, Sarawak General Hospital

**Objectives:** This study aimed to determine the prevalence and types of Unintentional Medication Discrepancies (UMDs) through medication reconciliation during hospital discharge.

**Methods:** A prospective observational study was conducted at the General Medical Wards of Sarawak General Hospital, a tertiary hospital in Sarawak, Malaysia, from May 2023 to Jun 2024. Adult patients aged 18 years and older, presenting with cardiovascular, endocrine, renal, and respiratory comorbidities, were selected using a convenient sampling method. The discharge medication reconciliation process by clinical pharmacists included comparing the list of medications in the discharge prescription with those documented in the medication history assessment form and inpatient medication charts. Discrepancies detected were verified with the prescribers to determine the nature of the discrepancies.

**Results and Discussion:** Out of 177 discharges, 48 (27.1%) were found to have at least one UMD. 61 UMDs were detected in 48 patients, making an average of 1.3 errors per patient. The most prevalent UMD was omission (54.1%), followed by inappropriate drug (18.0%) and discrepant dose (16.4%). These discrepancies were predominantly observed in the Alimentary Tract and Metabolism therapeutic class (36.1%) and Cardiovascular System therapeutic class (36.1%), followed by Blood and Blood Forming Organs therapeutic class (8.2%). The findings highlight the critical role of medication reconciliation in identifying and rectifying UMDs, thus enhancing patient safety during care transitions. The high prevalence of UMDs, particularly omissions, underscores the need for systematic interventions to improve discharge processes. Clinical pharmacists play an essential role in this context, ensuring that medication lists are accurate and complete, thereby preventing potential adverse drug events. Future initiatives should focus on integrating comprehensive medication reconciliation processes into standard discharge protocols. Training programs for healthcare professionals on the importance of accurate medication documentation and verification can further reduce the occurrence of UMDs.

**Conclusions:** This study has demonstrated the importance of medication reconciliation in safeguarding medication safety through detecting and rectifying UMDs during the transition of care by clinical pharmacists.

**Keywords**: unintentional medication discrepancy, prevalence, discharge prescription, medication reconciliation

# Abstract: Pre-dialytic Administration of Aminoglycosides (AMG) – A Single Center Study

**Authors:** Siti Latifah Azirah Mohd Noor, Amalia Athirah binti Ahmad Razak, Sharransusmita A/P Gunaraja, Nur Filzah binti Hazaha, Suzana Mustafa

**Affiliations:** Pharmacy Department, Raja Perempuan Zainab II, 15586 Kota Bharu, Kelantan, Malaysia

**Objectives:** Aminoglycoside is essential in treating severe infections caused by aerobic gram-negative bacteria. It is a concentration-dependent antibiotic and treatment success has been mainly associated with reaching a Cmax/MIC ≥ 8–10. Due to the Cmax/MIC-dependent mechanism, a pre-dialytic administration was suggested that would probably yield increased killing and reduced toxicity. Unfortunately, the suggested practice is not well-supported by clinical data. In HRPZ, the current practice is administering AMG at least 3 hours before dialysis. As such, we aim to report AMG pharmacokinetic observations in patients receiving renal replacement therapy (RRT) in HRPZ II.

**Method:** Data were retrospectively collected for patients identified through the TDM database, with serum aminoglycoside concentrations drawn either pre-dialysis, post-dose, or at random. Percentages dialysed were calculated with available serum concentrations pre- and post-dialysis. Demographic and clinical data were also collected from the patient's medical records. The data were gathered and analysed using Statistical Package for the Social Sciences (SPSS) software version 20.0 for Windows.

**Results and discussion:** Thirty-six RRT patients received AMG treatment for 2022–2023. However, only 26 patients with 47 blood samples were included in the analysis. They were primarily treated in critical care wards, mostly with Amikacin (*n*  =  17). The mean (SD) first doses of AMG were 10.9 (3.8) mg/kg and 2.5 (0.81) mg/kg for Amikacin and Gentamicin, respectively. Fifty-eight per cent of the patients received an inadequate dosage of AMG, which significantly contributed to the subtherapeutic concentrations of peak AMG in 62% of the patients (*p* < 0.05). No significant difference was observed in higher trough concentrations with the adequate AMG dosage. AMG was highly dialysable, with the mean (SD) percentage of dialysable being 48% (11.8%) and 54% (14.5%) for Amikacin and gentamicin, respectively.

**Conclusions :** Pre-dialytic administration of a higher first dose of AMG allows for achieving the PKPD target of Cmax/MIC as early as possible. However, therapeutic drug monitoring should be employed to design precise doses for optimum bacterial killing and toxicity prevention.

**Keywords:** Aminoglycosides, dialysis, dialysable

# Abstract: Mortality Rate and Therapeutic Outcome Among Diabetic Patients With Asthma Complication Who Have Been Vaccinated With COVID-19 at Hospital Educational Jakarta

**Authors:** Maria Sheila Setya Ningtyas, Diana Laila Ramatillah, Muhammad Junaid Farrukh

**Affiliations:** 1 Department of Pharmacy, Universitas 17 Agustus 1945 Jakarta, Jakarta, Indonesia; 2 Department of Clinical Pharmacy, UCSI University, Kuala Lumpur, Malaysia

**Objectives:** This study aims to determine the mortality rate and therapeutic outcomes of diabetes patients with asthma complications who have been vaccinated with COVID-19.

**Methods:** This study employed a retrospective and prospective cohort method at Jakarta Educational Hospital for three months. The retrospective period covers January 2022 to December 2023, while the prospective period extends from December 2023 to February 2024. Purposive sampling was used to select samples that meet the inclusion criteria. The data collected includes the patient's sociodemographics, laboratory results, therapy used, and the therapeutic and clinical outcomes. The data was processed using IBM version 26 and displayed in graphic and table form.

**Results and Discussion:** Based on the research carried out, 150 samples were obtained. Among 150 patients, 76% were women, with most ages > 50 years (80.7%). The type of Covid-19 vaccine that most patients received was Sinovac (48.6%). There was no significant relationship between the therapy used and the mortality rate. The outcome therapy was asthma attacks two times/week (65.3%) and HbA1c value for the poor control group (48%), where patients with poor HbA1c control have an average survival of around 85% when the patient has been diagnosed after 15 years.

**Conclusions:** It can be concluded that the average patient mortality is 3.33% with the results of HbA1c therapy, namely poor control of 85%. There is no significant relationship between the therapy patients use and clinical outcomes.

**Keywords**: Mortality, Outcome Therapy, Diabetes, Asthma, Covid-19 Vaccination

# Abstract: The Incidence, Clinical Outcomes, and Cost Implications of Early Intravenous-To- Oral Antibiotic Switching for Lower Respiratory Tract Infections in the Medical Wards of a Major Hospital In Sarawak

**Authors:** Sim Shyang Jiun, Chan Pui Sheon, Cindy Lau Sing Ling, Chan Ngiik Ching, Andy Ong Kang Thy, Esteban Chin Yu Hern

**Affiliations:** 1 Pharmacy Department, Bintulu Hospital, Sarawak; 2 Medical Department, Bintulu Hospital

**Objectives:** This study aimed to evaluate the use of switch therapy in hospitalized lower respiratory tract infection (LRTI) patients and to assess the clinical and economic impact of shortened intravenous (IV) antibiotic courses.

**Methods:** We conducted a 3-month prospective cohort study of adults admitted with LRTI and initially treated with IV antibiotics. Switching was defined as transitioning from IV to oral antibiotics without interrupting therapy. Patients were categorized into timely switch and delayed/not switched groups. Patient demographics were analyzed using descriptive statistics. Non-parametric tests and independent t-tests were used to examine the relationship between group assignment and clinical outcomes. Factors leading to late switching were identified.

**Results and discussions:** Among 81 LRTI patients, 89% had community-acquired pneumonia (CAP). Approximately 75% transitioned promptly to oral antibiotics, while others continued IV treatment beyond day 3. Converted patients had significantly shorter IV therapy durations (*P *= 0.016) and hospital stays (*P *< 0.001). Injection Amoxicillin/Clavulanate (94%) was used empirically, with only 5% showing positive cultures. Delays in culture results and completing IV antibiotics were common barriers to timely switching, causing an average delay of 2.2 days and RM 580.82 in additional costs—no patients required readmission or restarted IV antibiotics within five days in either group.

**Conclusions:** Early switching led to shorter hospital stays and fewer antibiotic days without worsening outcomes. Prompt culture and sensitivity could minimize unnecessary antibiotic use. Improved LRTI diagnosis distinguishes viral infections, avoids unnecessary IV antibiotics, refines treatment, benefits patients, and reduces healthcare costs.

**Keywords:** intravenous to oral antibiotic switch, lower respiratory tract infections

# Abstract: Evaluating The Belt Conveyor Utilization Policy On Waiting Time And Productivity In Outpatient Pharmacies

**Authors:** Nurhairani Abdul Latif, Saidatul Sheeda Ahmad Shukri, Muhamad Fuad Ali, Siti Nur Su’aidah Nasarudin, Hajar Atiqah Abdul Kadir, Sharifah Shafawati Syed Mohd Hamdan

**Affiliations:** 1 Pharmacy Policy and Strategic Planning Division, Ministry of Health, Malaysia; 2 Planning Division, Ministry of Health, Malaysia

**Objective:** This study aims to evaluate the waiting time and productivity of the outpatient pharmacy in facilities with and without belt conveyors.

**Method:** A cross-sectional study evaluated waiting time and productivity in 17 facilities with belt conveyors at the outpatient pharmacy. The control group, facilities without belt conveyors, were identified using the propensity score matching (PSM). The propensity score was computed based on variables such as the facility's category, the total number of counters, the type of OPD counter, the range of prescriptions dispensed, the total number of pharmacy staff, and the space area. The 1:1 matching without replacement technique was used based on the closest propensity score. The waiting time and productivity data for both groups were then analysed using an independent t-test for productivity and Mann-Whitney for waiting time. Significance was defined as *p*<0.05, ensuring the validity and reliability of the results.

**Result and Discussion:** The results indicated no statistically significant difference in waiting time for facilities with and without belt conveyors (U = 136.00 OR z = −0.327, *p *= 0.744). In addition, productivity showed no significant difference between the facilities with belt conveyors (M = 3.60, SD = 1.192) and without belt conveyor facilities (M = 3.64, SD = 1.273); t(32)  = −0.109, *p *= 0.914. The findings indicate that introducing belt conveyors does not enhance waiting times or productivity within the outpatient pharmacy.

**Conclusion:** The study provides valuable insights to policymakers on the belt conveyor utilisation policy in outpatient pharmacies, thus indicating a need to revise the current policy to align with the practice and carefully consider all factors before introducing innovations in pharmacy layouts.

**Keywords:** belt conveyor, waiting time, productivity, outpatient pharmacy

# Abstract: The Effectiveness Evaluation of The Pfizer Booster Vaccine In Indonesia And Malaysia

**Authors:** Raihan Fadhil Muhammad, Diana Laila Ramatillah, Hasniza Zaman Huri

**Affiliations:** 1 Pharmacy Faculty, Universitаs 17 Аgustus 1945 Jаkаrtа, Indonesia; 2 Faculty of Pharmacy, Malaya University

**Objectives:** This study aims to evaluate the effectiveness of the Pfizer booster vaccine on Malaysian and Indonesian participants who are against COVID-19.

**Methods:** This Cross-sectional study included only Pfizer booster vaccine participants from Indonesia and Malaysia. The sampling was done using convenience sampling with all participants who met the inclusion criteria enrolled in this research. The collected data from 402 respondents (202 Indonesian and 200 Malaysian) show there is a significant correlation between Sociodemographic and Nationality (*P *< 0.001).

**Results and Discussion:** Most Indonesians received the Sinovac Biotech Vaccine as their first and second dose, while Malaysians received Pfizer as their first and second. Muscle pain, headache, dizziness, drowsiness, shivering, hunger and thirst and nausea or vomiting are the commonly experienced side effects of the Pfizer booster vaccine. The COVID-19 history with Nationality shows a significant correlation (*P *< 0.001), with most participants never getting infected with COVID-19 until now or ever being infected before the booster vaccine. The data also revealed that the Pfizer booster vaccine shows a beneficial adverse drug reaction in both countries as it lessens the allergies of 9.7% of Indonesians and 1% of Malaysians.

**Conclusions:** The Pfizer booster vaccine is very effective in both countries as it protects the participants from getting infected by COVID-19, and there were no dangerous results from using the Pfizer booster vaccine.

**Keywords**: Booster, Covid-19, Effectiveness; Pfizer, Vaccine

# Abstract: Association Of Sociodemographic And Comorbidities With Clinical Outcomes in Diabetes and Diabetes Hypertensive Patients Vaccinated With Sinovac Vaccine

**Authors:** Sofia Ngarso, Diana Laila Ramatillah, Hasniza Zaman Huri

**Affiliations:** 1 Magister Ilmu Farmasi, Pharmacy Faculty, Universitas 17 Agustus 1945, Jakarta; 2 Pharmacy Faculty, Malaya University, Malaysia

**Objectives:** This study aims to evaluate the clinical outcomes of diabetic and diabetic patients with hypertension who have been vaccinated with the Sinovac vaccine.

**Methods:** This study used a prospective and retrospective cohort analytical observational design. The data was collected from a private hospital in South Tangerang between April 2021 and May 2024. The study population consisted of patients diagnosed with diabetes and hypertension who had been vaccinated with the Sinovac vaccine. The sampling technique used convenience sampling.

**Results and Discussion:** Based On the 300 patients, 56.3% were female patients, with the majority of the ages coming from the 41 to 60 years old group (46.7%) and 47.6% from the >60 years age group. There was no significant association between the length of diagnosis of diabetic patients and diabetes with hypertension and clinical outcomes. There was an association between kidney failure and clinical outcomes, with the death of 8 people (2.7%) in patients with stage 4 and 5 renal failure. There was no association between socio-demographics and clinical outcomes. In comorbidities with Diabetes Mellitus, 3.33% of patients die. The patients diagnosed with the disease >10 years have a higher percentage of mortality compared to patients diagnosed <10 years.

**Conclusions:** It can be concluded that, on average, diabetic patients and diabetic patients with hypertension who have received the Sinovac vaccine can survive as much as 94.3% and those who die 5.7%. There is a correlation between kidney failure and comorbidity with clinical outcomes.

**Keywords**: Clinical outcome, diabetes, hypertension, Sinovac, Sociodemography.

# Abstract: Medication Adherence in Type II Diabetes Mellitus Patients

**Authors:** Nurjianti Ipaenim, Maryanti Tuharea, Diana Laila Ramatillah

**Affiliations:** Department Faculty, Universitas 17 Agustus 1945 Jakarta, Jakarta, Indonesia

**Objective:** This research is a systematic literature review that aims to examine the pattern of medication adherence in type II diabetes mellitus patients, the factors that influence this adherence, and the strategies needed to improve medication adherence.

**Methods:** This research was conducted following the PRISMA statement. The inclusion criteria used were studies on treatment adherence for type II diabetes mellitus, studies published in English, studies published within the last five years (2019–2024), and studies published in journals.

**Results and Discussion:** After a selection process, 18 research journals were obtained and analyzed. The results of the analysis showed a high prevalence of non-adherence in the use of antidiabetic drugs, with the main reasons being forgetting, financial limitations, and missing symptoms. The complexity of the treatment regimen is associated with lower levels of treatment compliance and leads to poor glycemic control. In addition, it was found that female patients had better adherence rates than males, and the highest adherence rates were found in repaglinide users, followed by pioglitazone and sitagliptin users. However, no relationship was found between glycemic control and adherence to antidiabetic drug use.

**Conclusions:** This study concludes that interventions to overcome polypharmacy, patient education, support, and intensive monitoring can increase medication adherence in type II diabetes mellitus patients. It is hoped that future research can be carried out to implement and intervene in appropriate education and counselling models to improve treatment compliance in patients with type II diabetes mellitus.

**Keywords:** medication, adherence, diabetes mellitus type II.

# Abstract: Nirmatrelvir-ritonavir Treatment and Risk of Long COVID Symptoms: A Cross-Sectional Study at a Primary Health Clinic Malaysia

**Authors:** Tai Chia Woon, Juwita Asikhin Binti Juma’at, Anu Priya A/P Mukunan, Nithya Devi A/P Devi, Sharon Augustine Amburose, Lim Shu Min, Lim Chin Wen, Noraian Idris, Farah Nabihah Johari, Nur Afiqah Ithnin, Nur Syafiqah Binti Mohd Asri, Siti Aishah Binti Khairul Hisham, Muhammad Danial Bin Zahid, Chong Hui Jing, Muhammad Asyraff Bin Zarwi

**Affiliations:** Pharmacy Department, Mahmoodiah Health Clinic Johor Bahru (KPLJB), Malaysia

**Introduction**: Nirmatrelvir-ritonavir is an oral antiviral developed to treat people with mild-to-moderate coronavirus 2019 (COVID-19). However, the effect of the treatment on Long COVID symptoms has been less clear.

**Objectives**: To determine the proportion of patients who experienced Long COVID and associated factors among COVID-19 patients who completed the oral nirmatrelvir-ritonavir 5-day treatment.

**Methods**: This cross-sectional study involved all COVID-19 patients treated with nirmatrelvir-ritonavir from May to December 2022 at KPLJB. All eligible patients were contacted by phone in March 2023 to interview on their medication adherence and experience of any Long COVID symptoms after completing the treatment.

**Results and Discussion** We collected 415 patients prescribed with nirmatrelvir-ritonavir during the study period. Around two-thirds of the patients (63.4%) have one or more comorbidities while 82%had their COVID-19 vaccine booster dose. Among all the 223 patients who were contactable by phone, 16 patients (7.2%) were not compliant with nirmatrelvir-ritonavir, mainly due to side effects. After completing the 5-day nirmatrelvir-ritonavir treatment for over three months, 98 patients (43.9%) experienced at least one Long COVID symptom. One-fifth (21.5%) of them still experienced chronic fatigue, persistent cough (17.9%) and insomnia (9.4%). After adjusting for gender, smoking, history of medication allergy and comorbidities (obesity and T2DM) through logistic regression analysis, our studied subjects who had an additional COVID-19 vaccine booster dose had 58% lower odds of getting Long COVID symptoms.

**Conclusion**: Treatment with nirmatrelvir-ritonavir among non-hospitalized individuals vaccinated with a booster dose was associated with lower odds of Long COVID symptoms.

**Keywords:** nirmatrelvir-ritonavir, Long COVID, COVID-19, medication adherence, vaccine

# Abstract: Prevalence and characteristic of drug-related problems (DRPs) amongst patients attending pharmacist-led Haemodialysis Medication Therapy Adherence Clinic (HD-MTAC): A longitudinal study

**Authors:** Shan Lii Ching, Ernieda Hatah, Farida Hanim Islahudin, Nurul Ain Mohd Tahir, Hui Yin Tay

**Affiliations:** 1 Department of Pharmacy, Universiti Kebangsaan Malaysia, Kuala Lumpur, Malaysia; 2 Department of Pharmacy, Hospital Tuanku Ampuan Rahimah, Klang, Malaysia

**Objectives:** This study aimed to investigate the DRP prevalence and characteristics in HD patients undergoing pharmacist-led HD-MTAC over time.

**Methods:** A retrospective observational analysis was conducted on HD-MTAC patient records at a tertiary hospital in Malaysia. The inclusion and exclusion criteria included patients’ records of chronic HD patients aged 18 to 75 years, with up to a maximum of 20% incomplete data and were actively seen in HD-MTAC between 2021 and 2023. Patients’ demographics, DRP prevalence, and characteristics were reviewed and recorded. The DRPs were classified according to the Pharmaceutical Care Network Europe Association (PCNE), version 9.0. The prevalence and characteristics of DRPs were descriptively analysed across visits.

**Results and Discussion:** This study included 76 patients’ records and involved 569 visits. The review covered up to eleven visits over 20 months. The mean dialysis duration was 6.36 ± 5.78 years, with an average of 7.88 ± 1.49 co-morbidities per patient. Patients were prescribed an average of 7.91 ± 2.80 medications and a daily pill burden of 17.44 ± 7.63. A total of 585 DRPs were identified and intervened, with almost all patients (*n* = 73, 96.1%) experiencing at least one DRP during their visits. Longitudinal analysis showed the mean DRP peaking at visit four (1.23 ± 1.28), which decreased to its lowest point at visit seven (0.72 ± 0.93). The trend subsequently increased again until visit eleven (1.11 ± 1.32). Overall characteristics of the identified DRPs showed the majority falling into the sub-domain category of non-optimal drug effectiveness (*n* = 285, 79.6%) followed by unnecessary drug treatment (*n* = 148, 90.8%). Individual visits displayed similar characteristics, except for visit nine, which showed an inversed pattern with DRPs predominating in the unnecessary drug treatment sub-domain followed by non-optimal drug effectiveness.

**Conclusions:** This study reveals that DRPs among HD patients occur over time, peaking at a particular visit, with new drug-related issues emerging later in the disease. Further research is needed to investigate these patterns to understand the longitudinal trends in DRP prevalence and characteristics, highlighting critical areas and time points for HD-MTAC improvements.

**Keywords**: MTAC, haemodialysis, DRP, medication management

# Abstract: Outcome Treatment of Cardiovascular Disease Among AstraZeneca Vaccinated Patients in Jakarta

**Authors:** Phoebe Clarissa Chastity, Diana Laila Ramatillah

**Affiliations:** Department of Pharmacy, Universitas 17 Agustus 1945 Jakarta, Jakarta, Indonesia

**Objectives:** The objective of this research is to evaluate the changes in cardiovascular disease before and after vaccination and determine whether AstraZeneca impacts cardiovascular disease outcomes.

**Methods:** This research employs an analytical survey with a cohort approach conducted prospectively and retrospectively, with data collection in the Jakarta area. Simultaneously, we collected data on cardiovascular treatment outcomes and side effects, age, disease complications, and AstraZeneca vaccination. Data was gathered using validated questionnaires that were tested for reliability. Statistical analysis, including crosstab analysis, will be conducted to determine correlations with a specified significance level.

**Results and Discussion:** The study focuses on comparing the changes in patients’ CVD-related comorbidities before and after vaccination among 200 participants, with the majority female 115 (57.5%) and male 85 (42.5%) with a grouping of participants with age <30 (15.5%), age <45 (39.5%), and age >45 (45.0%). Substantial correlation was found between several characteristics, including comorbidities, sociodemographic variables, patients’ medication usage before and after vaccination, and the association between vaccination and comorbid illnesses (*P* < 0.05). The data reveal a strong connection between vaccination and a history of cardiovascular disease in individuals. This association emphasizes the vaccine's potential influence on CVD-related symptoms following inoculation. Examining patients’ medication usage before and after vaccination reveals a substantial relationship between vaccination and the prevalence of comorbid conditions, showing an essential connection between a patient's cardiovascular disease history and vaccination history.

**Conclusions:** This study offers valuable insights into the influence of the AstraZeneca vaccine on CVD-related symptoms post-vaccination in Jakarta. The findings highlight that the vaccine significantly impacts the risk of cardiovascular disease, which is crucial for understanding its effect on the management and progression of cardiovascular health.

**Keywords**: AstraZeneca vaccine, cardiovascular, comorbidities, treatment outcomes

# Abstract: Characteristics and Management of Patients with Newly Diagnosed Type 2 Diabetes Mellitus at Primary Healthcare Setting: Role of Pharmacist in a Multidisciplinary Health Care Team

**Authors:** Tai Chia Woon, Dheviya A/P Rajendran, Farah Nabihah Binti Johari, Foo Jing Chuan, Hazlina Binti Iurat, Justine See Kai Ting, Lai Siew Yen, Lim Chin Wen, Lim Shu Min, Muhammad Zuyyin Bin Wafar, Mustaqim Bin Abd Rashid, Ngo Swee San, Nurul Najihah Binti Azni, Nur Raihanah Binti A. Rahman, Siti Aiyshah Binti Shahid, Siti Khadijah Binti Zamri, Tan Mei Fang, Yasmin Nabilah Binti Zakaria, Yasodha A/P Govindasamy

**Affiliations:** Pharmacy Department, Johor Bahru District Health Office, Ministry of Health Malaysia

**Introduction**: In patients newly diagnosed with type 2 diabetes mellitus (T2DM), the target of HbA1c < 6.5% should be achieved within six months due to the legacy effects. Early use of combination therapy accompanied by lifestyle modification to achieve the target is strongly recommended.

**Objectives**: To determine the demographic and clinical characteristics of patients newly diagnosed with T2DM at all the 14 health clinics under Johor Bahru District Health (PKDJB) and to discuss the role of pharmacists in a diabetes multidisciplinary team (MDT) in primary healthcare settings.

**Methods**: This cross-sectional retrospective, multi-centre study involved all newly diagnosed T2DM patients in PKDJB from 1 February 2022 to 31 January 2023. All eligible patients whom pharmacists counselled were recruited. The patient's demographic characteristics and clinical data (HbA1c and body mass index, BMI, presence of comorbidities, chronic complications, and prescribed pharmacotherapy) were included in the counselling form. All these data were retrospectively reviewed and analyzed.

**Results and Discussion**: A total of 1437 patients were recruited. Most patients were between 55 and 59 years old (16.28%), and the mean age at diagnosis was 53.68 ± 13.02 (Mean ± SD) years old. Most patients were female (54.8%) and Malay ethnicity (56.4%). The mean HbA1c during diagnosis was 8.30 ± 2.19%. The prevalence of hypertension was 62.7%, dyslipidemia was 64.79%, and obesity was 77.73% (mean BMI 28.32 ± 5.39 kg/m^2^). As for complications, 8.07% of patients have diabetic kidney disease, followed by diabetic neuropathy (2.16%). Metformin monotherapy (85.52%) was the most used therapy, followed by metformin and gliclazide dual therapy (8.49%). Among those with HbA1c>10%, younger age was noted (47.88 ± 11.75 years old), and only 10.21% of them were treated with insulin as suggested by the CPG Malaysia. As an MDT member in diabetes care, pharmacists can help optimize the treatment in a given period, ensuring medication knowledge and adherence, which results in glucose attainment and prevents chronic complications.

**Conclusion**: Suboptimal diabetes treatment is standard in newly diagnosed T2DM patients at PKDJB, especially those with higher HbA1c. Pharmacists are crucial in optimizing the treatment regimen and preventing chronic complications.

**Keywords**: newly diagnosed, type 2 diabetes, pharmacists, primary healthcare, multidisciplinary team

# Abstract: Side Effects of Metformin in Type 2 Diabetes Mellitus: A Systematic Review

**Authors:** Maiyeni Sabrina, Diana Laila Ramatillah

**Affiliations:** Pharmacy Faculty, Universitas 17 Agustus 1945 Jakarta

**Objectives:** This study aims to evaluate the side effects of metformin in type 2 diabetes Mellitus.

**Methods:** This systematic review was conducted according to the Preferred Reporting Items for Systematic Reviews and Meta-Analyses (PRISMA) guidelines. Articles in English were searched in Medline (via PubMed) from 2019 to 2024. Search terms related to the side effects of metformin were combined with terms for type 2 diabetes mellitus. The initial search identified 3,861 articles. Based on text availability, 3,577 full-text articles were reviewed. After excluding 2,636 articles based on article type (clinical and randomized controlled trials) and publication date, 200 articles remained. Abstract screening reduced this to 13 articles, and full-text screening further reduced this to three.

**Results and Discussion:** Three eligible studies were identified that provided quantitative and qualitative data on the side effects of metformin in type 2 diabetes mellitus. The studies revealed that 40 patients (53.3%) experienced gastrointestinal (GI) effects, including epigastric and abdominal pain, nausea, vomiting, diarrhoea, and bloating, with an average metformin use duration of 7.8 years. Additionally, metformin therapy was associated with reduced testosterone levels in males with T2DM who had normalized blood glucose control, indicating a potential link between metformin use and the high prevalence of low testosterone levels in these patients. There was also a significant increase in the likelihood of vitamin B12 deficiency with higher doses of metformin, with those taking more than 2000 mg daily having nearly 40 times the odds of deficiency compared to non-users.

**Conclusions:** The collected data indicates that metformin use in type 2 diabetes mellitus is associated with notable side effects, including gastrointestinal disturbances, decreased testosterone levels, and vitamin B12 deficiency.

**Keywords:** Side effects, metformin, type 2 diabetes mellitus

# Abstract: Analysis of Saponins in Several Plants Using the HPLC Method and Their Use in Pharmaceutical Products

**Authors:** Vony Intan Prasasty, Silvia Rizky

**Affiliations:** Pharmacy Faculty, Universitas 17 Agustus 1945 Jakarta

**Objectives:** This research aims to determine the content of saponin compounds found in several types of plants and their health benefits.

**Methods:** This research uses computer equipment and the Google Scholar database, Research Gate, NJC journal, and Science Direct. The type of research used is an article review using data sources directly collected by the researcher. The data search was done through a Google Scholar database, using the keywords “saponin levels and HPLC”. The screening results that met the requirements were used as references for 25 articles.

**Results and Discussion:** The studies utilized dried leaf samples extracted using ultrasonification and centrifugation to obtain a clear solution. The saponins were then analyzed using HPLC with various columns, resulting in a retention time of 53.09 minutes and an average saponin content of 1.05%. Saponins are significant because they enhance immunity, exhibit antitumor, anti-inflammatory, antifungal, and antiviral properties, reduce blood glucose and lipid levels, diminish oxidative stress, and improve cardiovascular function.

**Conclusions:** The search results of several researchers above show that saponin analysis in several plants can be carried out using HPLC with a wavelength of 205 nm. Saponin compounds as secondary metabolites have many benefits in the pharmaceutical field, including anti-inflammatory, hepatoprotective activity, immunological improvement, antitumor, anti-ageing, treatment of cerebral oedema, lung injury, myocardial ischemia, reperfusion injury and gastric ulcers.

**Keywords**: HPLC, Pharmacology, Saponin, review article

# Abstract: Evaluating Adequate Use of Acid-Suppressive Therapy for Stress Ulcer Prophylaxis (SUP) in Paediatric Intensive Care Unit (PICU)

**Authors:** Hiba Arif, Muhammad Gulzaib, Waseem Ullah Khan, Márió Gajdács, Long Chiau Ming, Suresh Shanmugham, Shazia Jamshed

**Affiliations:** 1 Advanced International Hospital, Islamabad, Pakistan; 2 Shifa International Hospital, Islamabad, Pakistan; 3 Shifa College of Pharmaceutical Sciences, Shifa University, Islamabad, Pakistan; 4 Department of Pharmacy, Boitekanelo College, Gaborone, Botswana, Southern Africa; 5 Department of Oral Biology and Experimental Dental Research, Faculty of Dentistry, University of Szeged, Hungary; 6 School of Medical and Life Sciences, Sunway University, Kuala Lumpur, Malaysia; 7 Pharmacy Practice, School of Pharmacy, IMU, Kuala Lumpur, Malaysia; 8 Jinnah Women University, Karachi, Pakistan

**Objectives**: This study evaluated the adequate use of acid-suppressive therapy for stress ulcer prophylaxis (SUP) in a paediatric intensive care unit in a quaternary care setting.

**Methods**: It is a retrospective, descriptive, single-centre study performed in a paediatric intensive care unit at Shifa International Hospital, Islamabad. Electronic medical records of 588 critically ill children were evaluated for adequacy of stress ulcer prophylaxis. All paediatric patients admitted to the critical care wards of the hospital from January 2020 to December 2022 were included. Exclusion criteria included all patients admitted to PICU with gastrointestinal bleeding on admission, patients on prior acid suppressive therapy, and oncology paediatric patients.

**Results and Discussion**: A total of 588 patients fulfilled the criteria included in the current research. Among the Proton Pump Inhibitors (PPI) and histamine h2-receptor antagonists (H2 blockers), omeprazole was the most frequently prescribed in PICU. Esomeprazole was prescribed in older children, while famotidine was prescribed the least. Most frequently prescribed children were presented with respiratory and neurological conditions. Slightly more than 60% were prescribed an acid-suppressive drug during their PICU stay as they concomitantly received antibiotics, systemic corticosteroids, analgesics, anticoagulants, antiepileptics, and sedatives. Dose optimization was identified as a concern as around 30% of total SUP prescriptions were identified as inappropriate (requiring paediatric dose adjustments) or having medicines with prominent drug-drug interaction potential with PPI. Mechanical ventilation was seen in 59% of the patients, which seems to be a prominent factor in prescribing SUP. No case of gastrointestinal bleeding was observed. Nearly 40% were prescribed an acid-suppressing drug at the time of discharge.

**Conclusions:** SUP prescribing in the PICU setting is common. The use of omeprazole was predominant. The highest number of SUP prescriptions were seen in patients with mechanical ventilation and prescriptions of NSAIDS and systemic corticosteroids.

**Keywords**: acid-suppressive therapy, stress ulcer prophylaxis, proton pump inhibitors

# Abstract: Effectiveness of Posaconazole Salvage Therapy for Mucormycosis in Hematology Patients

**Authors:** Yasir habib, Zeeshan Ahmad Khattak, Muhammad Ali Mehdi, Samia Afaq, Aun Bin Azhar, Long Chiau Ming, Suresh Shanmugham, Shazia Jamshed

**Affiliations:** 1 ISMA Institute of Pharmaceutical Sciences, Gujrat, Pakistan; 2 Burjeel Medical City, Abu Dhabi, United Arab Emirates, UAE; 3 Faculty of Pharmacy, The University of Lahore, Punjab, Pakistan.; 4 Azhar Hospital Gujar Khan, Rawalpindi; 5 Pharmacy Practice, School of Pharmacy, Sunway University, Kuala Lumpur, Malaysia; 6 Pharmacy Practice, School of Pharmacy, IMU, Bukit Jalil Campus, Kuala Lumpur, Malaysia; 7 Shifa College of Pharmaceutical Sciences, Shifa University, Islamabad, Pakistan

**Objectives:** Mucormycosis, a life-threatening invasive fungal infection caused by moulds of the order Mucorales, predominantly affects immune-compromised and diabetic patients. Standard management includes a three-pronged strategy, including initiating initial therapy, eliminating or controlling underlying medical conditions, and performing debridement of affected tissues. Amphotericin B is standard initial therapy; however, many patients are refractory or intolerant, necessitating alternative salvage therapies like posaconazole. This study explores individual Mucormycosis cases, focusing on underlying medical conditions, causative agents, infection sites, debridement, and reasons for the failure of initial standard therapy (intolerance, refractory response, or both).

**Methods:** A retrospective single-centre study was conducted on the efficacy of posaconazole salvage therapy in mucormycosis patients who are intolerant or refractory to initial amphotericin B therapy. Data was collected from the oncology and haematology departments of a quaternary care hospital in Pakistan, encompassing twelve patients. Information recorded included causative agents, infection sites, initial standard therapy, reasons for discontinuation, debridement, dose and duration of posaconazole, patient response at follow-ups, and Posaconazole-associated adverse drug events.

**Results and Discussion:** All patients had confirmed invasive mucormycosis, with infection sites including sinonasal (*n* = 7), pulmonary (*n* = 3), rhino-orbital (*n* = 1), and nasal (*n* = 1). Causative agents identified were Mucor (*n* = 4), Rhizomucor (*n* = 3), and Rhizopus (*n* = 5). Six patients were refractory, four were intolerant, and two were both to amphotericin B therapy. Posaconazole was administered as 300 mg delayed-release tablets twice daily on day one, followed by 300 mg once daily from day two. Mild to moderate adverse events (*n* = 9) were reported, but none advised or discontinued therapy. Stable disease was observed in nine patients, and partial response was observed in three patients after a fortnight of posaconazole use. At the end of therapy or the last follow-up, the success rate was 80%, with eight out of ten patients exhibiting successful response. Two patients were lost to follow-up and two experienced treatment failure.

**Conclusions:** Posaconazole salvage therapy demonstrates an 80% success rate in Mucormycosis patients with haematological malignancies. Although mild to moderate adverse drug events were reported, they did not necessitate discontinuation of therapy. Posaconazole is a safe and effective long-term salvage therapy with good tolerability.

**Keywords:** posaconazole, salvage therapy, mucormycosis, amphotericin B

# Abstract: Intravenous Proton Pump Inhibitors (PPI) Use for Stress Ulcer Prophylaxis (SUP) in Critically Ill Patients

**Authors:** Saba Naureen, Waseem Ullah Khan, Mahnoor Asad, Márió Gajdács, Lua Pei Lin, Long Chiau Ming, Suresh Shanmugham, Shazia Jamshed

**Affiliations:** 1 Pharmacy Advisor, Community Pharmacy, Boots, Manchester, United Kingdom; 2 Shifa College of Pharmaceutical Sciences, Shifa University, Islamabad, Pakistan; 3 Department of Pharmacy, Boitekanelo College, Gaborone, Botswana, Southern Africa; 4 Department of Oral Biology and Experimental Dental Research, Faculty of Dentistry, University of Szeged, Hungary; 5 Pharmacy Practice, Faculty of Pharmacy, Universiti Sultan Zainal Abidin, Terengganu, Malaysia; 6 School of Medical and Life Sciences, Sunway University, Kuala Lumpur, Malaysia; 7 Pharmacy Practice, School of Pharmacy, IMU, Kuala Lumpur, Malaysia; 8 Jinnah Women University, Karachi, Pakistan

**Objectives:** Acid suppressive medicines (ASM), especially proton pump inhibitors (PPIs), have been overused for stress ulcer prophylaxis (SUP) and often continued after discharge. The main aim of this study was to evaluate the current practices of IV use of PPIs in critically ill patients.

**Methods**: A retrospective single-centre study was conducted to evaluate the current practices of IV use of PPIs in critically ill patients at Shifa International Hospital, Islamabad, Pakistan. All adult patients admitted to the critical care wards of the hospital from January 2020 to December 2022 were included. Data about primary diagnosis, past medical history, past medication history, current medications in the hospital, appropriateness of SUP, and discharge medications were extracted from the electronic medical record (EMR). Demographics like gender, age, weight, and length of stay were also recorded. The primary outcome was to evaluate the current practices of IV PPIs for SUP in critically ill adult patients. The secondary outcomes were to determine the appropriate use of SUP and the associated factors for using SUP.

**Results and Discussion:** A total of 1076 patients’ data were reviewed. Eight hundred seventy-three patients were given SUP during ICU stay, omeprazole being the most frequently prescribed agent, accounting for 866 patients out of 1076. Around 81.7% were given SUP therapy in the medical ICU (MICU), 46.9% in the medical ICU step-down (MICU (SD)), and 67.2% in the surgical ICU (SICU). The medicines were continued after discharge in 22.2%, 11%, and 41% of patients in MICU, MICU (SD), and SICU, respectively. A total of 238 patients were discharged on the PPIs. The ‘minor risk factors’ and ‘SUP therapy continued after discharge’ showed statistically significant findings (0.013 and 0.0001, respectively). More than 50% of the patients (729; 67.75%) received inappropriate SUP therapy.

**Conclusion:** Overuse of IV PPIs in SUP in the adult ICU population was observed. Omeprazole was the most frequently prescribed agent. The prescribing practices did not follow ASHP guidelines. Moreover, an appreciable number of ICU patients were also discharged with prophylactic PPI.

**Keywords:** critically ill patients, stress ulcer prophylaxis, proton pump inhibitors

# Abstract: Evaluation of the Impact of the Covid-19 Pandemic on the Rise in TB Cases in Indonesia

**Authors:** Nurhasan, Diana Laila Ramatillah

**Affiliations:** Pharmacy Faculty, University 17 Augustus 1945 Jakarta, Jakarta, Indonesia

**Objectives:** This study aimed to identify and quantify changes in the number of TB cases detected before, during, and after the COVID-19 pandemic in Indonesia. Specifically, it assessed the association between the COVID-19 vaccine, environmental hygiene and nutritional intake.

**Methods:** This study used a retrospective observational design. TB case data were collected from various sources, including the Indonesian Ministry of Health's TB Dashboard. Data were analyzed to identify trends in the number of TB cases and to assess the association between TB cases and independent variables by conducting surveys and patient medical record reviews.

**Results and Discussion:** The results showed the influence of the COVID-19 pandemic on the increase in TB cases in Indonesia. The number of TB cases before the pandemic was 570,289 in 2018 and 568,987 in 2019; during the pandemic, 393,323 in 2020, 443,235 in 2021, and after the pandemic, 724,309 in 2022 and 821,200 in 2023. Additionally, the analysis showed that the COVID-19 vaccine did not affect increasing TB cases. The COVID-19 vaccine could increase immunity and reduce the risk of TB transmission. Malnutrition might be associated with an increased risk of Tuberculosis (TB) infection from passive to active. The effect of improved nutrition was analyzed as a contributing factor to the decline in TB cases in Europe in the 20th century, although this analysis was not based on data on changes in BMI in the population. The quality of the home environment that did not meet health requirements was a risk factor for TB disease transmission. The source of disease transmission was closely related to housing sanitation conditions, including ventilation, lighting, occupancy density, and room humidity.

**Conclusions:** This study provided valuable insight into the effect of the pandemic on the increase in TB cases in Indonesia. The COVID-19 vaccine did not affect the increase in TB cases, while environmental hygiene and nutritional intake significantly correlated with the cases. Cross-sector collaboration was needed to produce policies to address the pandemic and reduce its impact on TB control.

**Keywords:** Tuberculosis, Covid-19 Vaccine, Environmental Hygiene, Nutritional Intake

# Abstract: Factors Causing Drug-Related Problems A Mong Type 2 Diabetes Mellitus Patients

**Authors:** Maryanti Tuharea, Nurjianti Ipaenim, Diana Laila Ramatillah

**Affiliations:** Department Faculty, Universitas 17 Agustus 1945 Jakarta, Jakarta Indonesia

**Objective:** Type 2 Diabetes Mellitus patients are more susceptible to drug-related problems (DRP). Undesirable events that happen to patients undergoing drug therapy and have the potential to interfere with the expected success of healing are known as Drug-related Problems (DRP). In practice, many factors cause DRP, so the author wants to know the factors that cause DRP in Type 2 Diabetes Mellitus patients.

**Methods:** This research design was a systematic literature review of journals or articles via Google Scholar and Science Direct. This research was carried out following the PRISMA statement based on inclusion criteria, namely journals for 2018–2024; the outcome studied was the potential for Drug-Related Problems, this journal or article is open access, the journal or article has a design qualitative research and using Indonesian and English.

**Results and Discussion:** This research design was a systematic review by systematically looking at journals and articles in 300 articles and journals. There were seven articles and journals that met the inclusion and exclusion criteria. The risk factors that cause DRP are described into ten factors. Among them, drug interaction factors, especially pharmacodynamic interactions, play an essential role in causing DRP, followed by the number of DRP events reported in 6 research articles. Then, the factor of inaccurate drug doses or being too high or too low was reported in 5 articles, which caused treatment to be ineffective and caused side effects, factors such as age, gender, inappropriate selection of diabetes mellitus drugs, and factors indicating diabetes mellitus without receiving therapy. Diabetes mellitus drugs were reported in 3 articles, and polypharmacy factors were reported in 2 articles, which are significant factors causing DRP in geriatric patients with type 2 diabetes mellitus. Adverse drug reactions, drug side effects, and lifestyle factors were reported respectively in 1 article.

**Conclusions:** There is a strong correlation between inaccurate dosage and potential drug interactions, indicating that as the number of drugs used increases, the possibility of DRP also increases significantly.

**Keywords:** Drug-related problems, type 2 diabetes mellitus, systematic review

# Abstract: Effectiveness of pharmacist counselling on medication adherence of patients with diabetes mellitus type 2: A Systematic Review

**Authors:** Paulina B. Pelasula, Diana Laila Ramatillah, Mience Ubyaan

**Affiliations:** Pharmacy Faculty, Universitas 17 Agustus 1945 Jakarta, Jakarta, Indonesia

**Objective:** This study assessed the effect of pharmacist counselling on patient compliance and measured the impact of compliance on patient glycemic control.

**Methods**: This literature review uses the PRISMA method of the 176 journals found through the Publish and Perish app on Google Scholar. The literature search was conducted in May 2024. Systematic Reviews were conducted by searching for articles from Google Scholar, PubMed, ScienceDirect and Publish or Perish. Counselling conducted by a pharmacist, full text, counselling methods used, control groups that received counselling, control groups that did not, and research journals conducted in Asia in 2020–2024.

**Results and discussion:** Initially, 77 articles were removed due to duplicates and ineligibility determined by automation tools, thus leaving 99 journals, of which 47 were excluded and 32 were not received. Of the remaining 20 journals, 15 were excluded for various reasons: 6 lacked full text, 2 did not meet the period requirement, and 7 were not original articles. Ultimately, five journals were included in this systematic review. The analysis focused on the impact of pharmacist counselling on patient compliance and its subsequent effect on glycemic control. Various test methods were employed to analyze the data. The results showed a significant reduction in blood sugar levels in type 2 diabetes mellitus (DM) patients following pharmacist counselling, as evidenced by a paired t-test (*p* < 0.001). Furthermore, counselling significantly improved medication adherence, demonstrated by the Mann-Whitney test (*p* < 0.001) and the chi-square test (*p *= 0.004). The Morisky, Green, and Levine Medication Adherence Questionnaire (MGL MAQ) was used to assess adherence. Results indicated a significant improvement in medication adherence before and after pharmacist counselling, with a *p*-value of less than 0.001.

**Conclusion:** Further research on pharmacist counselling on therapy outcomes, diabetes complications and quality of life of patients with type 2 diabetes should be conducted. All articles collected and examined in this systematic review support the theory that pharmacist counselling can be very beneficial and significantly improves knowledge and medication adherence in patients with type 2 DM.

**Keywords**: Pharmaceutical Counselling, Type 2 Diabetes Mellitus, Patient Adherence

# Abstract: Community Pharmacists’ Knowledge and Perceptions of HPV Self-Sampling for Cervical Cancer Prevention

**Authors:** Sofia Umaira Binti Ismail, Nik Aisyah Najwa Nik Mustaffa Shapri, Zakiah Mohd Noordin, Aina Amanina Abdul Jalil, Nur Sabiha Md Hussin

**Affiliations:** 1 Department of Clinical Pharmacy, Faculty of Pharmacy, Universiti Teknologi MARA (UiTM), Selangor Branch, Selangor, Malaysia; 2 Faculty of Pharmacy and Health Sciences, Universiti Kuala Lumpur Royal College of Medicine Perak, 30450 Ipoh, Perak, Malaysia

**Objectives:** Community pharmacists (CPs) are well-positioned to promote HPV self-sampling due to their community accessibility, enabling them to provide knowledge access to kits and reduce the vaccination burden. However, their knowledge and perceptions of HPV self-sampling in Malaysia remain unclear. This study aimed to assess CPs’ knowledge and perceptions of cervical cancer, HPV infection, and HPV self-sampling and to explore the association with their sociodemographic characteristics.

**Methods:** A cross-sectional study was conducted from October 2023 to May 2024, involving 353 CPs across Malaysia. The CPs were invited to complete an online questionnaire through face-to-face interactions or online platforms. The questionnaire was divided into three sections: sociodemographic profiles, knowledge about cervical cancer, HPV, and HPV self-sampling, and perceptions of HPV self-sampling. Data were analysed using the Chi-square test and the independent sample t-test. A one-way ANOVA was employed to examine the statistical differences in participant perceptions based on sociodemographic variables, with a significance level set at *p*<0.05. Bloom's cut-off point was used to categorise the levels of knowledge and perception.

**Results and Discussion:** One hundred and fourteen CPs participated in the survey, yielding a 32.3% response rate. The mean knowledge score was 6.54/10, indicating a moderate level of knowledge, while the mean perception score was 3.64/5, also indicating a moderate level of perception. The findings highlighted CPs’ strong understanding of the benefits of HPV self-sampling in preventing cervical cancer, with a mean score of 4.04, and their recognition of the need for knowledge to actively promote it actively, scoring 4.26. The participants also emphasised the requirement for additional training and resources and the importance of policy reinforcement by the Ministry of Health and healthcare system experts, scoring 4.39. A significant difference was found between knowledge of cancer and family history of the disease (*p *< 0.001).

**Conclusions:** CPs demonstrated moderate knowledge and perception of HPV self-sampling for cervical cancer prevention, thus highlighting the need to address these gaps through stricter policies encompassing continuous education and awareness campaigns, mandatory training, and adequate allocation of resources.

**Keywords**: HPV, self-sampling, cervical cancer, knowledge, perception, community pharmacists

# Abstract: Analysis of Salt Consumption Pattern and Its Association with Prevalence of Hypertension Among Adolescent

**Authors:** Ni Putu Devi Wiraandini, Diana Laila Ramatillah

**Affiliations:** Pharmacy Faculty, Universitas 17 Agustus 1945 Jakarta, Jakarta, Indonesia

**Objective:** Hypertension is a significant health concern among adolescents, and high salt intake is one of the primary risk factors. This study aimed to understand the relationship between salt intake and hypertension among adolescents in Tangerang City, Indonesia.

**Methods:** A total of sixty-six participants were included in this analytical observational with a case-control design study: thirty-three cases of hypertension and 33 controls, with age and gender-matched in a 1:1 ratio. Participants were selected using consecutive sampling with inclusion criteria of being aged 15 to 18 years, having moderate physical activity, and having no family history of hypertension. This study measured blood pressure, calculated body mass index (BMI) by age, and evaluated salt consumption using a Semi-Quantitative Food Frequency Questionnaire (SQ-FFQ). The data collected were analyzed using the chi-square test with a 95% confidence level.

**Result and Discussion:** The data analysis showed that the hypertension case group had a higher salt consumption than the control group. About 48.5% of the participants were overweight, while 6.1% consumed high amounts of coffee. Participants who were overweight had a 6.13 times higher risk of developing hypertension. The bivariate analysis using the Chi-square test showed a significant relationship between nutritional status and high sodium food consumption with hypertension (*p*-value 0.001).

**Conclusion:** Our study results confirm the relationship between daily salt consumption, nutritional status, and blood pressure. Adopting a healthy diet and controlling salt intake can be crucial strategies in preventing adolescent hypertension.

**Keywords:** hypertension, adolescents, salt consumption, nutritional status

# Abstract: The relationship between patient education level and obedience to taking tuberculosis medicine with the success of tuberculosis therapy at primary health care Air Besar Ambon City

**Authors:** Surtia, Diana Laila Ramatillah, Nurhasan

**Affiliations:** Pharmacy Faculty, Universitas 17 Agustus 1945 Jakarta, Jakarta, Indonesia

**Objective:** The objectives of this study were to identify the patient's education level, measure compliance with taking tuberculosis medication, assess the success of tuberculosis therapy, analyze the relationship between education level and compliance with taking medication, analyze the relationship between compliance with taking medication and the success of therapy, and analyze the relationship between education level and the success of therapy.

**Methods:** This study is cross-sectional by design, and it was conducted prospectively and retrospectively, with data collected at the Air Besar Health Center, Ambon City. Primary data was collected using questionnaires to measure patient education level and medication adherence, and secondary data was collected from medical records, such as sputum test results, radiology, and other medical records, to obtain data on the success of tuberculosis therapy.

**Results and discussion**: Data was collected from 20 patients for this study. The findings indicate that six respondents (30%) demonstrated sufficient compliance with their medication regimen, while nine respondents (45%) showed good compliance, and five respondents (25%) exhibited excellent compliance. In this context, compliance refers to how patients follow doctors’ and nurses’ treatment and behavioural recommendations. It is crucial in the healthcare process, particularly in treating tuberculosis. The primary goals in treating tuberculosis patients include curing the disease, preventing death, preventing recurrence, and reducing transmission.

**Conclusion**: Knowledge, education, occupation, and attitude significantly influence medication compliance in patients with pulmonary tuberculosis.

**Keywords**: Education level, obedience to taking Tuberculosis medicine, success of tuberculosis therapy

# Abstract: The Impact Of The Hepatitis B Vaccination On Health Personnel's Ability To Defend Against Hepatitis B Virus

**Authors:** Mience Ubyaan, Diana Laila Ramatillah, Vania Jessica Ongkers

**Affiliations:** Pharmacy Faculty, Universitas 17 Agustus 1945 Jakarta, Jakarta, Indonesia

**Objective:** The main aim of this study was to examine and assess scientific literature and published findings from 2021 to 2024 that delve into the effectiveness of the Hepatitis B vaccine in healthcare settings, specifically focusing on healthcare workers.

**Methods:** The authors conducted a systematic literature review using Google Scholar and Scopus databases. They utilized PICO (Population/Problem, Intervention, Comparison, and Outcome) criteria to identify relevant articles. Only papers meeting the inclusion criteria were chosen for analysis. This approach was designed to ensure a comprehensive and impartial evaluation of the vaccine's efficacy.

**Results and discussion**: The analysis uncovered that the rate of complete Hepatitis B vaccination among healthcare workers was surprisingly low, with only one in five individuals receiving all required doses. Coverage is notably linked to gender, education level, work experience, infection prevention training, and bodily fluids and blood exposure. According to CDC recommendations, the vaccine schedule consists of three doses, with protection levels, increasing from 30 to 55% after the first dose, 75% after the second dose, and up to 90% after the third dose in adults over 40 and this underscores the significance of finishing the entire vaccination series for optimal protection. Approximately 70.000 infections occur yearly, with 5.9% of healthcare workers globally being exposed to Hepatitis B annually.

**Conclusion**: The study concluded that achieving full vaccine coverage among healthcare professionals was inadequate nationally, indicating a necessity for regular immunization protocols and increased vaccine acceptance through training. It was advised that healthcare workers undergo training on infection control, especially concerning Hepatitis B, to enhance vaccination coverage and public health. Immunization against the virus can effectively safeguard healthcare workers from Hepatitis B, underscoring the importance of vaccination in lessening occupational hazards.

**Keywords:** Vaccination health personnel’s, Hepatitis B, Vaccination, Virus

# Abstract: Challenges in Endometriosis Care: Treatment Adherence and Diagnostic Delay Analysis

**Authors:** Sadia Shakeel, Shagufta Nesar, Muhammad Ali, Mahnoor Asad, Shazia Jamshed

**Affiliations:** 1 Department of Pharmacy Practice, Faculty of Pharmaceutical Sciences, Dow College of Pharmacy, Dow University of Health Sciences, Karachi, Pakistan; 2 Jinnah College of Pharmacy, Sohail University, Karachi, Pakistan; 3 Faculty of Pharmacy, Salim Habib University, Karachi, Pakistan; 4 Rawal General and Dental Hospital, Islamabad, Pakistan; 5 Pharmacy Practice, School of Pharmacy, IMU, Kuala Lumpur, Malaysia; 6 Jinnah Women University, Karachi, Pakistan; 7 Shifa College of Pharmaceutical Sciences, Shifa Tameer-e-Millat University STMU, Islamabad, Pakistan

**Objective:** The present study aimed to explore physicians’ adherence to clinical practice guidelines for endometriosis assessment and care.

**Methods:** The current cross-sectional study targeted the general physicians and gynaecologists working in Karachi's different public and private patient care settings. Convenience sampling was used for the study. The questionnaire was designed based on the American Society of Reproductive Medicine (ASRM) guidelines and the European Society of Human Reproduction and Embryology (ESHRE). Along with the demographic information, the survey form included close-ended items to evaluate the physicians’ compliance with the international evidence-based guidelines for assessing and managing endometriosis.

**Results and Discussion:** The overall response rate of the present study was 72.5%, with a mean age of respondents 37.2 ± 7.5 years. More than half of the respondents (55.8%) stated the approximate prevalence of endometriosis in Pakistan as 11–20%. ASRM classification was the most commonly used measure for assessing the severity of endometriosis by (69.1%) respondents; however, the response rate varied significantly with their experience (*p *= 0.0001). More than half (67.3%) of respondents considered that endometriosis diagnosis is complex and stated that misinterpretation of symptoms (44.8%) was the most crucial factor for the diagnostic delay. About 46% of the respondents specified using clinical indications to identify endometriosis. Almost all respondents (97.5%) considered laparoscopy the gold diagnosis standard. The majority of the respondents (78.1%) stated that they counsel women assumed to have endometriosis symptoms and empirically treat them with analgesia combined with hormonal therapies. However, 66.9% take patient preferences, side effects and cost-effectiveness into consideration when selecting hormonal treatment for endometriosis-associated pain.

**Conclusions:** The outcomes revealed a need to establish plans to raise knowledge of endometriosis among physicians, shorten the time it takes to diagnose cases, and, ultimately, better manage women with the condition.

**Keywords:** Endometriosis; Physicians; Gynecology; Diagnosis; Pakistan.

# Abstract: Impact of Community Pharmacists’ Involvement in Public Health Services: Are they willing to participate in the intervention?

**Authors:** Sadia Shakeel, Hina Rehman, Márió Gajdács, Shazia Jamshed

**Affiliations:** 1 Department of Pharmacy Practice, Faculty of Pharmaceutical Sciences, Dow College of Pharmacy, Dow University of Health Sciences, Karachi, Pakistan; 2 Department of Pharmacy Practice, Institute of Pharmaceutical Sciences, Jinnah Sindh Medical University, Karachi, Pakistan; 3 Department of Oral Biology and Experimental Dental Research, Faculty of Dentistry, University of Szeged, Hungary; 4 Pharmacy Practice, School of Pharmacy, IMU, Kuala Lumpur, Malaysia; 5 Jinnah Women University, Karachi, Pakistan; 6 Shifa College of Pharmaceutical Sciences, Shifa Tameer-e-Millat University STMU, Islamabad, Pakistan

**Objective:** The current study was carried out to explore the pharmacists’ willingness to be involved in public health services and the self-perceived barriers that could limit their participation in the execution of such services.

**Methods:** The current cross-sectional study was conducted by adopting a pre-validated questionnaire distributed through different social media platforms to pharmacists in different community pharmacy settings and other patient care areas in Karachi. Convenience sampling was used to choose the participants. The pharmacists registered with the Pakistan Pharmacy Council and working during business hours from both genders were included in the sampling frame.

**Results and Discussion:** The response rate of the present study was 81.7%. More than half of the respondents were male. Most respondents (85.7%) showed a willingness to provide health promotion services; however, only 66.8% opined that professional curricular training is adequate. The respondents (84.1%) negated that pharmacists should not be involved in public health activities. Around 21% contemplate that society will not accept their participation in such activities. Lack of adequate training (58.8%), lack of patient interest in preventive healthcare activities (51.2%) and lack of financial reward (47.5%) were the major self-perceived obstacles that could limit the pharmacist's health promotional activities in the community pharmacy settings.

**Conclusions:** The current study's findings will close the gap in the literature in Pakistan and provide public healthcare stakeholders with information about pharmacist readiness to participate in community pharmacy-based public health services and potential obstacles.

**Keywords:** Community pharmacy; Public health activities; Pharmacists' willingness; Pakistan.

# Abstract: Assessment Of Prescriber's Awareness And Adherence To The Standard Treatment Guidelines Of Rheumatoid Arthritis In Sargodha, Pakistan

**Authors:** Saira Azhar, Shahar Yar, Jahanzaib Ahmed, Mahnoor Asad, Shazia Jamshed

**Affiliations:** 1 Faculty of Pharmacy, Iqra University North Campus Karachi, Pakistan; 2 College of Pharmacy, University of Sargodha, Sargodha, Pakistan; 3 Rawal General and Dental Hospital, Islamabad, Pakistan; 4 Pharmacy Practice, School of Pharmacy, IMU, Kuala Lumpur, Malaysia; 5 Jinnah Women University, Karachi, Pakistan; 6 Shifa College of Pharmaceutical Sciences, Shifa Tameer-e-Millat University STMU, Islamabad, Pakistan

**Objectives:** Effective rheumatoid arthritis (RA) management relies on prescribers’ adherence to standard treatment guidelines (STGs). This study aimed to assess prescribers’ awareness and adherence to STGs and identify factors influencing guideline implementation in Sargodha, Pakistan.

**Methods:** A descriptive cross-sectional study with a self-administered questionnaire, aligned with ACR and EULAR recommendations, was conducted among 203 prescribers (house officers, medical officers, specialists, and GPs) in public and private settings (100% participation rate). Descriptive statistics and inferential statistics were performed on SPSS version 23.

**Results and Discussion:** Over 60% of prescribers agreed with the efficacy of available RA medications and the cost-effectiveness of guideline-based prescribing. Only 33% were aware of the current Methotrexate monotherapy vs Hydroxychloquine/Sulfasalazine recommendation. A clear majority (79%) acknowledged the need for improved guideline awareness and adherence through educational programs. Prescriber-reported barriers in guideline adherence included patient-related issues (92%), lack of readily available guidelines (82%), and limited guideline enforcement (73%). Despite these challenges, most of the prescribers displayed awareness of appropriate treatments for RA, with significant agreement on the effectiveness of NSAIDs (Naproxen/Celecoxib), conventional DMARDs (Methotrexate/Sulfasalazine), and biologic DMARDs (Etanercept). Over 87% also recognized Prednisone's efficacy, and over half demonstrated awareness of proper standard treatment regimens for these medication classes.

**Conclusion:** Although prescribers acknowledged the value of STGs and demonstrated some awareness of appropriate RA treatments, gaps in knowledge and adherence existed. Educational programs, readily accessible guidelines, and improved enforcement mechanisms are crucial to bridge these gaps and optimize RA management in Sargodha.

**Keywords:** Rheumatoid arthritis; prescriber; awareness; treatment guidelines

# Abstract: The Influence of Knowledge and Attitudes of Health Workers on the Use of Antibiotics

**Authors:** Sari Unayah Hatuwe, Diana Laila Ramatillah, Meiyoritha S molle

**Affiliations:** Faculty of Pharmacy, University 17 August 1945 Jakarta, Jakarta, Indonesia

**Objectives:** The primary goal of this investigation is to explore the influence exerted by the knowledge and attitudes of healthcare professionals on the utilization of antibiotics.

**Methods:** A systematic literature review was conducted, utilizing search engines like Google Scholar and Scopus, focusing on publications within the last five years (2019–2024) that relate to the correlation between antibiotic resistance and antibiotic usage in the community.

**Results and Discussion:** A total of 190 articles were identified through an extensive literature search, of which 36 were considered relevant following screening and removing duplicates. Ultimately, 20 articles meeting the inclusion criteria were chosen. Only 31.3% of the participants reported receiving guidance on the prudent use of antibiotics, with half adjusting their perspectives post-receiving the information. The average score for knowledge concerning antibiotics stood at 2.6 out of 4, with 32.5% answering all knowledge-based queries accurately.

**Conclusion:** This research underscores the crucial role played by healthcare professionals in addressing antibiotic resistance, underscoring the necessity for tailored training initiatives and comprehensive resources to enhance their grasp of antibiotic resistance and suitable prescription practices. Continuous educational endeavours and awareness campaigns are imperative in bridging knowledge disparities among healthcare providers, promoting adherence to protocols, and fortifying the overall response to antibiotic resistance within healthcare settings.

**Keywords:** Attitude, Antibiotic Resistance**,** knowledge

# Abstract: An Analysis of Effectiveness of Monotherapy Versus Antipsychotic Polypharmacy Use on Quality of Life in the Treatment of Schizophrenia

**Authors:** Mariance Trisnawati, Diana Laila Ramatillah, Sari Unayah Hatuwe

**Affiliations:** Pharmacy Faculty: Universitas 17 Agustus 1945 Jakarta, Jakarta, Indonesia

**Objectives:** The study's objective is to perform a systematic literature review to assess the efficacy of monotherapy versus polypharmacy with antipsychotic drugs in treating symptoms of schizophrenia. The goal was to identify the most appropriate medications and doses for each patient to enhance treatment outcomes and improve their quality of life.

**Methods:** Following PRISMA guidelines, a systematic literature review was done using Google Scholar and Pubmed databases. The focus was on studies investigating the impact of monotherapy and antipsychotic polypharmacy on the quality of life of individuals diagnosed with schizophrenia. Only studies published in English within the past five years (2019–2024) and available in reputable journals were considered.

**Results and Discussion:** After the selection process, 20 research articles were identified and reviewed. The results indicated a preference for monotherapy antipsychotics due to their lower incidence of side effects. Both first-generation (typical) antipsychotics like Haloperidol and second-generation (atypical) antipsychotics such as Risperidone, Olanzapine, and Clozapine were found to manage symptoms of schizophrenia effectively. Polypharmacy is a consideration when monotherapy falls short in achieving desired outcomes to reduce severe side effects like metabolic syndrome and extrapyramidal symptoms, potentially leading to improved overall health and quality of life. Patient adherence tends to be higher with monotherapy, as managing multiple medications in polypharmacy can be challenging.

**Conclusion:** Monotherapy was established as the preferred initial treatment for schizophrenia, reserving polypharmacy for cases where monotherapy was ineffective. The choice between monotherapy and polypharmacy should be personalized, taking into consideration the patient's specific symptoms, treatment history, and close monitoring for potential side effects.

**Keywords:** Schizophrenia, monotherapy, polypharmacy, antipsychotics, effectiveness, quality of life

# Abstract: A Review of Cooking Oil Manufacturing No Free Fatty Acids (FFA) Using Free Fatty Acid Esterification and Enzymatic Methods

**Authors:** Ahmad sairi, Diana Laila Ramatillah

**Affiliations:** Pharmacy Faculty, Universitas 17 Agustus 1945 Jakarta, Jakarta, Indonesia

**Objective:** In this review, we will discuss cooking oils that do not contain free fatty acids and the dangers of free fatty acids for health

**Method:** A comprehensive literature review was conducted using sources such as Google Scholar, Science Direct, and PubMed, focusing on free fatty acids in cooking oil products and their health implications. Articles published between 2013 and 2023 were included based on relevance and discussion content, excluding those that did not address free fatty acids.

**Results and Discussion:** One way that can be done to remove free fatty acids is to react free fatty acids with alcohol with the help of a sulfuric acid catalyst. This reaction is known as esterification. The best condition in the research was using a catalyst percentage of 1.4% with a reaction time of 130 minutes and a free fatty acid content of 2.23%. Traditionally, FFA removal can use an enzymatic reaction from papaya sap and a fermentation process using the microbe Rhizopus oligosporus, often known as tempeh yeast. The lowest free fatty acid content was at pH 4 in the treatment of 0.4 grams of crude papain enzyme and 0.5 grams of tempeh yeast, 0.0491%. Meanwhile, the highest fatty acid content was in the treatment of 0.5 grams of tempeh yeast: 0.8 grams of crude papain enzyme at pH 3 of 0.1996%.

**Conclusion:** From the Journal literature, it can be concluded that the levels of fatty acids in cooking oil/coconut oil can be reduced using two methods, namely: This reaction is known as esterification using alcohol and sulfuric acid As well as using an enzymatic reaction from papaya sap and a fermentation process using the microbe Rhizopus oligosporus which is often known as tempeh yeast.

**Keywords:** Cooking / Coconut Oil, Free Fatty Acid, Esterification, Enzymatic.

# Abstract: Usage Pattern of Anti-hypertensive Medications in a District Hospital of Central Sarawak

**Authors:** Arthur Tiong Ing Jie, Chuo Sing Hong, Winnie Lew Ee Ling, Theng Sie Ching

**Affiliations:** Department of Pharmacy, Hospital Kanowit, Sarawak

**Objectives:** This study aimed to investigate the usage of anti-hypertensive medications in Hospital Kanowit, a district hospital in central Sarawak, specifically to assess the prescribing pattern and expenditure of anti-hypertensive medications in Hospital Kanowit.

**Methods:** We conducted a cross-sectional study by extracting data using the Pharmacy Information System (PhIS). Records on anti-hypertensive medications prescribed to adult patients (≥ 18 years old) from an outpatient clinic in Hospital Kanowit in 2021 were generated and analysed. We collected data on age, gender, number and types of anti-hypertensive medications prescribed. The cost of anti-hypertensive was calculated based on the total usage and price list provided by Logistic Pharmacy. Data was presented in frequency and percentages.

**Results & discussion:** 6133 patients were prescribed anti-hypertensive medications in Hospital Kanowit in 2021, with 56.90% female and primarily elderly patients with a mean age of 61.43. While 33.55% of the patients were on monotherapy (33.55%), the majority of the patients required at least dual anti-hypertensive therapy (34.84%) followed by triple therapy (19.56%). Most younger patients (<40 years old) only required monotherapy. However, 65.78% of patients between 40 and 65 years old and 70.05% of patients over 65 required at least dual therapy. Amlodipine was the most commonly prescribed anti-hypertensive medication to outpatients in Hospital Kanowit, followed by Perindopril (47.42%), Metoprolol (22.29%) and Bisoprolol (7.80%). In terms of expenditure, Hospital Kanowit spent RM425,362.96 on anti-hypertensive medications, constituting 22.10% of overall drug budget received in 2021. Despite Amlodipine being the most commonly prescribed anti-hypertensive medication, Metoprolol contributed to the highest expenditure (25.73%) among all anti-hypertensives, followed by Perindopril (21.27%) and Prazosin (15.80%).

**Conclusion:** Amlodipine was the most commonly prescribed anti-hypertensive medication in Hospital Kanowit. However, Metoprolol consumed the highest expenditure among all anti-hypertensive medications. It is recommended that the choice of beta blockers used in Hospital Kanowit be reviewed to ensure cost-effectiveness.

**Keywords:** Anti-hypertensive, Hospital Kanowit, prescribing pattern, expenditure

# Abstract: A Review on the Evaluation of Medication Management in Community Health Centers

**Authors:** Amanda Titirloloby, Florentina Sorluri, Srigemawati Singerin, Diana Laila Ramatillah

**Affiliations:** Pharmacy Faculty, Universitas 17 Agustus 1945 Jakarta, Jakarta, Indonesia

**Objective**: The study aims to ensure optimal medication management within Community Health Centers, focusing on the correct type, quantity, storage, distribution, use, and quality of medicines. The Regulation of the Minister of Health of the Republic of Indonesia No. 73 of 2016 provides standards for pharmaceutical services, guiding the planning, requesting, receiving, storing, distributing, destroying, withdrawing, controlling, administering, and evaluating drug management practices.

**Methods:** The study literature review for data collection to evaluate the drug. Five articles were reviewed based on an online database search. Articles published in Google Scholar between 2020 and 2023 were identified by searching the Garuda Portal. The search results met the specified keywords and inclusion criteria and were analyzed using the STROBE instrument.

**Results and Discussion**: The literature review found that the level of implementation of pharmaceutical service standards at community health centres was included in the excellent category, with an average score of 78–84%. The categories with the highest scores were the pharmaceutical preparation management category (84%) and the drug information service category (82%). The lowest score was in the quality assurance category (78%).

**Conclusion**: Based on the results of the systematic review, the results of the evaluation of drug management at the community health centre are in a suitable category.

**Keywords**: Evaluation, Drug Management, Community Health Center

# Abstract: Prevalence and Risk Factors of Lost To Follow-Up in Adult Patient on Antiretroviral Therapy: Narrative Review

**Authors:** Meiyoritha Sylvia Molle, Mariance Trisnawati, Diana Laila Ramatillah

**Affiliations:** Pharmacy Faculty, Universitas 17 Agustus 1945 Jakarta, Jakarta, Indonesia

**Introduction:** HIV/AIDS has become one of the leading causes of death in the world. By 2020, there is a 90-90-90 target, and one of the targets is that 90% of all people diagnosed with HIV infection will receive continuous antiretroviral therapy. HIV treatment with ART can experience several problems, one of which is loss of follow-up or LTFU. Knowledge of risk factors for LTFU can contribute to patient care, provide input for program policy, and reach the 90% target.

**Methods:** This narrative review synthesizes findings from recent studies on the risk factors associated with HIV LTFU. A comprehensive literature search was conducted using Google Scholar, Scopus, and PubMed for articles published between 2019 and 2024. Keywords used included “Risk factors for HIV Loss to Follow Up.” The review focused on open-access articles available in English and Indonesian.

**Results and Discussion:** Six articles were identified and reviewed. The analysis revealed several factors significantly influence LTFU, including age, knowledge, support and stigma, body mass index, access to health services, and ARV side effects. Social stigma often prevents patients from disclosing their HIV status, which negatively impacts adherence to ARV therapy. Additionally, inadequate family support is a significant factor leading to treatment discontinuation. Patients over 50 km from healthcare facilities face higher risks of LTFU due to limited access to health services.

**Conclusions:** Multiple factors contribute to the incidence of LTFU among HIV patients on ARV therapy. Understanding these risk factors is essential for healthcare providers and policymakers to develop effective strategies to reduce LTFU rates and improve the outcomes of ARV therapy.

**Keywords:** Prevalence, Risk Factors, Loss to follow-up, Antiretroviral therapy, Adult patient

# Abstract: An Analysis of Characteristics People Living with HIV (PLHIV) receiving Highly Active Antiretroviral Therapy (HAART) at Healthcare Clinics in Lembah Pantai, Kuala Lumpur

**Authors:** Mohemmad Redzuan, Syahrir Z, Norny Syafinaz R, Ramli M

**Affiliations:** 1 Pharmaceutical Service Programme, Ministry of Health Malaysia; 2 Kulliyah of Pharmacy, IIUM Kuantan, Malaysia; 3 Kulliyah of Medicine, IIUM Kuantan, Malaysia

**Objectives:** This study aims to analyze the characteristics of PLHIV undergoing Highly Active Antiretroviral Therapy (HAART) at Lembah Pantai, Kuala Lumpur healthcare clinics.

**Methods:** This retrospective study was conducted in September 2023, involving 748 PLHIV patients from two healthcare clinics in Lembah Pantai, Kuala Lumpur. Data collection forms were used to gather information from medical records and patient files. Descriptive analyses, including prevalence and mortality rates, were conducted to analyze the demographics of PLHIV in Lembah Pantai.

**Results and Discussion:** 89.7% (671) were male, and 85.3% (638) were single. The majority, 44.3% (331), were homosexual and got HIV infection from men sex with men (MSM). Overall, PLHIV had received HAART treatment for a median of 4 years and the age of diagnosed HIV median of 32 years old. 81% (606) were on first-line HAART treatment. The median CD4 count upon entry treatment was 301 cells/mm^3^, and the viral load was 1487 copies/mL. Prevalence PLHIV on chronic illness: diabetes (0.05%), hypertension (2.4%), dyslipidemia (8.8%), respiratory disease (2.1%) and underlying co-morbidities (7.2%). Prevalence on infectious disease: hepatitis (3.1%), tuberculosis (2.4.%) and hepatitis with tuberculosis (1.2%). Prevalence mental disorder: depression (1.7%), anxiety (1.7%), other mental disorders (0.5%). An analysis of all deaths occurred ten years with mortality (44.3%).

**Conclusions:** This study provides critical insight into the demographic and health characteristics of PLHIV on HAART in Lembah Pantai, Kuala Lumpur. The prevalence of diseases highlights the necessity for comprehensive healthcare approaches for PLHIV. Despite treatment advancements, the ten-year mortality rate remains high. These findings are crucial for developing targeted health policies and improving care for PLHIV.

**Keywords**: Characteristics, Highly Active Antiretroviral Therapy (HAART), Prevalence, Mortality, Healthcare.

# Abstract: The Impact of Augmented Renal Clearance on the Therapeutic Drug Monitoring of Vancomycin in an Infant with Invasive Pneumococcal Infection: A Case Report

**Authors:** Bakhtiar Siti Zakiyyah, Ahmad Narihan, Muhammad Ghazali

**Affiliations:** 1 Department of Pharmacy, Sarawak General Hospital; 2 Department of Paediatric, Sarawak General Hospital

**Objective:** This case report aims to highlight the impact of augmented renal clearance (ARC) on the vancomycin level in an infant.

**Methods:** We report a case of ARC in a 2-month-old boy with invasive pneumococcal infection. The patient was admitted for sepsis, initially treated with intravenous benzylpenicillin and cefotaxime. He developed myoclonic jerks, and subsequent lumbar puncture results were concordant with bacterial meningitis. The cerebral spinal fluid (CSF) sample analysed for meningitis panel via the multiplex polymerase chain reaction (PCR) was positive for *Streptococcus pneumoniae*. The initial blood culture grew penicillin-resistant (Minimum inhibitory concentration, MIC 2 mcg/mL), ceftriaxone-intermediate (MIC 1 mcg/mL) and vancomycin-susceptible (MIC 0.5 mcg/mL) *Streptococcus pneumoniae.* However, CSF culture had a negative yield. His brain's computed tomography (CT) showed the presence of left subdural effusion. Intravenous benzylpenicillin was discontinued and substituted with vancomycin. The patient completed a combination course of vancomycin and cefotaxime.

**Results and Discussion:** Throughout hospitalisation, the estimated glomerular filtration rate (eGFR) was predominantly above 130 mL/min/1.73 m^2^, consistent with the definition of ARC. Serum trough concentration of vancomycin was unattainable despite multiple loading doses and a maximum recommended dose of 60 mg/kg/day. As described in several studies, the standard vancomycin dosage may be insufficient in infants with ARC. However, little is known about the drug dosage recommendation in ARC, as the recommendation is variable and too general. In this case, the therapeutic range of vancomycin was only achieved after administration via continuous infusion and an increment to 80 mg/kg/day. Repeated lumbar punctures and brain imaging showed biochemical and radiological improvement. The patient completed vancomycin and cefotaxime for six and seven weeks, respectively, and was discharged home after 50 days of hospitalisation.

**Conclusions:** ARC is commonly associated with the subtherapeutic concentration of renal excreted drugs such as vancomycin. In critically ill paediatric patients, ARC needs to be considered, especially when antimicrobial efficacy is questioned. Optimising the pharmacokinetics of vancomycin, including therapeutic drug monitoring (TDM) and switching to continuous infusion, is paramount before considering switching to a broader spectrum antimicrobial.

**Keywords:** augmented renal clearance, vancomycin, therapeutic drug monitoring, critically ill infant, pharmacokinetic

# Abstract: Identifying Key Research Gaps in Malaysian Pharmaceutical Services: A Qualitative Thematic Analysis of Stakeholder Insights

**Authors:** See Wan Ho, Nor Ilham Ainaa Muhsin, Abdul Haniff Mohamad Yahaya, Pui Lim Chan, Azzy Iyzati Ahmad Shanizza, Siti Nur Su’aidah Nasarudin

**Affiliations:** Pharmacy Policy and Strategic Planning Division, Ministry of Health Malaysia

**Objective:** This study aimed to identify research gaps in pharmaceutical services in Malaysia for the next one to ten years to set the pharmacy research priorities in Malaysia. The research priorities are essential to steer pharmacy research activities within the country to generate data and evidence necessary to address crucial national health goals.

**Methods:** The study was carried out from January 2022 to December 2023. Input on research gaps was collected through workshops and online surveys involving a wide range of pharmaceutical services stakeholders in Malaysia, such as senior managers from the Pharmaceutical Services Programme of the Ministry of Health Malaysia and researchers, healthcare providers and representatives from both the public institutions and private pharmaceutical sectors. Literature and policy document reviews supplemented the data collection. The collected inputs were analysed using an inductive and deductive hybrid thematic analysis method. The thematic analysis was guided by an analytical framework created based on the pharmaceutical product life cycle and World Health Organization health system building blocks.

**Results and Discussion:** Seventy-four research gaps with 236 suggested research areas were collected. The thematic analysis identified eight themes and 24 sub-themes. The themes are Medicine development, Access to medicines, Impact of pharmacotherapy optimisation, Impact of pharmacy services, Medicine consumerism, Digital-in-health, Governance, and Capacity and capability building. These themes formed the basis for setting the pharmacy research priorities in Malaysia to guide the strategic planning of pharmacy research in the country.

**Conclusions:** This study identified critical research areas needing attention to improve pharmaceutical services in Malaysia. It underscored the importance of a systematic approach to identifying and analysing the research gaps. The involvement of diverse stakeholders ensured comprehensive insights, reflecting the needs and challenges across the healthcare system.

**Keywords**: pharmaceutical services, Malaysia, qualitative thematic analysis, research gaps, research priorities, stakeholder consultation

# Abstract: The Relation Of Antibiotic Use With Recurrence Of Acute Respiratory Tract Infection In The Hospital X

**Authors:** Mega Haryani, Ida Paulina Sormin, Liandhajani

**Affiliations:** 1 Magister Ilmu Farmasi, Fakultas Farmasi Universitas 17 Agustus 1945, Jakarta; 2 Fakultas Farmasi, Universitas Malaya, Malaysia

**Objective:** This study aims to examine the connection between the use of antibiotic therapy and the tendency to experience a recurrence of acute respiratory infection (ARI) among the adult population.

**Methods:** This observational study employs a retrospective cohort approach. Data were collected retrospectively through medical records at Hospital X. The relationship between antibiotic use and recurrence in ARI patients was analyzed using the Chi-Square test.

**Results and Discussion:** The total sample size was 110 patients. Among them, 53.6% received Cefixime therapy, making it the most common treatment. Cefadroxil was the second most frequently used therapy at 25.5%, followed by Azithromycin at 20.9%. The analysis indicated a significant association between antibiotic use and recurrence in ARI patients, with the Chi-Square test yielding a *p* < 0.037.

**Conclusions:** The Chi-Square test results (*p *< 0.05) indicate a significant relationship between the recurrence of ARI and the use of antibiotics such as Cefixime, Cefadroxil, and Azithromycin. This finding suggests that different antibiotics have varying impacts on the prevalence of recurrence in ARI patients.

**Keywords:** Antibiotics, Adults, Acute Respiratory Infection, Recurrence

# Abstract: The Correlation of Stress and the Incidence of Gastritris: Narrative Review

**Authors:** Srigemawati Singerin, Amanda Titirloloby, Florentina Sorluri, Diana Laila Ramatillah

**Affiliations:** Pharmacy Faculty, Universitas 17 Agustus 1945 Jakarta, Jakarta, Indonesia

**Objectives:** Gastritis, an inflammation of the stomach lining, can be exacerbated by stress due to the abnormal or imbalanced connection between the brain's nervous system and the stomach. This imbalance can lead to hormonal changes that stimulate excess stomach acid production, causing discomfort and bloating. Poor stress management can increase stomach acid levels and decrease prostaglandin levels. Individuals in their productive age often experience gastritis due to high levels of stress, busy lifestyles, and neglect of health. This study aims to determine the correlation between stress and gastritis through a narrative literature review.

**Methods:** This study employed a literature review methodology, examining five articles sourced from online databases. Data were collected by browsing the Garuda Portal and Google Scholar for articles published between 2011 and 2022. The search results were filtered based on relevant keywords and inclusion criteria, and the selected articles were analyzed using the STROBE instrument.

**Results and Discussion:** The literature review revealed that 34.5% of the respondents (180 out of 522) experienced mild stress, while 57.1% (290 respondents) reported incidents of gastritis. Statistical analysis showed a significant correlation between stress and the incidence of gastritis, with a *p*-value of <0.05.

**Conclusions:** The results of this literature review indicate a significant correlation between stress and the incidence of gastritis, highlighting the importance of stress management in preventing gastritis.

**Keywords:** Stress, Gastritis, Literature Review

# Abstract: Quitting at Work: Navigating Workplace-Based Smoking Cessation Intervention Challenges and Opportunities

**Authors:** Nor Syafiqah Binti Zunaidi, Syarifah Syamimi Putri Adiba Binti Syed Putera, Mohamad Haniki Nik Mohamed, Mahmathi Karuppannan, Zakiah Binti Mohd Noordin

**Affiliations:** 1 Faculty of Pharmacy, Universiti Teknologi MARA (UiTM) Selangor Branch, Puncak Alam Campus, 42300 Puncak Alam, Selangor, Malaysia; 2 Faculty of Pharmacy and Health Sciences, Royal College of Medicine Perak, Universiti Kuala Lumpur, Ipoh, Malaysia; 3 Department of Pharmacy Practice, Kulliyah of Pharmacy, International Islamic University Malaysia (IIUM), Jalan Sultan Ahmad Shah, 25200, Kuantan, Pahang, Malaysia

**Objectives:** In Malaysia, where smoking rates among working adults remain alarmingly high, there is an urgent need to address the lack of targeted workplace smoking cessation programs. This study delves into the complex landscape of workplace-based smoking cessation interventions (WBSCI) to uncover the challenges and opportunities that influence its adoption.

**Methods:** A qualitative study employing a semi-structured interview was conducted with 20 employers and employees in Klang, Selangor. Participants were individually interviewed through online platforms or in-person sessions, each lasting 20 to 30 minutes. Interviews were audio-recorded, transcribed, and analysed using thematic analysis and NVivo software.

**Results and Discussion:** The analysis identified five primary challenges: implementation costs, low smoker numbers, program structure, unfamiliarity with cessation programs, and smokers’ attitudes. Financial constraints and low smoking prevalence in some workplaces limit program prioritisation. Unfamiliarity with cessation methods and negative attitudes, such as unwillingness to quit, further hampered efforts. Three key themes were identified as enablers: designated smoking areas, health concerns, and existing smoke-free initiatives. The effectiveness of WBSCI is expected to be enhanced through the implementation of dedicated smoking areas, increased adoption of smoke-free initiatives, and improved personal motivation driven by health concerns.

**Conclusions:** This study provides valuable insights into the challenges and opportunities related to WBSCI. Successful implementation of WBSCI necessitates proactive, tailored strategies that consider population needs and available resources. Addressing these barriers and leveraging facilitators through collaborative efforts between responsible stakeholders are essential for improving workplace smoking cessation initiatives and fostering healthier work environments.

**Keywords**: smoking cessation, workplace, barriers, facilitators

# Abstract: Exploring the Practices and Factors Influencing Pre-Travel Consultations for Prospective Hajj and Umrah Pilgrims by Community Pharmacists: A Qualitative Study in Malaysia

**Authors:** Norazlin Abdul Kadir, Mohd Shahezwan Abd Wahab, Azyyati Mohd Suhaimi, Noordin Othman

**Affiliations:** 1 Faculty of Pharmacy, Universiti Teknologi MARA (UiTM) Selangor Branch, Puncak Alam Campus, 42300, Puncak Alam, Selangor, Malaysia; 2 Department of Pharmacy, Kuala Lumpur Health Clinic, Ministry of Health Malaysia, 53200, Kuala Lumpur, Malaysia; 3 Non-Destructive Biomedical and Pharmaceutical Research Centre, Smart Manufacturing Research Institute, Universiti Teknologi MARA (UiTM) Selangor Branch, Puncak Alam Campus, 42300, Puncak Alam, Selangor, Malaysia; 4 Faculty of Pharmacy, Universiti Sultan Zainal Abidin, Besut Campus, 22200, Besut, Terengganu, Malaysia; 5 Quality Use of Medicines in Umrah and Hajj Pilgrimage Research Group, Department of Pharmacy Practice, College of Pharmacy, Taibah University, 30001, Madinah, Saudi Arabia; 6 School of Pharmacy, Management and Science University, 40100, Shah Alam, Selangor, Malaysia

**Objectives:** This study explores community pharmacists (CPs) practices and the factors influencing pre-travel pharmacist consultations for prospective Hajj and Umrah pilgrims in Malaysia.

**Methods:** We conducted semi-structured interviews with 24 CPs in Selangor and Kuala Lumpur, Malaysia. The Theoretical Domains Framework (TDF) was used to develop the interview guide and analyse the data. These face-to-face interviews explored CPs’ practices and beliefs, focusing on the barriers and facilitators in providing pre-travel consultations for prospective Hajj and Umrah pilgrims. The audio recordings of the interviews were transcribed verbatim and analysed using qualitative content analysis.

**Results and Discussion:** We identified seven distinct activities performed by CPs, primarily providing travel health education. Beliefs regarding pre-travel pharmacist consultations for prospective Hajj and Umrah pilgrims were categorised into 14 domains of the TDF. The study shows that CPs were motivated to offer pre-travel consultations to the pilgrims. However, they also faced challenges, including knowledge gaps in Hajj and Umrah travel guidelines and health-related issues concerning the pilgrimage. Additionally, CPs reported barriers such as staff shortages, lack of time, limited confidence in providing consultations related to regulations and disease management, and a perceived lack of support from doctors.

**Conclusions:** This study offers valuable insights into the activities involved in pre-travel consultations conducted by CPs. The findings suggest interventions that can potentially increase engagement of pre-travel pharmacist consultations for prospective Hajj and Umrah pilgrims, including improved training, education, resources, and interprofessional collaboration.

**Keywords**: Community pharmacist, Hajj, Umrah, mass gathering, travel medicine, qualitative, theoretical domains framework, Malaysia

# Abstract: Review of Hair Growth and Weight of Hair Tonic Preparations from Several Herbs

**Authors:** Liandhajani, Guntoro Halim, Antonius Padua Ratu

**Affiliations:** 1 Department of Pharmacy, 17 August 1945 University Jakarta, Indonesia; 2 Department of Pharmacy, Sekolah Tinggi Teknologi Industri dan Farmasi, Bogor Indonesia

**Objectives:** Hair tonic is a hair care cosmetic formulated to help overcome hair loss problems. This study is to review the dosage form of a hair tonic with plant extracts that have the potential to maintain healthy hair.

**Methods:** The plant extracts to be reviewed green tea leaf (*Camellia sinensis* (L) Kuntze) *Sauropus androgynus* (L) Merr. Leaf; *Apium graveolens* L. and honey; black tea (*Camellia sinensis* (L) Kuntze); *Aloe vera* and *Glycyrrhiza glabra* L.; *Apium graveolens* L. and green tea leaf (*Camellia sinensis* (L) Kuntze); *Momordica charantia* L. leaf; *Polyscias scutellaria* leaf; *Pogostemon cablin* Benth. And *Ipomoea aquatica* Forsk. The dosage form is given to rabbits 1–2 times daily for 7–28 days. The hair growth results of each extract were tested statistically with ANOVA and Duncan's further tests using SPSS version 26.

**Results and Discussion:** The review results with Anova and Duncan divided the differences into five subsets of the dosage form with the extract. The dosage form of *Pogostemon cablin* Benth leaf extract showed a very significant difference compared to other dosage form extracts in the percentage of hair growth against negative controls. The dosage form with the water fraction of tea leaf extract and *Sauropus androgynus* (L.) Merr leaf also showed significant differences from other extracts. The percentage of hair weight increase was most significant in the preparation with *Momordica charantia* L leaf extract compared to extracts from green tea leaf (*Camellia sinensis* (L) Kuntze); *Apium graveolens* L. and honey; *Aloe vera*; and *Glycyrrhiza glabra* L.

**Conclusions:** The study concluded that the preparation with *Pogostemon cablin* Benth leaf extract, tea leaf, and *Sauropus androgynus* (L.) Merr leaf has the potential to provide hair growth; *Momordica charantia* L. leaf extract has the potential to increase hair weight.

**Keywords**: extract, hair growth, hair tonic, hair weight

# Abstract: Antianemia Therapy Patterns And Its Impact on the Clinical Outcomes Of Stage V Chronic Kidney Disease Inpatients of Dr. X General Hospital, Padang, Indonesia

**Authors:** Armenia, S.R. Annisa, D. Dillasamola

**Affiliations:** Faculty of Pharmacy, University of Andalas, Padang, West Sumatera, Indonesia

**Objectives:** Anemia in chronic kidney disease (CKD) is prevalent and significantly impacts patient quality of life. This study aims to determine the impact of the severity of anaemia, the pattern of anaemia therapy, the socio-demography, and the time of administration of antianemia therapy to the clinical outcome (Hb, Hct, and erythrocyte levels) on the stage V CKD patients

**Methods:** This retrospective observational analytics study was conducted on 119 inpatient data hospitalized in 2021 at the General Hospital Padang Indonesia. Data was analyzed by ANCOVA, followed by Duncan's multiple T range test. The significance was taken at *p *< 0.05.

**Results and Discussion**: The results showed that folic acid, PRC transfusion, and their combination were the predominant therapies for stage V CKD patients. All patients exhibited an average Hb, Hct and erythrocyte increase after therapy (*p *< 0.05). The patient sex and age did not significantly affect (*p *> 0.1), but the severity of anaemia and the therapy significantly (*p* < 0.05) affect the patient outcome. However, all outcome parameters are below average.

**Conclusions**: Folic acid, PRC infusion, and their combination were given to Stage V CKD patients, and they could not overcome anaemia.

**Keywords:** anaemia, stage V CKD, anaemia therapy, haematological parameters.

# Abstract: The Perception and Acceptance of Community Pharmacists Towards the Implementation of Zoning Policy: A Preliminary Study in Sarawak

**Authors:** Theng Wei Kiong, Loo Shing Chyi, Syahida Sarini Binti Bolhan, Debbie Lee Sui Ling, Rachel Sii Zu Wen, Cannilia Anak Kerine, Ting Lee Chyn, Chieng Sying Na, Sharifah Husna Aqilah Binti Syed Mohamad

**Affiliations:** Sarawak State Pharmacy Enforcement Branch, Ministry of Health Malaysia

**Objectives:** This study evaluated the perception and acceptance of community pharmacists regarding implementing zoning policies in Sarawak.

**Methods**: A cross-sectional quantitative survey was conducted from March to April 2024, utilizing a self-created questionnaire that underwent face and content validation, pre-testing, and pilot testing. The questionnaire demonstrated sufficient internal consistency, with Cronbach's Alpha values for questions on perceived benefits (0.95), disadvantages (0.91), challenges (0.70), and acceptance (0.64) towards pharmacy zoning policy, respectively. The study included all licensed community pharmacists in Sarawak, with a minimum sample size of 177. Descriptive statistics and multiple logistic regression were employed for analysis, with a *p*-value of <0.05 considered statistically significant.

**Results and Discussion**: A total of 81 pharmacists responded, with over 75% of them agreeing that the implementation of zoning policies would improve healthcare access, quality, and sustainability. Most of them perceived that zoning policy enables equitable access for urban and rural populations, mitigates unhealthy competition, and therefore allows pharmacists to prioritize service quality. The majority (74%) also acknowledged that effective design, implementation, and enforcement would pose significant barriers and challenges. Standardized regulations might not sufficiently address different healthcare requirements across regions, necessitating ongoing refinement and adaptation of policies. Nevertheless, 74.1% expressed agreement with implementing zoning policies in their practice areas, provided that minimum distance requirements (85.2%) and alignment with local health needs (66.6%) were adequately addressed.

**Conclusions**: Most community pharmacists perceive zoning policies as beneficial, although they anticipate challenges in implementation. Integrating minimum distance requirements and considering local health needs are critical factors for successful policy implementation.

**Keywords:** Community pharmacy, Zoning, Accessibility, Competition, Sustainability

# Abstract: Evaluation of the effectiveness of antibiotic use in infection and non-infection of type 2 DM patients at one of the internal medicine specialist clinics in Ambon City.

**Authors:** Grace Ivone Novaline Souhuwat, Diana Laila Ramatillah

**Affiliations:** Pharmacy Faculty, Universitas 17 Agustus 1945 Jakarta, Jakarta, Indonesia

**Objectives:** The study aimed to evaluate the increase in the rational use of antibiotics by reducing the incidence of infection and improving glycemic control to decrease complications and enhance the quality of life for patients with type 2 diabetes.

**Methods:** The study employed a quantitative, cross-sectional research design targeting type 2 diabetes mellitus (DM) patients undergoing antibiotic treatment for infectious and non-infectious conditions at an internal medicine specialist clinic in Ambon. Data collection involved questionnaires assessing educational levels and medication adherence, supplemented by medical record data and pharmacist counselling sessions to evaluate therapeutic outcomes. Statistical analyses, including correlation and regression, explored the relationships and impacts among the study variables.

**Results and Discussion:** Age, gender, and comorbidities were examined among 200 participants with type 2 DM receiving antibiotic treatments. The largest age group was 56–65 years, comprising 44.4% of the participants, while the youngest age group (36–45 years) accounted for 2.8%. The second largest age group was 46–55 years, representing 36.1%, with a notable decrease to 16.7% in participants over 65. Gender analysis revealed a higher prevalence of female diabetics (52.8%) compared to male diabetics (47.2%). A substantial correlation was observed between administering antibiotics in type 2 DM patients for infectious and non-infectious conditions. The data indicated a strong relationship between the rational use of antibiotics and reduced infection incidence, contributing to improved glycemic control. This reduction in infections subsequently decreased complications and enhanced the quality of life for patients with type 2 diabetes.

**Conclusions:** The study provided valuable insights into the effectiveness of antibiotic use in infection and non-infection patients with type 2 diabetes at an internal medicine specialist clinic in Ambon City. Findings revealed that most patients were aged between 56 and 65 years. The potential impact of antibiotic use due to infection was 25%, while the potential for non-infection-related antibiotic use was 2.8%. Age, gender, and comorbidities did not significantly influence the use of antibiotics in type 2 diabetes patients.

**Keywords:** Antibiotic, Infections, Type 2 diabetes mellitus

# Abstract: The Effectiveness of Daily Pre-packed Medication with Pictogram Labelling (DPM-PL) in Improving Medication Adherence of Haemodialysis (HD) Patients in Hospital Sibu

**Authors:** Ling Ding Ee, Hii Evangelin Hui Hui, Sii Pei Jen, Ting Grace Yien Ling, Mah Angelica Xiao Hei, Kong Min Xian, Hii Lawrence Wei Soon, Samuel Ting Chuo Yew

**Affiliations:** 1 Pharmacy Department, Hospital Sibu, Sarawak, Malaysia; 2 Pharmacy Department, Hospital Saratok, Sarawak, Malaysia; 3 Sarawak General Hospital, Sarawak, Malaysia; 4 Pharmacy Practice and Development Branch, Sarawak State Health Department, Malaysia

**Objective:** This study aims to assess the effectiveness of daily pre-packed medication with pictogram labelling (DPM-PL) in improving medication adherence among haemodialysis (HD) patients in Hospital Sibu.

**Methods:** A quasi-experimental study was conducted with 33 HD patients in Hospital Sibu. Those who took ≥6 oral medications with poor medication adherence defined by pill count (PC) <85% and Malaysian Medication Adherence Assessment Tool (MyMAAT) score <54 were recruited. After pre-intervention data collection, subjects’ medications were pre-packed with plastic sealer bags according to HD and non-haemodialysis day (NHD) regimens. It was generally split into three serving times of the day and with regard to meals. Each bag was labelled with a corresponding pictogram. Subjects were under follow-up at two-week intervals for a total of six weeks. Pre-intervention PC, MyMAAT, medications Dose, Frequency, Indication and Time of administration (DFIT) score, pre-HD blood pressure (BP), and serum phosphate levels were compared against post-intervention data. Data were analysed using paired t-test, Wilcoxon signed-rank test and repeated measures ANOVA test.

**Results and Discussion:** Regarding pill count, medication adherence significantly improved at week 4 (*p *= 0.02) and week 6 (*p *= 0.03). Post-intervention mean MyMAAT score (56.7 ± 3.91) was significantly higher than pre-intervention mean score (41.7 ± 9.49) [*t*(22) = 7.56, *p*<0.001]. Post-intervention DFIT median score [96.9(IQR = 7.3)] was significantly higher than the pre-intervention DFIT median score [91.7(IQR = 8.9)] (*z* = −4.05, *p*<0.05). Thus, DPM-PL intervention improved medication adherence and understanding, attributing this success to its simplicity. Nevertheless, the difference in pre-HD BP over time was not statistically significant (*p *= 0.908 for systolic BP, *p *= 0.761 for diastolic BP). Mean serum phosphate level decreased by 0.1 mmol/L post-intervention but was statistically non-significant (*p *> 0.05). Confounding variables such as excessive dietary sodium, phosphate and fluid intake may lead to insignificant outcomes.

**Conclusions:** The DPM-PL improved patients’ medication adherence and understanding over time. To fully understand the sustained impact of this intervention on clinical outcomes, additional studies with extended follow-up periods in larger haemodialysis (HD) populations are necessary.

**Keywords**: medication adherence, haemodialysis patient, pre-packed medication, pictogram labelling

# Abstract: Determining the cost elements for reimbursement of pharmaceutical services provided by community pharmacies: A scoping review

**Authors:** Nor I A Muhsin, Yi Huan Tan, See Wan Ho, Ellisya A Z Murad, Azzy I A Shanizza, Nuruz Z M Zin, Mohd A F Abdul, Hafidza Baharum, Siti N S Nasarudin, Pui Lim Chan, Abdul H M Yahaya

**Affiliations:** Pharmacy Policy & Strategic Planning Division, Ministry of Health, Malaysia

**Objectives:** This study aims to comprehend the cost elements for reimbursement of pharmaceutical services provided by community pharmacies in various countries. The outcomes of this study may assist policymakers in determining appropriate reimbursement rates for pharmaceutical services provided by community pharmacies in Malaysia.

**Methods:** A systematic scoping review following the PRISMA extension for scoping reviews (PRISMA-ScR) was conducted across PubMed, ScienceDirect and Cochrane databases from inception to August 2023 using pre-defined keywords relevant to cost elements for reimbursement and pharmaceutical services. Two independent researchers assessed the quality of each article before the data were synthesised qualitatively.

**Results and Discussion:** Out of 20,064 articles screened, 30 were included in this study across 17 countries. The study identified 27 unique cost elements, with labour, operational, and material costs being the most common. Significant regional variabilities were observed. Malaysia's pharmacist salaries dominated labour costs in Asia, while Thailand's labour costs varied by service type. Operational costs in Malaysia included monthly rent and utilities; in Oman, the cost for automation of prescription processing was included to reduce drug dispensing time.

Additionally, material costs in Malaysia encompassed office materials and utilities while Thailand covered daily dose dispensing. In Europe, labour costs vary by service and intervention types in Portugal and the UK, while in the Netherlands focused on operational costs per medication package and material costs covering essential utilities such as stationary, electricity, and water. Similarly, in Africa, South Africa's labour costs range widely based on service complexity, with operational and material costs linked to medical equipment and supplies. Globally, automation and systematic improvements reduced costs and enhanced efficiency, emphasising the need for standardised reimbursement structures.

**Conclusions:** Overall, the findings highlight labour, operational and material costs as the most common cost elements globally in pharmaceutical services. Policymakers should consider these elements when determining appropriate reimbursement rates for pharmaceutical services provided by community pharmacists in Malaysia.

**Keywords**: cost elements, reimbursement, pharmaceutical services, community pharmacies, scoping review

# Abstract: Current Landscape and Factors Influencing Market Competition in Malaysia’s Public Pharmaceutical Procurement System: Stakeholder Perspectives

**Authors:** Farahwahida Mohd Kasim, Ernieda Hatah, Lokhman Hakim Osman, Adliah Mhd Ali, Zaheer-Ud-Din Babar

**Affiliations:** 1 Centre of Quality Management of Medicines, Faculty of Pharmacy, Universiti Kebangsaan Malaysia, Kuala Lumpur, Malaysia; 2 Center of Valuation Creation and Human Well-being Studies, Faculty of Economics and Management, Universiti Kebangsaan Malaysia, Kuala Lumpur, Malaysia; 3 Pharmaceutical Services Programme, Ministry of Health, Petaling Jaya, Malaysia; 4 Centre of Pharmaceutical Policy and Practice Research, University of Huddersfield, United Kingdom

**Objectives:** To investigate stakeholders’ perspectives in the pharmaceutical industry on the current landscape and factors that may influence market competition in Malaysia's public pharmaceutical procurement system.

**Methods:** Qualitative semi-structured interviews were conducted with stakeholders from the Ministry of Health (MOH) (purchasers) and pharmaceutical companies (suppliers) between November 2023 and February 2024. Purposive sampling was used to invite stakeholders with extensive experience purchasing and supplying pharmaceuticals in the public procurement healthcare system. Participants shared their views on the current landscape and factors influencing market competition in the public pharmaceuticals procurement system. Interviews were audio-recorded, transcribed verbatim, and analyzed using thematic analysis with ATLAS.ti version 9.1.3.

**Results and Discussion:** A total of 26 participants were interviewed, including 18 purchasers from the Ministry of Health (MOH) sectors—such as headquarters, state central stores, hospitals, and health clinics—and eight suppliers from multinational and local pharmaceutical companies as well as tendering agents. Participants had between 4 and 40 years of experience in their fields. They view market competition in the public pharmaceutical procurement system as associated with supply and demand, pricing strategies, and the strategic evaluation of profitability, encompassing product and non-product criteria. Companies employ competitive strategies and market entry tactics, including competitor analysis and persuading prescribers of their drugs’ benefits. Factors influencing market competition in public pharmaceutical procurement systems include market power, regulatory frameworks, government procurement policies, and procurement efficiency. These elements may affect market competition, from initial entry and bidding participation to pricing strategies.

**Conclusions:** Market competition in the pharmaceutical public procurement healthcare system was intricate, dynamic, and influenced by multiple factors. Developing government policies and processes that promote healthy competition while ensuring the sustainability and viability of both government and industries is essential for protecting national and public interests.

**Keywords**: drugs, competitive market, government purchasing, pharmaceutical industries, views

# Abstract: A Novel Small Unilamellar Carrier System for Enhanced Skin Delivery of Red Ginger Extract as Anti-Inflammatory

**Authors:** Henny Lucida, Gilang Lukviarman, Nur Syarifatun Nisa, Netty Suharti

**Affiliations:** 1 Department of Pharmaceutics, Faculty of Pharmacy, Universitas Andalas, Padang, Indonesia; 2 Department of Pharmaceutical Biology, Faculty of Pharmacy, Universitas Andalas, Padang, Indonesia

**Objectives:** This study aims to optimize the formulation of red ginger extract niosomes and evaluate mice's physicochemical properties and anti-inflammatory effects.

**Methods:** Optimization was performed using a 2^5−^factorial design (5 factors, mole ratio of 6-gingerol in the extract: span 60: cholesterol, sonication time, hydration temperature and drying rate) with two levels (low and high). Niosomes were prepared using a thin layer hydration method followed by sonication, and the droplet size response was analyzed using Design Expert 13®. The anti-inflammatory activity of niosome gel containing 10% red ginger extract was assessed using the air pouch method to measure the exudate volume in mice.

**Results and Discussion:** The optimal niosome formulation was predicted at the composition of 1 mol 6-gingerol (equivalent to 2.1 g of extract): 1.0332 mol span 60: 0.1081 mol cholesterol sonicated for 15 minutes with a hydration time of 3.53 minutes at 56.87°C, a rotary evaporator speed of 204.60 RPM to obtain vesicle size of 28 nm. Niosome production at optimum conditions obtained spherical vesicles with a Z-average of 284.3 nm, a zeta potential of 73.7 mV, a PDI of 0.485, and an entrapment efficiency of 71.56%. Red ginger extract niosome gel reduced the average exudate volume in mice injected with 1% carrageenan, significantly different from the control groups (*p* < 0.05).

**Conclusions:** The niosomal delivery system enhances the anti-inflammatory activity of red ginger extract and potentially solves the problem of the ineffectiveness of conventional gels for relieving joint pain.

**Keywords**: red ginger, niosomes, optimization, anti-inflammatory

# Abstract: Exploring Factors for Pharmaceutical Price Variability in Segmented Procurement System of Malaysia's Public Healthcare: Insights on Market Competition

**Authors:** Farahwahida Mohd Kasim, Ernieda Hatah, Lokhman Hakim Osman, Adliah Mhd Ali, Zaheer-Ud-Din Babar

**Affiliations:** 1 Centre of Quality Management of Medicines, Faculty of Pharmacy, Universiti Kebangsaan Malaysia, Kuala Lumpur, Malaysia; 2 Center of Valuation Creation and Human Well-being Studies, Faculty of Economics and Management, Universiti Kebangsaan Malaysia, Kuala Lumpur, Malaysia; 3 Pharmaceutical Services Programme, Ministry of Health, Petaling Jaya, Malaysia; 4 Centre of Pharmaceutical Policy and Practice Research, University of Huddersfield, United Kingdom

**Objectives:** This study explores market competition by evaluating factors associated with price variability in the Ministry of Health (MoH) segmented pharmaceuticals procurement system.

**Methods:** This retrospective observational study analysed 2016 procurement data for high-cost pharmaceutical products in MoH, focusing on drugs from eight therapeutic subgroups. The purchase orders, local contracts, and supplier bid data from 2017 to 2021 were sampled from 19 MoH facilities across five geographical zones. Price variability was calculated as a percentage by dividing the observed price by the minimum price for brands within the same year. The Kruskal-Wallis H test, with Bonferroni correction, was used to identify differences between geographical zones, facility types, procurement values, therapeutic subgroups, product types, and the number of competing local agents and products.

**Results and Discussion:** A total of 98 drugs through 4,702 purchase orders managed by 28 local agents were included in the study. Significant variables indicate that East Malaysia exhibits higher price variability compared to Peninsular Malaysia (*p* < 0.001), while within Peninsular Malaysia, the Southern region shows higher price variability than the Northern region (*p *= 0.04). Hospitals and health clinics segmented purchasing do not show significant price variations (*p* > 0.05). Procurement values of RM50,000 to RM1 million (*p* < 0.001) and direct purchases of RM30,000 to RM50,000 (*p *= 0.03) had lower price variations than those under RM10,000, respectively. Lipid-modifying agents and immunosuppressants showed lower price variations than others (*p* < 0.001). Imported generics had more price variation than innovators, reference biologics, and local generics (*p* < 0.001). Purchases with three or more competing agents had lower price variation than single agents (*p* < 0.05), and quotes from three to five competing products showed lower price variation than two competing products (*p *= 0.037). The findings suggest that more suppliers or products stabilise prices while fewer competitors increase price variability.

**Conclusions:** The study highlights key factors associated with price variability and competition dynamics in the public's segmented pharmaceutical procurement system. A longitudinal analysis could offer deeper insights into market trends and procurement strategy effectiveness.

**Keywords**: drug; medicine; pharmaceutical; pricing; public’s segmented procurement; facility-based procurement

# Abstract: Overview Of Metadon Dose Use In Patients In The Methadone Maintenance Therapy Program (Ptrm) In Greater Tangerang

**Authors:** Melwani Amran, Diana laila Ramatillah

**Affiliations:** Magister Pharmacy, Faculty of Pharmacy, 17 Agustus 1945 University, Jakarta, Indonesia

**Objectives:** Methadone is utilized to help heroin addicts transition away from heroin by substituting it with methadone at an adjustable dose, which is gradually decreased until methadone can be discontinued. This study aims to determine the methadone dosages administered to patients in the Methadone Maintenance Therapy Program (PTRM) in Greater Tangerang from January to December 2023. The study includes methadone clinics at Cibodasari Community Health Center, Cipondoh Community Health Center, and Ciputat Community Health Center.

**Methods:** A cross-sectional, descriptive study design was employed. Data were collected from patient records at the three methadone clinics. Variables analyzed included patient demographics, initial and maintenance methadone doses, educational levels, and marital status.

**Results and Discussion:** The study included 33 patients, predominantly male (91%). Age distribution showed that 97% were between 25 and 45 years old, while 3% were between 46 and 55 years old. Educational attainment varied, with 18% having a junior high school education, 58% high school, 15% diploma, and 9% college education. Regarding marital status, 73% were married, 12% unmarried, and 15% previously married. Initial maintenance doses ranged from 15 to 30 mg/day. The smallest maintenance dose recorded was 0.7 mg/day, the highest was 210 mg/day, with an average maintenance dose of 76.1 mg/day.

**Conclusions:** The findings indicate that the initial methadone doses for patients in the Methadone Maintenance Therapy Program ranged from 15 to 30 mg/day. Maintenance doses varied widely, with a minimum of 0.7 mg/day, a maximum of 210 mg/day, and an average of 76.1 mg/day, aligning with PTRM guidelines recommending maintenance doses typically between 60 and 120 mg/day.

**Keywords:** Methadone, Maintenance Therapy, PTRM, Dosage, Community Health Centers

# Abstract: Use of Erythropoietin Drugs in Hemodialysis Practice for Chronic Kidney Failure Patients: A Systematic Review

**Authors:** Firly Kartika Yuni Rambi, Diana Laila Ramatillah

**Affiliations:** Pharmacy Faculty, Universitas 17 Agustus 1945 Jakarta, Jakarta, Indonesia

**Objective:** This study aims to review the literature on the use of erythropoietin drugs or therapy in patients with chronic kidney failure undergoing hemodialysis.

**Methods:** This research employs a systematic literature review using the PRISMA guidelines. Articles were identified based on inclusion and exclusion criteria. Inclusion criteria included publications in English or Indonesian, published between 2019 and 2024, and articles discussing hemodialysis, chronic renal failure, erythropoietin, and clinical pharmacy. Exclusion criteria included reports or draft policy guidelines from WHO or specific organizations, journals inaccessible in full text, and research articles on the psychological conditions of patients undergoing hemodialysis. Searches were conducted in the PubMed, ScienceDirect, and Google Scholar databases.

**Results and Discussion:** The search yielded 58 relevant articles. After screening titles and abstracts and reviewing full texts, ten articles met the inclusion criteria and were independently reviewed. The PRISMA flowchart was used to ensure a transparent selection process. The systematic review identified significant differences in the average increase in Hb levels pre- and post-administration of erythropoietin alfa (0.02 g/dl) and erythropoietin beta (0.48 g/dl), as proven by paired t-test statistical analysis.

**Conclusion:** The findings demonstrate the effects of erythropoietin therapy on Hb levels in hemodialysis patients with chronic renal failure.

**Keywords:** Erythropoietin, Hemodialysis, Chronic Renal Failure, Clinical Pharmacy

# Abstract: Weight Gain In Schizophrenia Patients Due To Atypical Antipsychotic Use

**Authors:** Divaz Hedya Putri, Ida Paulina Sormin, Julaeha

**Affiliations:** 1 Magister of Pharmacy, Faculty of Pharmacy, Universitas 17 Agustus 1945 Jakarta, Indonesia; 2 National Research and Innovation Agency Republic of Indonesia, Jakarta, Indonesia

**Objectives:** This study aims to evaluate the side effects of atypical antipsychotics, specifically in terms of weight gain in patients with schizophrenia after more than four weeks of use.

**Methods:** This study used retrospective design; data were collected through consecutive sampling on 51 adult inpatients in psychiatry (12 women and 39 men) who had been diagnosed with schizophrenia and had consistent daily nutrition patterns. Patients were hospitalized from April 2024 to July 2024, with the application of atypical antipsychotics in the medium term (more than four weeks). Patients’ weight was measured before starting treatment and four weeks after the use of atypical antipsychotics.

**Results and Discussion:** Patients receiving atypical antipsychotics, consisting of 12 women and 39 men aged between 18 and 40 years, showed that 70% of women and 56% of men experienced a weight increase of 1–5 kg within four weeks. The average weight of the subjects increased from 57.55 ± 10.743 kg to 59.83 ± 12.205 kg after starting treatment (*p *= 0.001). The most commonly used atypical antipsychotic was a combination of risperidone and clozapine, with a percentage of 91.25%, while clozapine alone was used by 3.75% and risperidone alone by 5%.

**Conclusions:** Administration of atypical antipsychotics for at least four weeks may lead to weight gain in patients with schizophrenia. Therefore, monitoring for metabolic side effects that may occur due to the use of atypical antipsychotics is very important.

**Keywords**: antipsychotic, metabolic, schizophrenia, weight.

# Abstract: Analysis of The Effect of Caffeine Beverage Consumption on Blood Pressure Among Young People

**Authors:** Jihan Oktavini, Diana Laila Ramatillah

**Affiliations:** Pharmacy Faculty, Universitas 17 Agustus 1945 Jakarta, Jakarta, Indonesia

**Objective:** Caffeine consumption is common among adolescents, potentially influencing blood pressure levels. This study aims to analyze the relationship between caffeine intake and blood pressure among adolescents in Jakarta, Indonesia.

**Methods:** This study utilized an analytical observational design with a cross-sectional approach. A sample of 100 adolescents aged 15–18 was selected using consecutive sampling. Inclusion criteria included moderate physical activity and no family history of hypertension. Blood pressure measurements, body mass index (BMI) calculations, and caffeine consumption evaluations were conducted using the Semi-Quantitative Food Frequency Questionnaire (SQ -FFQ). Data were analyzed using the chi-square test with a 95% confidence level.

**Result and Discussion:** Analysis revealed that 61.1% of the participants frequently consumed soft drinks, while 38.9% consumed them rarely. The chi-square test indicated no significant relationship between the consumption of soft drinks and coffee and the incidence of hypertension among adolescents. However, a trend indicated that adolescents with higher caffeine intake had slightly elevated blood pressure, which was not statistically significant.

**Results and Discussion:** The analysis showed that soft drinks 22% of participants often consume soft drinks, while 78% rarely consume them, and coffee 34% of participants consume coffee while 66% rarely consume it. The chi-square test showed a significant relationship between soft drinks and coffee consumption and the incidence of hypertension among adolescents.

**Conclusion**: The study shows a statistically significant relationship between caffeine consumption and adolescent hypertension. Further research with a larger sample size and longitudinal design is recommended to understand this relationship better.

**Keywords:** caffeine consumption, adolescents, blood pressure

# Abstract: Pathway to Better Pharmacy Practices: Development of a Consensus-Based Medication Review Service Blueprint for Community Pharmacy in Malaysia

**Authors:** Maali Mustafa, Ernieda Hatah

**Affiliations:** 1 Faculty of Pharmacy, Universiti Kebangsaan Malaysia, Kuala Lumpur, Malaysia; 2 Faculty of Pharmacy, University of Cyberjaya, Selangor, Malaysia

**Objectives:** This study aimed to develop a medication review service blueprint specifically tailored for Malaysia's unique community pharmacy setting, considering the local healthcare system and patient demographics.

**Methods:** The study utilized a mixed-methods design, combining content analysis and consensus techniques. Initially, a comparative content analysis systematically reviewed international medication review service models to identify best practices applicable to Malaysia, creating a preliminary blueprint for community pharmacy settings. A modified two-round e-Delphi method was conducted with 26 expert pharmacists from October 2022 to January 2023 to validate and refine this blueprint. Structured questionnaires collected expert opinions on key aspects such as service levels, recruitment policies, patient eligibility, and staffing requirements. Consensus was defined as achieving at least 80% agreement among the experts.

**Results and Discussion:** The content analysis identified essential components for an effective medication review service, including comprehensive medication management, patient education, and collaboration with healthcare providers. The preliminary blueprint proposed three types of services. Through the Delphi study, a refined blueprint emerged, consolidating the services into two primary levels: Medication Review-Consultancy (MR-C) and Medication Review-Clinical (MR-Clin). The refined blueprint emphasizes the need for flexible session durations, adequate staffing, and sustainable funding models, providing practical guidance for successful implementation.

**Conclusions:** The finalized blueprint offers practical guidance for implementing medication review services in Malaysian community pharmacies. Future studies should explore the adaptability of this service blueprint and develop training models to empower pharmacists.

**Keywords:** Medication review service, Delphi method, content analysis, community pharmacy, Malaysia

# Abstract: A Comparative Study On Over-The-Counter Sales Of Antibiotics And Narcotics At The Drug Sale Outlets In The Urban Areas Of Lahore, Pakistan

**Authors:** Muhammad Usman, Hammad Ahmad, Huma Rasheed

**Affiliations:** Institute of Pharmaceutical Sciences, University of Veterinary and Animal Sciences, Lahore, Pakistan

**Objectives:** The sale of controlled substances and antibiotics without a valid prescription is a serious offence perpetuating self-medication, high risk of overdosing and physical dependence in case of narcotics and antimicrobial resistance. The current study focuses on exposing the malpractices involved in selling narcotics and antibiotics without prescription in the urban areas of Lahore.

**Methods:** A mystery shopper method was adopted to evaluate the sale trend of azithromycin and bromazepam without prescription at 246 pharmacies in 10 different towns of Lahore. A three-tier scenario using a team of simulated clients visited the pharmacies and simultaneously presented false urgency for the need for both drugs. The response of pharmacy staff was documented on a coded pre-designed Performa after the visit.

**Results and Discussion:** The results of the study showed that azithromycin was freely sold at first tier in all 246 pharmacies, whereas 192(78%) pharmacies asked for a valid prescription for bromazepam and 113(45.9%) refused to sell without a prescription out of which 75% refusals were made by pharmacist and 63% were chain pharmacies. Bromazepam was dispensed with fewer checks in scruffy areas than in affluent areas (*p* < 0.001). As an outcome, 54.1% of pharmacies sold bromazepam without a prescription, and 88% were dispensed by non-pharmacists.

**Conclusions:** Chain pharmacies, effluent settings and the presence of pharmacists favoured adherence to the prescription sale of narcotics. In comparison, the unchecked dispensing of antibiotics warrants immediate action against over-the-counter sales. Stakeholders must be engaged in developing strategic tools and policy measures to control this menace.

**Keywords**: antibiotic misuse, narcotics misuse, over-the-counter sale, azithromycin, bromazepam

# Abstract: A Longitudinal Study on the Factors Associated with the Number of Research Publications among Pharmacists in the Ministry of Health Malaysia

**Authors:** Yi Huan Tan, Nor Ilham Ainaa Muhsin, Pui Lim Chan, Siti Fatimah Ayob, Nur Ain A Rashid, Noraini Nordin, Abdul Haniff Mohamad Yahaya

**Affiliations:** 1 Pharmacy Research & Development Subdivision, Pharmaceutical Services Programme, Ministry of Health Malaysia; 2 Pharmacy Professional Development Subdivision, Pharmaceutical Services Programme, Ministry of Health Malaysia

**Objectives:** Various factors can influence research publication. Understanding these factors is crucial for developing effective key performance indicators (KPIs) to enhance research productivity. This study aimed to identify the factors associated with the number of research findings published by pharmacists within the Pharmaceutical Services Programme, Ministry of Health Malaysia. The outcomes of this study aim to help researchers and policymakers adopt strategies to increase the number of research publications.

**Methods:** This exploratory study uses secondary data from 2012 to 2023. Data were gathered from databases within the Pharmaceutical Services Programme, including variables such as the number of permanent- or contract-based employment of fully registered pharmacists (FRP), pharmacy assistants, PhD holders, coursework- and research-based Master's degree holders, and training expenditure. Simple linear regression (SLR) was initially used to estimate the relationship between each factor and the pharmacy research publication numbers. Multiple linear regression (MLR) was then used to determine the combined effects of these variables. Data analysis was conducted using the statistical software IBM SPSS version 27.0.

**Results and Discussion:** The SLR analysis indicated a significant positive correlation between research publication numbers and most factors, with the number of PhD holders showing the highest coefficient of determination (R^2^) at 0.889 (*p *< 0.001; β = 1.622). Conversely, training expenditure exhibited a statistically significant negative correlation. In MLR analysis, the stepwise method was applied with all model assumptions fulfilled. The result demonstrated that only the number of permanent FRP and the PhD holders were statistically significant (*p *= 0.042 and <0.001, respectively). The model explained 93.2% of the variance in pharmacy research publication numbers [Publication = 2.434*(PhD) – 0.019*(Permanent FRP) + 141.445; R^2^ = 0.932]. These findings highlight the crucial role of highly qualified personnel in driving research productivity.

**Conclusions:** The pharmacy research publication output was significantly associated with the number of PhD holders and permanent FRP. These findings should serve as a basis for formulating future KPIs and be used as a help guide to enhance research productivity in this field.

**Keywords**: pharmacy, regression analysis, research publication, key performance indicators

# Abstract: Quantitative Evaluation of Antibiotic Use by a Community Pharmacy Using Anatomical Therapeutic Chemical/Defined Daily Dose Method: A Case Study

**Authors:** Valerie Kezia, Nadya Novelin Sinaga, Norman Dyanto, Laila R Diana

**Affiliations:** 1 Faculty of Pharmacy, 17 August 1945 University, Jakarta, Indonesia; 2 Faculty of Pharmacy, STIKes Prima, Bekasi, Indonesia

**Objective:** Antibiotics are crucial in helping the body suppress bacterial biochemical processes, especially in infections caused by bacteria. This study aimed to evaluate the use of antibiotics at a community pharmacy in West Java, Indonesia, using the Anatomical Therapeutic Chemical/Defined Daily Dose (ATC/DDD) method.

**Methods:** This research was conducted retrospectively by collecting secondary data from antibiotic sales data for outpatients at a community pharmacy in Bekasi, West Java, Indonesia, in 2020. The number of samples included in the inclusion criteria was 369, covering the period from January to December 2020, quantitatively using the ATC/DDD method. The drug utilization 90% (DU 90%) segment was used to determine the quality of drug prescribing.

**Results and Discussion:** This community pharmacy has operated since 2015 and dispensed between 700 to 1500 antibiotic prescriptions yearly. The commonly dispensed antibiotics were Amoxicillin, Azithromycin, Cotrimoxazole, Ciprofloxacin, Cefadroxil, Chloramphenicol, and Clindamycin. The antibiotic with the highest DDD/1000 outpatient visits value was Amoxicillin (292.97), followed by Cotrimoxazole (61.00), Ciprofloxacin (45.18), Cefadroxil (44.59), Azithromycin (36.89), and Chloramphenicol (0.22). Notably, Amoxicillin and Cotrimoxazole contributed most to the DU 90%, with values of 60% and 13%, respectively. Additionally, the DDD in grams for Amoxicillin (1.125 g) and Cotrimoxazole (0.235 g) were found to be within the WHO DDD standards for Amoxicillin (1.5 g) and Cotrimoxazole (0.4 g).

**Conclusions:** This case study evaluated data from a single community pharmacy to provide preliminary insight into the rational use of antibiotics in the region. Future studies may employ larger sample sizes and data from more community pharmacies in Indonesia to provide a better understanding of the rational use of antibiotics in the nation.

**Keywords:** ATC/DDD, Community Pharmacy, Antibiotic Use

# Abstract: Practice of Medication Storage and Disposal among Patients with Chronic Medications in Sibu Hospital

**Authors:** Kong Mei Chieng, Wong Siaw Ying, Ivy Siew Wen Juan, Lily Tiong

**Affiliations:** Department of Pharmacy, Sibu Hospital, Sarawak

**Objectives:** Inappropriate storage of home medications is a global issue that may affect the effectiveness and stability of medications, compromising the ability to treat chronic diseases effectively. We explored the practice of medication storage and disposal among patients with chronic medications in Sibu Hospital.

**Methods:** A cross-sectional study was conducted at Sibu Hospital, Sarawak, from December 2021 to February 2022 among patients aged ≥18 with at least one chronic disease and medication. Patients who were diagnosed with active psychiatric conditions and unable to give informed consent were excluded. The convenience sampling method was applied by interviewing patients at inpatient and outpatient pharmacies. Data was collected via face-to-face interviews using validated questionnaires formulated based on MyHEALTH KKM Safe Storage Protocol, Meditips Kenali Ubat Anda and the literature “Development and Validation of Medication Storage and Disposal Questionnaire”.

**Results and Discussion:** A total of 444 respondents were recruited, with a response rate of 96.5%. The majority of the respondents were Iban (53.6%), followed by Chinese (30.6%), Malay (14.2%) and other ethnic (1.6%), with a mean age of 54.34 years old. The findings revealed that 77.3% of the respondents practised improper storage (*n* = 343) with an average score of 86.2%, whereas 97.7% poorly managed medication disposal (*n* = 434) with an average score of 11.1%. Multiple logistic regression was carried out to assess the effects of gender, age, ethnicity, education level and income on the likelihood of proper medication storage and disposal. The overall model was statistically significant compared to the null model (X2(11) = 28.78, *p *= 0.002), explained 9.5% of the storage variation and correctly predicted 77.7% of cases. It found that respondents with a mean age of 54.34 years old without formal education had inadequate knowledge of storage (*p *= 0.004). The findings also suggested that Iban respondents with income ≤RM3000 and who received primary education had poorer knowledge of storage (*p *= 0.003). There was no significant association between sociodemographic factors and disposal.

**Conclusions:** From the results, we concluded that respondents were less aware of handling medication at home. Pharmacists should play an active role in emphasising appropriate medication storage and disposal through awareness campaigns.

**Keywords:** chronic medications, storage, disposal

# Abstract: Prerequisite of Conducting Mentoring Program and Protected Research Time for the Impact of Pharmacists’ Research in Johor Public Healthcare Facilities

**Authors:** Zahrina A.K., Ahmad Yuzaily B., Mohamad Ammar Z., Diana Yap F.S., Lee S.L., Shantasaroopini S.M., Tan A.Y., Lim S.H., Ng M.W., Hiswani M., Nur Hidayah K., Lee C.Y.

**Affiliations:** 1 Pharmaceutical Services Division, Johor State Health Department; 2 Pharmacy Department, Hospital Enche Besar Hajjah Khalsom, Kluang; 3 Pharmacy Department, Hospital Sultanah Aminah, Johor Bahru; 4 Pharmacy Department, Hospital Pakar Sultanah Fatimah, Muar; 5 Pharmacy Department, Hospital Sultan Ismail, Johor Bahru; 6 Pharmacy Department, Hospital Segamat; 7 Pharmacy Department, Hospital Pontian; 8 Pharmacy Unit, Muar District Health Office; 9 Pharmacy Unit, Johor Bahru District Health Office

**Objectives:** This study aims to provide baseline data for enhancing the research capacity of pharmacists and its impact on pharmaceutical services. It identifies the perceived research impact regarding system and personal expectations and the suitability of incentives.

**Methods:** A cross-sectional survey was conducted among registered pharmacists employed in 23 public facilities in Johor, Malaysia, for over a year. Consented respondents completed a validated online questionnaire, which was analyzed in two domains: self-perceived confidence scores (5-point Likert scale) and research impact (Agree, Disagree, Non-applicable).

**Results:** Of the 378 respondents, 339 (89.68%) reported management support, but only 78 (20.63%) received protected research time (PRT). Although 312 (82.54%) had participated in various research roles, 157 (41.53%) lacked mentorship, with the most common form being peer discussion led by principal investigators (120, 31.75%). The principal investigator was perceived as the most confident team member, with a mean confidence score (MCS) of 66.12 ± 16.87 compared to 61.57 ± 16.73 for all respondents. A one-way ANOVA revealed significant differences in MCS among groups (F(3, 374)  =  3.83, *p *= 0.010). Tukey's HSD Test indicated significant differences in MCS between the principal investigator and both co-investigators (59.46 ± 16.46, *p *= 0.020, 95% CI =  [0.75, 12.56]) and other collaborators (59.26 ± 15.94, *p *= 0.021, 95% CI =  [0.73, 13.00]). Impact ratings showed optimism for improved service quality (181, 47.88%) and increased quality awareness (167, 44.18%). Additionally, 121 (32.01%) felt their research helped secure future resources, while 141 (37.30%) linked it to capacity building. Despite system deficiencies, team member satisfaction, personal goal achievement, and personal growth were notable (42.33%, 41.53%, 40.74%).

**Conclusions:** There is a notable lack of system incentives among the less optimistic group, contrasted with a self-driven effort in the personal achievement group. For research teams to produce higher-quality projects, practical measures such as mentorship and PRT must be instituted.

**Keywords:** Mentorship, Confidence, Pharmaceutical Services, Capacity Building, Research Impact

# Abstract: Prevalence and Outcome of Transcribing Errors in Healthcare Facilities in Malaysia

**Authors:** Norazida Ab Rahman, Ee Vien Low, Fateha Kamaruddin, Asilah Che Ayub, Sue Chin Chong, Norafidah Idris

**Affiliations:** 1 Institute for Clinical Research, National Institutes of Health, Ministry of Health Malaysia; 2 Institute for Health System Research, National Institutes of Health, Ministry of Health Malaysia; 3 Pharmacy Practice & Development Division, Pharmaceutical Services Programme, Ministry of Health Malaysia

**Objectives:** This study aims to determine the prevalence, characteristics, and outcomes of medication errors (MEs) due to transcribing errors reported via Malaysia's national reporting system.

**Methods:** We retrospectively analysed reports from the Medication Error Reporting System (MERS) Malaysia. All reports of medication errors due to transcribing errors in healthcare facilities between 2018 and 2022 were included for analysis. Descriptive statistics were applied.

**Results and Discussion:** 12,123 MEs related to transcribing errors were reported. Almost all (99.9%) of the MEs were reported from the public sector. More than half of the medication errors occurred in health clinics (52.8%), and 47.2% occurred in hospital settings. Out of the total report received, 540 (4.5%) incidents were identified as errors that reached the patients. Most of the incidents where errors reached patients occurred in the pharmacy (*n* = 515, 95.4%), and more than half of the patients took the incorrect medication (*n* = 301, 55.7%). Most errors did not harm the patients (*n* = 431, 79.8%). The leading causes of the medication error that reached patients were identified as task and technology-related issues (*n* = 386, 71.5%), followed by work and environment (*n* = 290, 53.7%) and staff factors (*n* = 271, 50.2%).

**Conclusions:** More effort is needed to promote MERS in the private and public sectors. Simultaneously, preventive measures need to be implemented to reduce ME as it is a preventable error that can potentially harm patients.

**Keywords**: medication error, transcribing error, medication-related, medication error reporting system

# Abstract: Unravelling the Profile of Bioactive Compounds and Its Potential Medicinal Properties from the ***Aruda*** Plant (***Ruta graveolens*** L.) in Timor-Leste: From Traditional Knowledge to In-Silico Bioprospecting

**Authors:** Cipriana Gusmao, Diana Laila Ramatillah, Ali Budhi Kusuma, Adhityo Wicaksono, Muhammad Ade Putra

**Affiliations:** 1 Department of Pharmacy, Dili Institute of Health, Dili, Timor-Leste; 2 Department of Pharmacy, Pharmacy Faculty University of 17 Agustus, Jakarta, Indonesia; 3 ABS Project Management Unit – UNEP, National Directorate for Biodiversity, Democratic Republic of Timor-Leste, Dili, Timor-Leste; 4 Department of Biotechnology, Faculty of Life Sciences and Technology, Sumbawa, Indonesia; 5 Division of Biotechnology, Genbinesia Foundation, Gresik, Indonesia; 6 Department of Product Development, PT. Archipelago Biotechnology Indonesia, Sumbawa, Indonesia

**Objectives:** This study aims to investigate the profile of bioactive compounds from the *Aruda* medicinal plant (*Ruta graveolens* L.), one of the most important medicinal plants in Timor-Leste. Furthermore, we provide an in-silico bioprospecting study to reveal the relationship between the potential bioactivity of each identified compound and its associated traditional knowledge following the molecular docking experiment.

**Methods:** The traditional knowledge (TK) data related to the medicinal properties of the Timorese *Aruda* plant was recorded through in-depth consultation with the indigenous community in Larisula Village, Baucau Municipality, Timor-Leste. The profile of bioactive metabolites from the crude ethanol extract of the Timorese *Aruda* plant was determined using the Liquid Chromatography – Mass Spectrophotometry/Mass spectrophotometry (LC-MS/MS) platform. The resultant peak data were then revealed using Masslynk software and further identified using PubChem Database. Lastly, the potential bioactivities of the most promising compounds were tested against the targeted ligands following the molecular docking procedure.

**Results and Discussion:** The LC-MS/MS analysis revealed that the Aruda (Ruta graveolens L.) ethanolic extract contains over 15 bioactive compounds from various chemical groups, including phenolic compounds and antioxidants. Additionally, molecular docking analysis of some representative compounds showed a positive correlation with the medicinal properties documented by the Indigenous community in Larisula Village, such as curing infections, providing analgesic effects, and exhibiting antipyretic properties.

**Conclusions:** This study provides valuable insights into the positive impact of incorporating scientific investigation to provide further evidence of beneficial claims obtained from the local's traditional knowledge regarding medicinal plants in Timor-Leste.

**Keywords**: Aruda (*Ruta graveolens* L.), Timor-Leste, Medicinal Plant, LC-MS/MS, Molecular Docking

# Abstract: Prices, availability and affordability of essential antiviral medicines in Pakistan, a cross-sectional analysis warranting action

**Authors:** Huma Rasheed, Umaira Israr

**Affiliations:** Institute of Pharmaceutical Sciences, University of Veterinary and Animal Sciences, Lahore

**Objectives:** The aim of the study was to evaluate prices, availability and affordability of essential antiviral medicines in Lahore, Pakistan as the country is challenged with exceeding burden of hepatitis and AIDs.

**Methods:** A total of 31 registered essential antivirals, including 11 antiretrovirals, were surveyed across five public and 12 private sector pharmacies, as well as three public sector sites from a vertical program and a central procurement centre. Tender prices for originator brands (OB) and the lowest-priced generic (LPG) in the public sector were collected, along with details of fast-moving brands (FMB) and available brands in the private sector. The study determined international reference price comparisons, median price ratios (MPR), percentage availability, and affordability.

**Results and Discussion:** The availability of 15 anti-hepatitis medicines was found to be “very low” with average of 29.33% in public sector and 26.66% in private sector (p*-value  = 0.0243). Mean availability for OBs was 3.2% and 9.6%, for LPGs; it was 32.25 and 19.35% for public and private sector, respectively. The availability of FMB brands of antivirals in private sector was 35.48%. The highest median price for LPG, FMB and OB was 2.1$, 3.16$ and 8.8$, respectively. Seven antivirals were found “unaffordable” and the patient for cytomegalovirus, Hepatitis C and Herpes Simplex would need wages for 162 days (396.9$), 12.94 days (31.703$) and 21.80 days (53.41$), respectively to afford the therapy.

**Conclusion:** The availability of essential antivirals is very low with difference of prices in public and private sector and compromised affordability, warranting induction of low- priced generics and improved competition.

**Keywords:** access to essential medicines, antivirals, antiretrovirals, availability, affordability, prices, Low and middle income countries, generic medicines

# Abstract: Diabetic Peripheral Neuropathy Treatment and Vitamin B Supplementation in Hospital Pakar Sultanah Fatimah, Johor

**Authors:** Ng Kar Xin, Zakiah A Rahman, Muhamad Aizuddin Roszali, Soh Ke Ying

**Affiliations:** Pharmacy Department, Hospital Pakar Sultanah Fatimah, Johor

**Objectives:** This study investigates the outcome and safety of DPN treatments.

**Methods:** An observational cross-sectional study was conducted in the outpatient pharmacy of Hospital Pakar Sultanah Fatimah (HPSF). The investigator interviewed patients≥18 years old who are on DPN treatment. The details regarding DPN treatment were recorded. The improvement of DPN symptoms assessed treatment outcome. The safety of the treatment was assessed by documenting the adverse effects of taking DPN medications.

**Results and Discussion:** Among the 102 patients in the study, 70.6% reported improvement in DPN symptoms after starting treatment. The most used DPN treatment was the Vitamin B1, B6, and B12 combination tablet (52.6%), followed by Vitamin B12 tablet (28.4%), Gabapentin capsule (14.7%) and Tramadol capsule (3.4%). Most patients received DPN treatment as monotherapy (84.3%), while 15.7% were given combination therapy to treat DPN. The most used combination therapy is the combination of vitamin B supplements with other DPN agents. There is no significant difference between monotherapy and combination therapy with improvements to DPN symptoms (*P*-value  =  0.537). Comparing the two types of Vitamin B supplements used in the treatment of DPN, there is no significant difference between taking Vitamin B1, B6, and B12 tablets and taking Vitamin B12 tablets regarding DPN symptoms improvement (*P*-value  =  0.467). Regarding safety, 75% of patients on Tramadol experienced adverse effects compared to 41.2% of patients on Gabapentin and 3.2% of patients on Vitamin B supplements.

**Conclusions:** Treatment of DPN should be individualized. The use of combination therapy is preferred if patients’ symptoms do not improve on monotherapy. Vitamin B supplements caused the lowest adverse effects compared to other agents. Healthcare professionals should always follow up with patients on DPN treatments to review symptom improvement and adverse effects experienced by patients.

**Keywords**: Diabetic peripheral neuropathy (DPN), pain, treatment, outcome, adverse effects

# Abstract: Differential Serum Metabolomic Profile in Preterm Infants Receiving Parenteral Amino Acid Solution Enriched with N-Acetylated-Tyrosine versus Deacetylated-Tyrosine

**Authors:** Sing Kiat Chuo, Jen Kit Tan, Wye Leng Elaine Hooi, Seok Chiong Chee, Fook Choe Cheah

**Affiliations:** 1 Master of Medical Science (candidate); Faculty of Medicine, Universiti Kebangsaan Malaysia; 2 Department of Pharmacy, Selayang Hospital, Universiti Kebangsaan Malaysia; 3 Department of Biochemistry, Faculty of Medicine, Universiti Kebangsaan Malaysia; 4 Department of Paediatrics, Selayang Hospital; 5 Faculty of Health and Medical Sciences, Taylor’s University; 6 Department of Paediatrics, Faculty of Medicine, Universiti Kebangsaan Malaysia

**Objectives:** This study compares the impact of using N-acetylated-tyrosine enriched versus deacetylated tyrosine parenteral amino acid (AA) solution in neonatal parenteral nutrition (PN).

**Methods:** Serum samples from preterm infants admitted to two neonatal units (HM, HS) in the first week of life were analysed using an untargeted metabolomic approach with ultrahigh-performance liquid chromatography-mass spectrometry. Clinical data was recorded until discharge.

**Results and Discussion:** Sixty-four preterm infants were purposively recruited (HM, *n* = 37, HS, *n* = 27). Principal component analysis did not reveal a clear separation in metabolite clusters, indicating more similarities than differences between both groups. Of the 147 metabolites identified, 19 were differentially expressed (*p *< 0.05). Fold-change analysis showed at least a 660-fold increase in N-acetyl-tyrosine and tyrosine. Pathway analysis suggested the phenylalanine, tyrosine and tryptophan biosynthesis and arginine biosynthesis were significantly altered in the infants receiving the tyrosine-enriched AA solution (*p *< 0.05, I > 0.1). HM infants had a shorter duration of PN [median, (IQR) = 6(6) vs 12(6) days]) Furthermore, hospitalisation [median, (IQR) =  42(32) vs 78(73) days] and higher growth velocity from the age of birth-weight regained to a postmenstrual age of 36 weeks gestation [median (IQR) = 16.10 (5.2) vs 13.80 (3.9) g/kg/day)].

**Conclusions:** Our findings provide preliminary insights into the use of metabolomics in personalising preterm nutrition. There is a pivotal need to review the amino acid component used in neonatal PN and the clinical impact it delivers.

**Keywords**: metabolomic; parenteral nutrition; tyrosine; amino acid; infant

# Abstract: Evaluation Of Drug Use In Patients With Coronary Heart Disease Who Have Received The Covid-19 Booster

**Authors:** Lia Susika, Diana Laila Ramatillah, Dr. Mohamed Elnaem

**Affiliations:** 1 Pharmacy Faculty, Universitas 17 Agustus 1945 Jakarta; 2 Ulster University

**Objectives:** This study aims to evaluate the patterns of medication use and demographic characteristics of coronary heart disease patients who have received the COVID-19 Booster 1 vaccine. Specifically, it seeks to identify common comorbidities, prevalent medications, and any potential impacts of booster vaccination on the management of these patients.

**Methods:** A prospective and retrospective cohort analytic observational design was employed. Data was collected at a hospital in Jakarta between January 2022 and May 2024. The study population included patients diagnosed with coronary heart disease who had received the COVID-19 Booster 1 vaccine. Convenience sampling was utilized to select participants. Data on patient demographics, comorbidities, and medication use were gathered from medical records.

**Results and Discussion:** A total of 110 coronary heart disease patients who received the COVID-19 Booster 1 vaccine were included in the study. The majority were male (52.7%) and aged between 51 and 70 (58.2%). Hypertension is the most common comorbidity affecting 58,2% of the patients. The medications most frequently used included antihypertensives (potassium-saving diuretics) in 41.8% of patients, atorvastatin (anti-dyslipidemia) in 50.9%, metformin (antidiabetic) in 15.5%, clopidogrel (antithrombotic) in 62.7%, and nitroglycerin (nitrate) in 60.0%, along with other additional medications as needed for individual patient management. The demographic analysis indicates a higher prevalence of coronary heart disease among males and older adults.The significant use of antihypertensives medications underscores the importance of managing coronary heart disease with comorbid hypertension in this population. The frequent use of clopidogrel highlights the emphasis on preventing thrombotic events in these patients.

**Conclusions:** The study successfully identified the demographic characteristics and medication use patterns among coronary heart disease patients who received the COVID-19 Booster 1 vaccine. The findings reveal a predominance of males aged 51–70, with hypertension as a common comorbidity. Effective management involves the extensive use of antihypertensives, atorvastatin, and clopidogrel. These insights can guide healthcare providers in optimizing therapeutic strategies and resource allocation for coronary heart disease patients post-COVID-19 booster vaccination.

**Keywords:** Coronary Heart Disease, Medication Use, COVID-19 Booster Vaccine, Hypertension, Sociodemography

# Abstract: Can ChatGPT replace the role of drug information pharmacists? Evaluating ChatGPT's accuracy and consistency in drug information queries

**Authors:** Boon Phiaw Kho, Kuek Hui Shi Laura, Wei Wern Khor, Ma Yii Chien Evelyn

**Affiliations:** Department of Pharmacy, Sarawak General Hospital, Sarawak, Malaysia

**Objectives:** This study investigates the accuracy and reproducibility of answers generated by ChatGPT in response to a selection of drug information queries.

**Methods:** A total of 180 query records from nine domains (20 queries each) were retrospectively retrieved from the database of a hospital's pharmacy drug information unit. The queries were entered into two ChatGPT-4 accounts: one was given a specific persona (role with detailed instructions to optimise the response), and the other was not. The accuracy of the answers was evaluated against the initial answers provided by pharmacists, which were guided by professional literature and reverified by study investigators. For reproducibility analysis, the queries were reentered into the account with the specific persona at least three weeks later, with half answered using the GPT-4 model and the other half using the GPT-4-mini model.

**Results and Discussion:** Providing ChatGPT with a specific persona marginally increased the answers’ accuracy (86.7%, *n* = 156/180 vs 81.1%, *n* = 146/180). For reproducibility, the GPT-4 model was superior to the GPT4-mini model, with consistent answers generated for 85.6% (*n* = 77/90) versus 58.9% (*n *= 53/90) of queries. ChatGPT performed best in answering queries concerning adverse drug reactions and toxicity and worst in general paediatric doses. It provided detailed reasoning for the answers, which can assist pharmacists in their decision-making process. However, it is not error-free, and hallucinations were detected. Some responses also could not be found in the reference list provided.

**Conclusions:** Understanding the capabilities and limitations of ChatGPT is important to define the boundaries for safe use. The current version cannot fully and safely replace drug information pharmacists. Nonetheless, it can be a useful companion for pharmacists to sense-check an answer. To optimise accuracy, utilising the GPT-4 model and providing it with a persona is recommended.

**Keywords**: ChatGPT, Drug Information, Accuracy, Reproducibility, Pharmacy

# Abstract: Access and Affordability of Essential Medicines for the Management of Cardiometabolic Diseases in Lahore, Pakistan

**Authors:** Huma Rasheed, Sapna Zafar Gill

**Affiliations:** Institute of Pharmaceutical Sciences, University of Veterinary and Animal Sciences, Lahore, Pakistan

**Objectives:** This study evaluated the availability, affordability, registration status, and brand premium of essential medicines for cardiometabolic diseases, including cardiovascular conditions and diabetes, in Lahore.

**Methods:** A cross-sectional survey was conducted in Lahore's public and private retail pharmacies. Data on the availability, registration status, and prices of essential medicines for cardiovascular diseases and diabetes, including originator brands and lowest-priced generics, were collected on-site. Affordability was measured by calculating the cost of a standard treatment regimen with the median daily wage, determining how many days’ wages would be required to afford the treatment. The brand premium was analyzed by comparing the cost differences between originator brands and lowest-priced generics.

**Results and Discussion:** The study included 78 essential medicines, with 63 (80.8%) registered for cardiovascular diseases and 12 (80%) registered for diabetes. On-the-spot availability showed that ten antidiabetic medicines were accessible. The minimum price for cardiovascular medicines ranged from 1 PKR (amlodipine 5 mg tablet) to 6375 PKR (streptokinase powder for injection 1.5 MIU), while for antidiabetic medicines, prices ranged from 1 PKR (metformin 500 mg tablet) to 7075 PKR (long-acting insulin 100 IU/ml in a 3 mL vial). The brand premium was calculated for six antidiabetic and 32 cardiovascular medicines where originator brands were available. Affordability analysis indicated that the cost of treatment for 56% of cardiovascular and 50% of antidiabetic medicines required more than one day's wage, making them unaffordable for many patients.

**Conclusions:** The study highlights that while most essential medicines for cardiometabolic diseases are registered and available in Lahore, their affordability remains a significant challenge. A considerable portion of the treatment costs exceeds the daily wage of patients, underscoring the need for policies to optimize pricing and improve access to these vital medicines.

**Keywords:** Access to medicines, Essential medicines, Cardiovascular drugs, Antidiabetic drugs

# Abstract: Identification Of Challenges In Open Tender Medicines Procurement By Public Hospitals of Punjab

**Authors:** Huma Rasheed, Kashif Rehman

**Affiliations:** Institute of Pharmaceutical Sciences, University of Veterinary and Animal Sciences, Lahore, Pakistan

**Objective:** This study aims to analyze the disparities in the annual procurement of essential medicines in public hospitals across three Punjab districts, Pakistan, from 2019 to 2022.

**Methods:** A quantitative cross-sectional descriptive study was adopted to analyze tender participation and quoted prices across districts A, B, and C over three years (2019–2022), as retrieved from the official website.

**Results and Discussion:** The number of tendered items is 24 to 193 per year, with the most being part of the essential medicines lists. The maximum tender participation was in the year 2020–2021 in district B (85%), whereas the highest zero bidding was 58% in district A for 2019–2020. In an overall inter-district comparison, district A received a higher rate than districts B and C, with increased medicines prices of up to 209–575%. Four medicines never received any bid in the nine tenders. In comparison, ten medicines did not receive any bid in two years in any of the districts. The number of monopoly items in year one was 49, 51, and 62; for year two, 40, 29, and 31; and in year three, 39, 28, and 58 for districts A, B and C, respectively. Only six medicinal products had maximum bidding competition with more than five bids.

**Conclusion:** The study highlights the disparities in essential medicine procurement in public hospitals of Punjab, which would adversely affect access to medicines by the public. The varied bidding participation, lack of competition, and fluctuating price trends point to economic and administrative challenges.

**Keywords**: access to medicines, essential medicines**,** open-**t**ender, pharmaceutical market, life-saving medicines

# Abstract: Factors Non-adherence Related to Enhance Medication Adherence Among Hypertension Patients Worldwide: A Systematic Review

**Authors:** Vania Jessica Ongkers, Diana Laila Ramatillah, Paulina Barbalina Pelasula

**Affiliations:** Department of Pharmacy, Universitas 17 Agustus 1945 Jakarta, Jakarta, Indonesia

**Objectives:** Medication non-adherence is a significant challenge in managing hypertension, leading to poor health outcomes and increased healthcare costs. Understanding the factors contributing to non-adherence and identifying effective strategies to enhance adherence is crucial for improving patient outcomes. This study aimed to identify factors contributing to medication non-adherence and strategies to enhance medication adherence among hypertension patients globally.

**Methods:** This systematic review used databases such as Google Scholar, Scopus, and PubMed, leveraging generative AI tools like Open Knowledge Maps to identify relevant studies from 2019 to 2024. The PICO method (Population/Problem, Intervention, Comparison, and Outcome) was used to frame the research questions. Purposive sampling was employed to select studies that met the inclusion criteria. Data on patients’ sociodemographics and factors related to medication non-adherence were extracted. To minimise bias, literature selection and data extraction were performed using automated and manual methods, including COVIDENCE AI tools. The PRISMA (Preferred Reporting Items for Systematic Reviews and Meta-Analyses) guidelines were followed to ensure a systematic approach.

**Results and Discussion:** From the 240 studies screened, ten met the inclusion criteria and were included in the review. The analysis revealed that five key dimensions influence medication non-adherence among hypertension patients according to the WHO framework: 1) Social and economic factors (e.g., financial constraints, social demands), 2) Healthcare team and system-related factors (e.g., healthcare access, provider-patient communication), 3) Condition-related factors (e.g., comorbidities, severity of hypertension), 4) Therapy-related factors (e.g., side effects, complexity of medication regimen), and 5) Patient-related factors (e.g., health literacy, motivation). Strategies to address these factors were identified, including patient education, simplified medication regimens, and improved healthcare-provider communication. These findings highlight the multifaceted nature of medication non-adherence in hypertension patients. Addressing the identified factors through targeted interventions can enhance medication adherence and improve clinical outcomes.

**Conclusions:** Medication non-adherence in hypertension patients is influenced by multiple factors across five dimensions. Targeted strategies addressing these factors are crucial for improving adherence and patient outcomes.

**Keywords:** Hypertension, Medication adherence, Non-adherence, Systematic review, Patient education, Healthcare access

# Abstract: Evaluation of Market Authorization of Essential Medicines Related to Mental Health and Central Nervous System Disorders

**Authors:** Huma Rasheed, Ahmad Raza

**Affiliations:** Institute of Pharmaceutical Sciences, University of Veterinary and Animal Sciences, Lahore, Pakistan

**Objectives:** This study documents the registration status of medicines for mental health and central nervous system (CNS) disorders listed in the WHO's 2021 Essential Medicines List through comprehensive document analysis.

**Methods:** The National Essential Medicines List of Pakistan 2018 was consolidated with the 22nd WHO Model List of Essential Medicines 2021. The categories examined included anticonvulsants/antiepileptics (Category 5), anti-migraine medicines (Category 7), anti-parkinsonism medicines (Category 9), and medicines for mental and behavioural disorders (Category 24). The 34 generic medicines were expanded to 106 medicinal products. Registration status and the number of brands were verified using three publicly available drug information sources and a validation process.

**Results and Discussion:** The analysis revealed that Category 5 had 20 out of 39 medicines (51.28%) unregistered. Category 7 had 2 out of 11 medicines (18.18%) unregistered. Category 9 had 3 out of 5 medicines (60%) unregistered. Category 24 had 25 out of 51 medicines (49.01%) unregistered. Overall, out of 106 medicines, 56 were registered, with 26 (46.43%) of these having ten or more brands listed in the sources. Eleven products (19.64%) were registered with only one brand. A total of 50 medicines (47.17%) were not registered. Further analysis indicated that six products (12%) were registered with slightly different specifications, and four products (8%) could be made available through compounding in community pharmacies.

**Conclusions:** The lack of market authorization for essential mental health and CNS disorder medicines significantly limits access and treatment options. Immediate policy interventions are necessary to address these gaps.

**Keywords:** Mental health medicines, CNS disorders, Essential medicines, Market authorization, Registration status

# Abstract: Exploring The Prevalence, Influencing Factors and Coping Mechanisms of Mental Burnout Among Healthcare Professionals in Hospital Raja Permaisuri Bainun (HRPB), Perak

**Authors:** Nuratikah Adillah Rezuan, Aina Amanina Abdul Jalil, Zakiah Mohd Noordin, Seow Lay Jing

**Affiliations:** 1 Faculty of Pharmacy and Health Sciences, Royal College of Medicine Perak, Universiti Kuala Lumpur (RCMP UniKL), Ipoh, Perak, Malaysia; 2 Department of Pharmacy Practice and Clinical Pharmacy, Faculty of Pharmacy, Universiti Teknologi MARA Selangor Branch, Puncak Alam Campus, Bandar Puncak Alam, Malaysia

**Objectives:** This study examines the prevalence, associated factors and coping strategies related to burnout among healthcare professionals in Hospital Raja Permaisuri Bainun (HRPB), Perak.

**Methods:** This study used an analytic observational method with a cross-sectional design, collecting data via online and printed questionnaires. Healthcare professionals from HRPB participated through non-probability convenience and snowball sampling. Descriptive statistics analysed the prevalence and coping mechanisms of mental and psychological burnout, while Pearson's Chi-Square and inferential statistics identified associations between burnout and demographic factors such as age, gender, and education (*p*-value < 0.05).

**Results and Discussion:** The study revealed a moderate prevalence of burnout (54.7%) among healthcare professionals at HRPB. Most participants experienced burnout related to personal and work factors (81.9%) and patient-related factors (85.5%). Significant associations were found between burnout and the respondents’ age (*p* < 0.001) as well as professional status (*p* = 0.013). Among the coping mechanisms, religion was the most commonly utilised by healthcare professionals at HRPB.

**Conclusions:** Burnout among HRPB healthcare professionals is moderately prevalent and significantly influenced by age and professional status factors. Religion is a primary coping mechanism for many. These findings highlight the need for government institutions to create targeted training programs and interventions to reduce burnout and improve healthcare professionals’ well-being and job satisfaction.

**Keywords**: burnout, healthcare professionals, coping mechanisms, mental health

# Abstract: Impact of Aspirin and Clopidogrel on Therapeutic Outcomes in Stroke Patients Vaccinated for COVID-19: A Cross-Sectional Study

**Authors:** Bertha Alfreda Amoret Zega, Diana Laila Ramatillah

**Affiliations:** Pharmacy Faculty, Universitas 17 Agustus 1945 Jakarta, Jakarta, Indonesia

**Introduction:** Stroke is a leading cause of morbidity and mortality worldwide. The management of stroke patients has become even more complex in the context of the COVID-19 pandemic, particularly concerning the effects of vaccinations and concurrent medications such as aspirin and clopidogrel. Understanding how these medications influence therapeutic outcomes in vaccinated stroke patients is crucial for optimizing treatment protocols.

**Objectives:** This study aimed to evaluate the impact of aspirin on the therapeutic outcomes of stroke patients vaccinated for COVID-19 compared to the effects of clopidogrel.

**Methods:** This prospective cross-sectional study enrolled adult stroke patients vaccinated for COVID-19 between January 2019 and December 2023. Patients were included if they had a confirmed diagnosis of stroke, had received at least one dose of the COVID-19 vaccine, and were prescribed either aspirin or clopidogrel. Convenience sampling was used to recruit participants, who were asked to complete questionnaires distributed in both physical form and via Google Forms. The questionnaires collected data on patient demographics, medical history, medication use, and therapy outcomes. Outcomes were categorized as improved, stable, or worsened based on clinical assessments. Statistical analysis was performed using chi-square tests to determine the significance of associations between medication use, gender, and therapy outcomes. A *p*-value of less than 0.05 was considered statistically significant.

**Results and Discussion:** Among the 200 patients, 68 (70.1%) showed improvement with clopidogrel 75 mg, 49 (47.6%) had stable outcomes with aspirin 80 mg, and 3 (2.9%) worsened with aspirin 80 mg. Gender-specific outcomes showed that 62 (52.1%) females improved, 40 (52.6%) males had stable outcomes, and all five patients with worsened outcomes were female. A significant association was found between drug use and outcomes (*p *= 0.007), while the association between gender and outcomes was not significant (*p *= 0.083).

**Conclusions:** The use of aspirin and clopidogrel is associated with therapeutic outcomes in stroke patients after COVID-19 vaccination. These findings highlight the importance of personalized treatment plans based on medication type.

**Keywords:** Aspirin, Clopidogrel, Stroke, COVID-19, Therapeutic outcomes, Medication adherence

# Abstract: Divulging the Knowledge, Attitudes and Perception on Halal Pharmaceuticals among Healthcare Practitioners: A Convergent Design

**Authors:** Marianne I. Meriales, Joan S. Nacua, Saleha Sadeeqa

**Affiliations:** 1 Department of Pharmacy, University of Southern Mindanao, Philippines; 2 Department of Pharmacy, University of the Immaculate Conception, Philippines; 3 Department of Pharmacy Practice, Lahore College for Women University, Pakistan

**Objectives:** This study aims to divulge the knowledge, attitudes and perceptions of healthcare practitioners – physicians, nurses and pharmacists and their insights on halal pharmaceuticals.

**Methods:** This study utilized a mixed-method convergent design. In the quantitative phase, a survey was administered to healthcare practitioners to assess their knowledge, attitudes, and perceptions of halal pharmaceuticals, with statistical analyses employed to identify differences in these areas. Simultaneously, qualitative data were collected to gather in-depth insights from healthcare practitioners, and thematic analysis was used to generate relevant themes. The integration of quantitative and qualitative data was presented through a joint display, highlighting areas of convergence and divergence.

**Results and Discussion:** The test of differences in the level of knowledge among healthcare practitioners showed significant variations based on area of practice, religion, and profession. In terms of attitudes, significant differences were observed concerning religion and profession. Additionally, differences in perceptions were identified based on age and length of service. The joint display of salient quantitative and qualitative findings reveals that healthcare practitioners’ knowledge is often based on appearances rather than reality. In their attitudes, healthcare practitioners highly value cultural sensitivity for maintaining integrity and significantly consider the availability and cost-efficiency of alternatives.

**Conclusions:** This study offers valuable insights into healthcare practitioners’ knowledge, attitudes, and perceptions regarding halal pharmaceuticals. Practitioners place high importance on the disclosure of animal-derived ingredients, the availability of alternatives, and transparent labelling. Additionally, they strive to satisfy subjective constraints, such as respect for beliefs, social outcomes, and practical utilities.

**Keywords:** Mixed method, halal pharmaceuticals, KAP, healthcare practitioners, Convergent design

# Abstract: Descriptive Study of Vitamin D Deficiency and Associated Factors Among Adults in Ipoh, Malaysia

**Authors:** Rara Merinda Puspitasari, Hazatul Humaira Hamdi, Nor Miza Izzati Mohd Basri, Zaswiza Mohamad Noor, Mohamad Adli Mohamad Naser

**Affiliations:** Faculty of Pharmacy and Health Sciences, Royal College of Medicine Perak, University Kuala Lumpur, Perak, Malaysia

**Objectives:** Vitamin D is crucial for various physiological processes, including bone health and immune function. Despite its importance, vitamin D deficiency (VDD) is prevalent globally. This study investigates vitamin D levels and factors associated with VDD among study participants.

**Methods:** This study involved 57 healthy adults. Participants completed questionnaires covering anthropometric data, habits and lifestyle, sun exposure, dietary intake of vitamin D, and skin type. Blood tests measured their vitamin D levels. Data analysis was conducted using descriptive statistics in SPSS software.

**Results and Discussion:** Our study found that 87.7% of participants had VDD, and 8.8% had insufficient levels. Significant predictive factors for vitamin D levels identified in previous studies include age, gender, BMI, sun exposure, and skin type. Participants under 40 had a higher prevalence of VDD, consistent with our finding that 75.4% were young adults. Over half of those with inadequate vitamin D were overweight/obese, had less than 30 minutes of daily sun exposure, and were exposed only to their hands and face. Inadequate levels were also prominent among females, particularly Muslim women who wore covered clothing. Despite these risk factors, most participants do not take vitamin D supplements frequently.

**Conclusions:** Our findings underscore the high prevalence of VDD among participants. The results highlight the need for targeted interventions, particularly for younger adults, individuals with higher BMI, and women who wear covered clothing. Personalised strategies to increase sun exposure and dietary vitamin D intake are essential to address this widespread deficiency.

**Keywords**: vitamin D, vitamin D deficiency, VDD

# Abstract: Comparative Analysis of Gentamicin Dosing Strategies in Adult Orthopedic Patients

**Authors:** Luan Mee Lu, Shirlie Chai, Sarah Heng Ying Lau

**Affiliations:** 1 Pharmacy Department, Miri Hospital, Ministry of Health Malaysia, 98000, Miri, Sarawak, Malaysia; 2 Clinical Research Centre Miri, Ministry of Health Malaysia, 98000 Miri, Sarawak, Malaysia

**Objectives:** Gentamicin is commonly administered using single-daily dosing (SDD) or conventional dosing (CD) regimens for adult orthopaedic patients. However, in our practice, target serum concentrations are sometimes not attained. This study aims to evaluate adherence to gentamicin dosing recommendation, examine the serum concentrations achieved with different initial dosing regimens, and assess target attainment in subsequent samples.

**Methods:** We reviewed the therapeutic drug monitoring (TDM) registry for all adult orthopaedic patients treated with gentamicin who had their TDM analysed from January 2016 to April 2022 at Miri Hospital. Patient demographics and steady-state gentamicin concentrations were collected from TDM request forms, the electronic laboratory database, and the Pharmacy Information System. The data were analysed using descriptive statistics and Pearson's chi-square test.

**Results and Discussion:** This study included 293 samples from 218 patients. The study found that 95.5% of patients received SDD below the recommended creatinine-based dosing. In the CD group, 55.6% of patients were given initial doses lower than 1.5 mg/kg/dose. Approximately 45.5% of SDD and 57.1% of CD initial samples achieved suboptimal gentamicin concentrations. Subsequent samples demonstrated that 80.0% achieved target concentrations. Among patients with normal renal function, 12-hourly dosing showed the highest likelihood of reaching target levels compared to 8-hourly dosing (χ^2^ = 95.58(1), *P *< 0.001).

**Conclusions:** The study highlights opportunities to improve gentamicin dosing practices to achieve target serum concentrations in adult orthopaedic patients. Findings suggest that 12-hour dosing is more effective in conventional regimens and emphasises the need to initiate with doses of at least 1.5 mg/kg/dose. The implementation of TDM recommendations further enhanced target attainment in the subsequent samples.

**Keywords**: gentamicin, peak, trough, aminoglycoside, monitoring

# Abstract: Evaluation of Knowledge and Use of Sublingual Glyceryl Trinitrate in Coronary Artery Disease Patients

**Authors:** Ling Weng Wong, Mei Chi Lee, Shirlie Chai, Michelle Lee Lee Ting, Wei Yang Khor

**Affiliations:** 1 Pharmacy Department, Miri Hospital, Ministry of Health Malaysia, 98000, Miri, Sarawak, Malaysia; 2 Clinical Research Centre Miri, Ministry of Health Malaysia, 98000 Miri, Sarawak, Malaysia; 3 Bintulu Health Clinic, Ministry of Health Malaysia, 97000 Bintulu, Sarawak, Malaysia

**Objectives:** Sublingual glyceryl trinitrate (SLGTN) is commonly prescribed for individuals with coronary artery disease (CAD). However, knowledge and use of SLGTN may be suboptimal.

This study aims to evaluate the knowledge and use of SLGTN among CAD patients and the factors associated with appropriate SLGTN use.

**Methods:** Using a convenience sampling method, we conducted this cross-sectional study among adult CAD patients treated with SLGTN and admitted to medical wards from November 2019 to May 2024 at Miri Hospital. The participants were interviewed using a validated, investigator-assisted questionnaire to assess the demographics (7 items), knowledge (8 items), and use (6 items) of SLGTN. One point was awarded for each correct knowledge response, while a score of zero was given for incorrect responses or if the participants answered “do not know”. The maximum knowledge score was 8. The knowledge categories included poor (scores 0–3), moderate (scores 4–6), and high (scores 7–8). For SLGTN use, the maximum score was 6, and a score of 5 and above denoted appropriate use. Data analysis encompassed descriptive statistics, and multiple logistic regression was performed to determine factors associated with appropriate SLGTN use.

**Results and Discussion:** This study included 314 SLGTN users, the majority of whom were male (67.5%), identified as Iban (37.9%), received primary school education or below (51.9%), had been using SLGTN for more than three months (75.8%) and had been counselled in the past six months (75.2%). The mean knowledge and use scores were 3.08 (SD = 1.25) and 4.68 (SD = 1.25). About 64.0% of patients (n = 201) possessed poor knowledge, and a third of the participants (n = 117) misused SLGTN. Male users, those with tertiary education, higher knowledge scores, and who had been counselled in the past six months were more likely to use SLGTN appropriately.

**Conclusions:** This work reveals opportunities for improvement in SLGTN education and highlights essential factors to further optimise SLGTN use among adults living with CAD. The findings suggest that targeted educational interventions could significantly enhance the appropriate use of SLGTN, particularly by improving educational outreach to those with lower educational attainment and ensuring regular counselling sessions.

**Keywords**: Nitroglycerin, nitrate, sublingual, ischemia, cardiac

# Abstract: Factors Affecting the Pharmaceutical Supply Chain Management Among Hospital Staff in the Selected Tertiary-Government Hospital in La Union

**Authors:** Sheila Mae V. Pulido, Faller, M. Erwin

**Affiliations:** 1 Graduate School, St. Bernadette of Lourdes College, Quezon City, Manila, Philippines; 2 Department of Pharmacy, Ilocos Training and Regional Medical Center, San Fernando City, La Union, Philippines; 3 College of Allied Health Sciences, Holy Child Central Colleges, Inc., Philippines

**Objectives:** This study aimed to identify critical factors influencing pharmaceutical supply chain management among hospital staff at selected tertiary government hospitals in La Union, Philippines. It also assessed the demographic profiles of staff, evaluated logistical processes, procurement practices, and inventory management, and explored the relationships between these factors and staff characteristics.

**Methods:** A correlational research approach was used, involving 135 hospital staff engaged in supply chain management, including procurement officers, supply officers, pharmacists, and nurses. Data were analyzed using multivariate multiple regression.

**Results and Discussions:** Most of the staff were aged 31–35, predominantly female with Bachelor's degrees, employed for 2–5 years, and had 6–10 years of experience. Nurses made up 22.22% of the participants. The study found high satisfaction in the accessibility (3.29) and availability (3.10) of logistical processes, though stock-level management needed improvement. Procurement processes were adequate, with solid performance in cost-effectiveness (3.47) and lead time management (3.34). Inventory management was effective in demand forecasting (3.09) and inventory turnover (3.16), but stock-level management required attention (2.81).Significant relationships were observed between demographic factors and supply chain management perceptions. Notably, staff with 2–5 years of service had a significant negative relationship with availability (B = −2.8, *p* = 0.013) and inventory turnover (B = −2.367, *p* = 0.04), but a marginally significant positive relationship with inventory turnover effectiveness (B = 5.217, *p *= 0.088). The role of the inventory officer showed a marginally positive relationship with availability (B = 2.033, *p *= 0.053) and was significant for better stock-level management (B = 3.3, *p *= 0.027). Younger staff (21–25 years) perceived more issues with timeliness (B = −8.456, *p *= 0.019).

**Conclusions:** Pharmaceutical supply chain management at these hospitals is effective but requires targeted improvements, especially regarding timeliness and inventory turnover. Recommendations include advanced inventory management systems, predictive analytics, and tailored training programs. Future research should explore specific interventions and technological advancements to optimize supply chains.

**Keywords:** pharmaceutical supply chain management, logistical processes, procurement, inventory management, hospital staff, tertiary hospital

# Abstract: Health Seeking Behavior And Compliance Of HIV Infected Individual To Antiretroviral Therapy

**Authors:** Jesa Sabillano Madelo

**Affiliations:** Tagum Doctors College Inc. Tagum City Philippines, Southwestern University, Cebu City, Philippines

**Objectives:** This study aimed to establish whether adherence to antiretroviral therapy (ART) among patients taking antiretroviral drugs and the health-seeking behaviour of patients living with HIV is associated with their socio-demographic characteristics.

**Methods:** This study utilized a descriptive correlational design utilizing both quantitative and qualitative approaches to determine the socioeconomic and demographic profile and the level of compliance among HIV-infected individuals receiving antiretroviral therapy medication appointments and medication—likewise, their level of health-seeking behaviour.

**Results and Discussion:** The findings of the study revealed that the stable increase in HIV cases in the country may have been due to non-compliance among HIV-infected individuals to medication and social life modification. As such, the study focused on testing the association of demographic characteristics of patients receiving antiretroviral therapy with the factors affecting their compliance with medication appointments and treatment. It was also revealed that most of the respondents are male within the age ranges of 19–35 years old with educational attainment of college level, single, jobless with family income of Php 5000 below. Results revealed that stipulated factors hamper their requirements to regularly make appointments with their physician to check their health status. Also, most of the HIV patients were compliant with the treatments they required. However, the side effects of the medication were found to have a high probability that these patients may not comply with the treatment.

**Conclusions:** This study emphasizes the need for targeted interventions that consider the economic, educational, and social backgrounds of individuals living with HIV. Programs should focus on providing economic support, enhancing education about HIV management, and fostering robust community and familial support systems to improve health outcomes and quality of life.

**Keywords**: Antiretroviral therapy, compliance, HIV, Health seeking behaviour

# Abstract: Determinants of Diarrheal Disease and Patterns of Antibiotic Use Among Children Under Five in Indonesia: Insights from the 2017 Demographic Health Survey

**Authors:** Tashya Dhela Maylinda, Rivai Endra Dwi Yulianto

**Affiliations:** 1 Faculty of Pharmacy, Universitas 17 Agustus 1945, Jakarta, Indonesia; 2 National Agency of Drug and Food Control, Jakarta, Indonesia

**Objectives:** Diarrhea is still the second leading cause of death for children under five years of age. Deaths of children under five years due to diarrhoea are caused by unsafe water, inadequate sanitation, and hygiene. Socio-demographic characteristics, environment, behaviour, and parenting practices are associated with diarrhoea in children under five years in Indonesia. The use of antibiotics in the treatment of diarrhoea needs attention. Antibiotic resistance can occur if its use is not based on a proper diagnosis. This study examined the factors that influence the incidence of diarrhoea in children under five years of age. This study also looked at the prevalence of treatment centres for diarrhoea patients and the use of antibiotics to treat diarrhoea in children under five.

**Methods:** The data used is cross-sectional data on children under five living in households in Indonesia taken from the 2017 DHS survey through data collections and survey reports. Descriptive analyses were performed on survey reports and datasets related to demographics, the prevalence of diarrheal diseases, and the use of antibiotics to treat diarrhoea. Significance analysis was conducted to see whether the selected variables affected the incidence of diarrhoea in children under five years of age.

**Results and Discussion:** Puskesmas is a place to seek treatment for diarrhoea, and it is most frequently visited by children under five years old (20.99%). 10.9% of pediatric patients with diarrhoea visited more than one health facility. Private hospitals are the places that most often give antibiotics to children under five who have diarrhoea (25%). Antibiotics were given by licensed healthcare facilities and other places visited by respondents, namely traditional birth attendants, grocery stores, and other sources.

**Conclusions:** Gender, age, location of residence (urban or rural), wealth level, mother's education level, and the primary material of the house floor are significant factors influencing the incidence of diarrhoea in children under five years.

**Keywords**: Diarrhea, Prevalence, Antibiotics, DHS 2017, Indonesia

# Abstract: Formulation and Stability Test of Coriander Seeds (***Coriandrum sativum***) Extract in 96% Ethanol, n-Hexane Fraction and Ethyl Acetate Fraction Hair Tonic and Hair Growth Activity by In Vivo on White Rabbit (***New Zealand White***)

**Authors:** Linawaty, Diana Novita Sari, Liandhajani

**Affiliations:** Faculty of Pharmacy, 17 Agustus 1945 Jakarta University, Jakarta, Indonesia

**Objectives:** Hair tonics address hair loss due to prolonged contact with the scalp, unlike other hair care products. Herbal plants, like coriander seeds (*Coriandrum sativum*), are traditionally known for their therapeutic properties, including promoting hair growth. This study aims to evaluate the effectiveness of hair tonic formulations containing coriander seed extracts and fractions in promoting hair growth, compared to a minoxidil 2% control.

**Methods:** Coriander seeds were extracted using 96% ethanol, producing 133.43 grams thick extract. Hair tonic formulations were developed in ethanol extract, n-hexane fraction and ethyl acetate fraction, each in concentrations of 2.5%, 5.0% and 7.5%. Minoxidil 2% was a positive control, while a base-only tonic was a negative control. The formulations were tested on white rabbits. The pH and density of each formulation were measured. The stability test of each formulation was tested by the accelerated stability test method in temperatures at 2–4°C and 40°C for six cycles to check the physical stability results. Hair growth was assessed using Shapiro-Wilk and One-Way ANOVA tests.

**Results and Discussion:** The pH values of the formulation ranged from 4.64 to 5.92, falling within the acceptable range for hair tonics. Hair growth assessments indicated that the ethyl acetate fraction, mainly at 7.5%, was nearly as effective as the Minoxidil 2% control.

**Conclusions:** Coriander seed extract, especially ethyl acetate 7.5% fraction, demonstrates significant potential in promoting hair growth, making it a promising alternative to traditional treatments like Minoxidil.

**Keywords**: *Coriander sativum*, hair tonic, hair growth, herbal extract, Minoxidil

# Abstract: Healing Activity of Ethyl Acetate Fraction from Carica papaya L. Leaves on Duration and Bruise Area in Male Wistar Rats

**Authors:** Fitriani, Rizki awaluddin, Alisa Khadijah, Lutfi Chabib

**Affiliations:** 1 Department of Pharmacy, Faculty of Pharmacy, University of 17 Agustus 1945 Jakarta, Jakarta, Indonesia; 2 Department of Pharmacy, Faculty of Mathematics and Natural Sciences, Islamic University of Indonesia, Yogyakarta, Indonesia

**Objectives:** The study aimed to investigate the effect of various concentrations of ethyl acetate fraction from Carica papaya L. leaves on the bruise area and duration.

**Methods:** Papaya leaves (Carica papaya L.) were extracted using methanol and subsequently fractionated into three levels based on polarity (n-hexane, ethyl acetate, and methanol-water). The bruised model was established on the hind legs of rats by applying 25 blows with a 100-gram metal rod from a height of 20 cm. Bruises were observed for 24 hours post-induction. Extracts and fractions were identified using thin-layer chromatography to determine the presence of compound classes. The ethyl acetate fraction treatments were divided into three groups: 1000 ppm, 2000 ppm, and 4000 ppm. Thrombopop gel (containing Heparin Sodium 200 IU/gram) was used as a positive control, while a gel base was a negative control. The bruised area was measured every 24 hours for seven days, and the bruise duration was observed until the skin colour returned to normal. The colour change was matched based on usual and bruised skin colour, referring to the RGB code.

**Results and Discussion:** Our findings reveal that the ethyl acetate fraction presents more compounds than the n-hexane and methanol-water fraction. The bruised area in the treatment groups with ethyl acetate fraction significantly differed from the negative control group (*p*<0.05). The group treated with 4000 ppm ethyl acetate fraction showed a smaller bruised area than the 1000 ppm and 2000 ppm concentrations (*p* < 0.05). The bruise duration in the group treated with a 4000 ppm ethyl acetate fraction significantly differed from the negative control (*p *< 0.05).

**Conclusions:** The ethyl acetate fraction of Carica papaya L. leaves effectively reduced the duration and bruise area in the bruise model on rats.

**Keywords**: Carica papaya L., Ethyl acetate fraction, Bruise (ecchymosis), healing activity, rat model of hypertension

# Abstract: The Relationship Between Linezolid Therapy and Haemoglobin (Hb) Levels In Drug-Resistant Tuberculosis (DR-TB) Patients

**Authors:** Farisa Luthfiana, Yulyana Nur Hidayah, Dini Permata Sari

**Affiliations:** Faculty of Pharmacy, Universitas 17 Agustus 1945 Jakarta, Indonesia

**Objectives:** Linezolid is a selective antibiotic commonly used for treating drug-resistant tuberculosis (TB) patients. The recommended duration for linezolid therapy in cases of drug-resistant TB is a minimum of 6 months. Long-term use of linezolid has been associated with bone marrow suppression, potentially reducing haemoglobin (Hb) levels. This study aims to evaluate the relationship between haemoglobin levels and linezolid in patients with drug-resistant tuberculosis (DR-TB) within six months.

**Methods:** This investigation utilized a retrospective analytical observational study with a cross-sectional design. Data were obtained from patient medical records by evaluating haemoglobin levels at initiating linezolid therapy and at the 6-month follow-up. The study sample included 32 patients selected through total sampling. The inclusion criteria encompassed individuals diagnosed with drug-resistant TB who were undergoing linezolid therapy, while exclusion criteria comprised patients who were receiving iron supplementation or those with other conditions associated with anaemia.

**Results and Discussion:** The average haemoglobin level at the initial therapy was 12.00 ± 1.75 g/dL, and the 6-month follow-up was 11.7 ± 2 0.28 g/dL. The statistical analysis yielded a *p*-value of 0.396, indicating no significant association between linezolid treatment and changes in haemoglobin levels among the patients.

**Conclusions:** Administration of linezolid within six months does not significantly impact haemoglobin levels in drug-resistant tuberculosis (DR-TB) patients.

**Keywords**: Linezolid, Haemoglobin, DR-TB.
